# Crystal Clear: Decoding Isocyanide Intermolecular Interactions through Crystallography

**DOI:** 10.1021/acs.joc.3c02038

**Published:** 2024-01-04

**Authors:** Eleftheria Chatziorfanou, Atilio Reyes Romero, Lotfi Chouchane, Alexander Dömling

**Affiliations:** † Innovative Chemistry Group, Institute of Molecular and Translational Medicine, Faculty of Medicine and Dentistry and Czech Advanced Technology and Research Institute, Palacky University in Olomouc, Olomouc 779 00, Czech Republic; ‡ Genetic Intelligence Laboratory, Weill Cornell Medicine-Qatar, Qatar Foundation, P.O. Box 24144, Doha, Qatar; § Department of Microbiology and Immunology, Weill Cornell Medicine, New York 10021, United States; ∥ Department of Genetic Medicine, Weill Cornell Medicine, New York 10021, United States

## Abstract

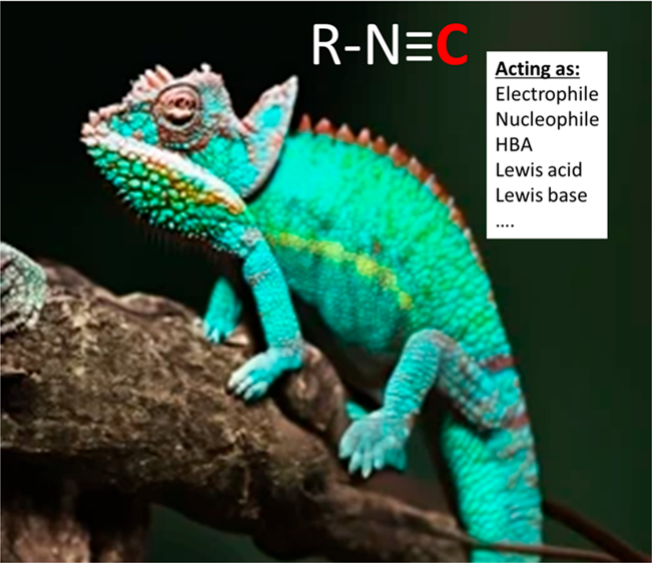

The isocyanide group is the chameleon among the functional groups in organic chemistry. Unlike other multiatom functional groups, where the electrophilic and nucleophilic moieties are typically separated, isocyanides combine both functionalities in the terminal carbon. This unique feature can be rationalized using the frontier orbital concept and has significant implications for its intermolecular interactions and the reactivity of the functional group. In this study, we perform a Cambridge Crystallographic Database-supported analysis of isocyanide intramolecular interactions to investigate the intramolecular interactions of isocyanides in the solid state, excluding isocyanide–metal complexes. We discuss examples of different interaction classes, including the isocyanide as a hydrogen bond acceptor (RNC···HX), halogen bonding (RNC···X), and interactions involving the isocyanide and carbon atoms (RNC···C). The latter interaction serves as an intriguing illustration of a Bürgi–Dunitz trajectory and represents a crucial experimental detail in the well-known multicomponent reactions such as the Ugi- and Passerini-type mechanisms. Understanding the spectrum of intramolecular interactions that isocyanides can undergo holds significant implications in fields such as medicinal chemistry, materials science, and asymmetric catalysis.

## Introduction

Understanding how matter interacts microscopically is key to designing the macroscopic properties of new materials. At the atomic level, molecules can engage in either covalent or noncovalent bonds, which govern various aspects such as strength, orientation, distance, and numerous other characteristics that ultimately translate into macroscopic features. In particular, noncovalent interactions play a key role in organic and supramolecular chemistry, biochemistry, and catalysis with applications in synthesis, materials, and drug discovery. Comprehending and effectively manipulating these interactions through synthesis are crucial to mastering the rational design of new materials with enhanced properties. By gaining a thorough understanding of these interactions, we can strategically engineer materials that exhibit improved characteristics and functionalities.

Motivated by our enduring interest in isocyanide reactivity and their applications in organic synthesis and medicinal chemistry, this study aims to analyze published crystal structures of isocyanides, to uncover valuable insights into their structural features and interactions.^1−4^ We examined the crystal structures of isocyanides available in the Cambridge Structural Database (CSD), specifically excluding metal complexes. In exploring this data set, we focused on several questions. What kind of noncovalent interactions can isocyanides undergo, beyond metal coordination? What unique characteristics do isocyanides exhibit compared to other functional groups? How can this knowledge be applied to elucidate the unique chemical reactivity? We categorized the types of interactions observed and interpreted our findings using qualitative frontier orbital models. By doing so, we aim to provide a comprehensive understanding of the distinct properties and behaviors of isocyanides in terms of noncovalent interactions.

The CSD is a unique and comprehensive repository, containing >1.2 million meticulously curated and verified three-dimensional (3D) structures. This invaluable resource serves as a vital tool for investigating intra- and intermolecular interactions.^5^ The vast data set predominantly consists of organic compounds (43%) and organometallic compounds (57%), derived from X-ray crystallography experiments. Notably, the CSD encompasses a diverse range of functional compounds, including metal–organic frameworks (MOFs), catalysts, pigments, agrochemicals, and an extensive collection of drug and pharmaceutical crystal structures. Leveraging these extensive data, medicinal chemists can effectively analyze and visualize the inter- and intramolecular contacts between functional groups within small organic molecules.^6−8^ This analysis yields insights into functional group reactivity, facilitates the validation of configurational changes in complex biomolecules across various organic solvents, and plays a pivotal role in structure-based drug design (SBDD) for developing lead compounds with improved pharmacodynamic properties.^9^ To facilitate rapid data access and analysis in computer-aided drug design (CADD), the CSD provides a suite of useful software tools.

**Figure 1:**
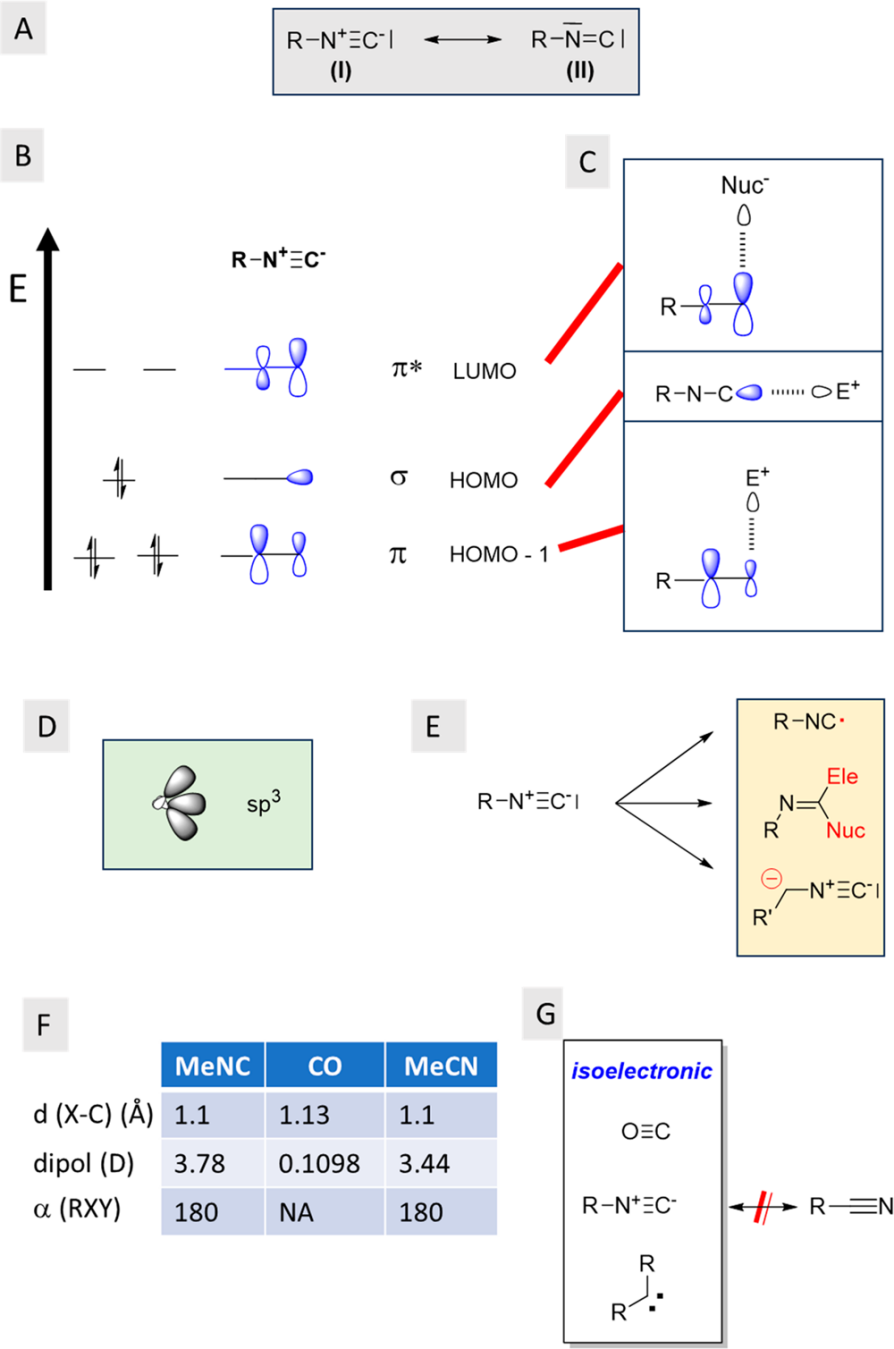
Isocyanide structure and properties. (A) Mesomeric structures of the isocyanide. (B) Frontier orbitals of isocyanides. (C) Major predicted intermolecular interactions. (D) sp^3^-type hybrid orbital of isocyanide C. (E) Three major reaction pathways of isocyanides. (F) Some physicochemical properties of MeNC and related compounds. (G) Carbon monoxide and carbene are isoelectronic to the isocyanide; however, the isomeric nitriles are not.

**Figure 2:**
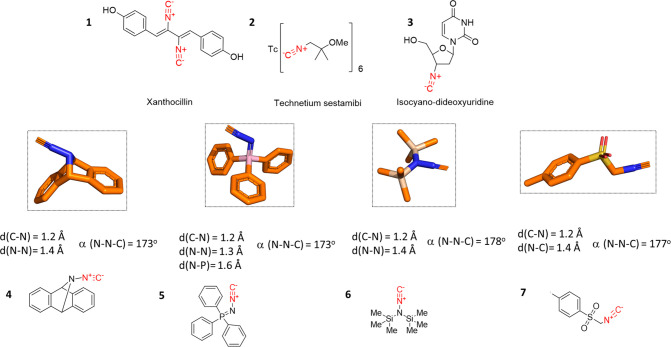
Structures of some significant isocyanides (**1**–**3**) and unusual *N*-isocyanides. Comparison of the 3D structures of *N*- and *C*-isocyanides. Isocyanide **4** (RECPUN, CCDC 1566922) shows coplanarity around the nitrogen adjacent to the isocyanide N. In contrast, the nitrogen adjacent to the isocyanide N in compound **5** (SAZPUF, CCDC 279551) has a trigonal pyramidal conformation. *N*-Isocyanide **6** (KEPPOM, CCDC 917995) features a trigonal planar N. Solid state conformation of tosmic isocyanide **7** (LUKVUK, CCDC 1063415).

The first isocyanide was discovered and described in 1859 by Liecke, although the structure was initially mis-interpreted.^10^ The electronic structure of isocyanides is often described by two mesomeric structures (Fg.1A), including a triple bond between C and N and formally charged N^+^ and C^–^ (I) and a carbene-type structure involving a C=N bond with a N lone pair and a sextet C (II). Isocyanides are therefore isosteric and isoelectronic with carbon monoxide and carbenes but substantially differ from the isomeric nitriles (Figure 1G). Crystallographic analysis reveals that the C–N distance of isocyanides corresponds to a triple bond (Figure 1F). The N–C distance, for example, in toluenesulfonylmethyl isocyanide (tosmic) **7**, is 1.2 Å. The isocyanide generally exhibits a linear geometry; e.g., in tosmic **7**, the C–N–C angle is 177° (Figure 2).^11^

Isocyanides, also sometimes called isonitriles, show a very rich organic chemistry that is based on their unusual reactivity and has been comprehensively reviewed.^12−14^ The key reactivities include their α-acidic character (although less acidic than nitriles) featuring a rich heterocyclic chemistry,^15^ their ease of radical formation, and, most unusually, their C-centered reactivity toward electrophiles and nucleophiles. They serve as key building blocks in isocyanide-based multicomponent reactions (IMCRs) like van Leusen, Ugi, Passerini, and the related Groebke–Blackburn–Bienaymé,^16^ allowing for the efficient generation of chemical libraries and small molecule scaffolds in a one-pot fashion that recently have found widespread applications in DNA-encoded library synthesis.^17^ MCRs are also characterized by their scalability and minimal solvent volumes and contribute to the green and sustainable synthesis of diverse compounds as recently reported.^18^ While nitriles have been extensively explored in medicinal chemistry and the accessibility of heterocycles, isocyanide-containing molecules have surprisingly remained unexplored. For instance, the ChEMBL database contains a substantial number of nitrile-containing compounds, accounting for a significant percentage of the total bioactive compounds recorded; however, isocyanides constitute a mere 0.03% of the recorded compounds.^19^ Similarly, within the Protein Data Bank (PDB), we conducted substructure searches to identify structures within the PDB that contained isocyanide functional groups. To define the isocyanide moiety, we employed the SMARTS pattern “*N#C”. These searches were also restricted to atoms labeled as “LIGAND” to maintain a precise focus on ligand entities within the PDB. We used the CSD Python API to perform these searches, specifically utilizing the .SMARTSSubstructure method within the ccdc.search module,^20^ although a very small fraction of the deposited protein structures (23 of 177 204) feature cocrystallized isocyanide-related molecules.

The structures of several significant isocyanides and products are shown in Figure 2. Surprisingly, despite their bad reputation based on their often malodor, two isocyanides are drugs; natural product-derived xanthocillin **1** was previously used as a topical antibiotic,^21^ and technetium (^99m^Tc) Sestamibi **2** is used in cardiac, parathyroid, and breast imaging.^22^ It is noteworthy that xanthocillin **1** displays activity even against multidrug resistant strains while exhibiting low toxicity to human cells. The mechanism of action of xanthocillin **1** working by inhibition of iron-bound heme has recently been elucidated.^23^ Of interest is also antiviral isocyano AZT derivative **3**, which was first synthesized by Ugi.^24,25^ Tosmic **7** is an isocyanide produced on a large scale in hundreds of tons per year, being a key building block in the synthesis of multiple commercial products.^11,26,27^ Isocyanides are present as a structurally diverse group of natural products, isolated from marine organisms, microorganisms, and fungi.^28^ Surprisingly, it was discovered using genome mining that isocyanides are the fifth-largest class of natural products produced by fungi, opening an untapped treasure trove.^29^ Thus, isocyanides hold promise for interesting new applications beyond synthesis in different areas, including medicine and materials. The medicinal chemistry of isocyanides has recently been comprehensively reviewed.^30−34^

A great majority of isocyanides are *C*-isocyanides, where the isocyano group is bound to an aliphatic or aromatic C (Figure 2). However, *N*-isocyanides are also known, where the isocyano group is bound to a N (Figure 2); on the contrary, in *N*-isocyanides **4** and **5** the isocyanide-bound N is pyramidal (sp^3^) and the bis-trimethylsilyl substituted N in **6** is trigonal planar (sp^2^).^27,35,36^ It is noteworthy that isocyaniminotriphenylphosphorane **5**, another *N*-isocyanide, is a synthetically very useful isocyanide that can undergo a rich chemistry leading to a plethora of heterocycles through an initial MCR followed by aza-Wittig-type intramolecular ring closures.^37^ Smaller isocyanides have been detected in space and are discussed as being relevant to the origin of life.^38,39^

## Results and Discussion

### What Are the Special Electronic Features of Isocyanides?

Understanding the electronic properties of isocyanides is crucial for comprehending their interactions and synthetic reactivity (Figure 1B,C). The frontier orbital theory offers a qualitative tool to do so, while more sophisticated descriptions based on high-level calculations are available to the interested reader.^40−42^ The primary synthetic feature of isocyanides is their amphiphilic ability to engage in reactions with nucleophiles and electrophiles at the carbon atom. This behavior gives rise to α-adduct formation through the addition of nucleophiles and electrophiles (Figure 1E). Isocyanides possess a unique combination of a σ-type lone pair at the carbon, serving as an electron pair donor (nucleophile, Lewis base), an energetically accessible π-CN orbital pair (HOMO–1) with a large orbital coefficient on the C, and a low-energy pair of π*-CN orbitals again with the largest orbital coefficient on the C, functioning as an acceptor partner (electrophile, Lewis acid). Hence, depending on the electronic characteristics of the interacting partner and its trajectory, isocyanides can act as either donors or acceptors in intramolecular interactions and have been also called molecular chameleons.^43^ Because of the frontier orbitals, isocyanide C is expected to act as a hydrogen bond acceptor or nucleophile through its HOMO σ orbital (Figure 1C). Moreover, the HOMO–1 π orbital with a large orbital coefficient on the C can be expected to act as a hydrogen bond acceptor, however, in an orthogonal direction. In contrast, the π*-CN orbitals with the largest orbital coefficient on the C are expected to work as an electrophile (Figure 1C). Applying hybrid orbitals of the π and σ frontier orbitals to generate an sp^3^-type orbital is also useful for understanding the donor and acceptor behavior of the isocyanide C (Figure 1D). Examples of isocyanide behavior in the solid state that can be rationalized by the frontier orbitals are described below.

### Query Definition

**Figure 3:**
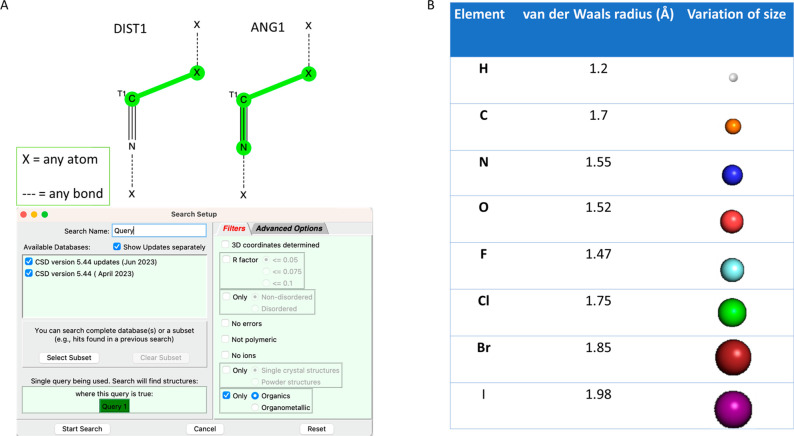
CSD query and van der Waals radii investigated. (A) Screenshot of the query criteria of our investigation. All organic isocyanides that produce interactions below the van der Waals radii and the angle α(N–C-X) distribution are included in the query. The X parameter represents any atom, and dashed lines represent any bond. (B) van der Waals radii of all of the elements in which we identified isocyanide contacts and their sizes.

To conduct our crystallographic analysis, we utilized the most up-to-date version of the CSD database (June 2023.1). We focused on analyzing interactions that involved isocyanide C and its neighboring atoms, specifically those with distances that are shorter than the sum of the van der Waals radii. We deemed such interactions productive and worthy of investigation, as such distances indicate binding interactions. Our search targeted isocyanides in which the immediate neighbor atom of the isocyanide C was within 3.7 Å. Figure 3 illustrates the detailed query definition. For the purpose of our discussion, we excluded isocyanide–metal complexes, which are highly significant in metal–organic chemistry but of minor relevance to our analysis. Thus, our query includes all of the organic isocyanides that form interactions with any other atom, except metals, below the sum of the van der Waals radii (X···CN-R). The nitrogen of the isocyanide can be bonded to any non-metal atom (parameter X), and the bond between them can be any bond. Also, we specified the connectivity order of carbon. Finally, in the parameters, we have excluded organometallics and isocyanides that form interactions above the van der Waals radii.

**Figure 4:**
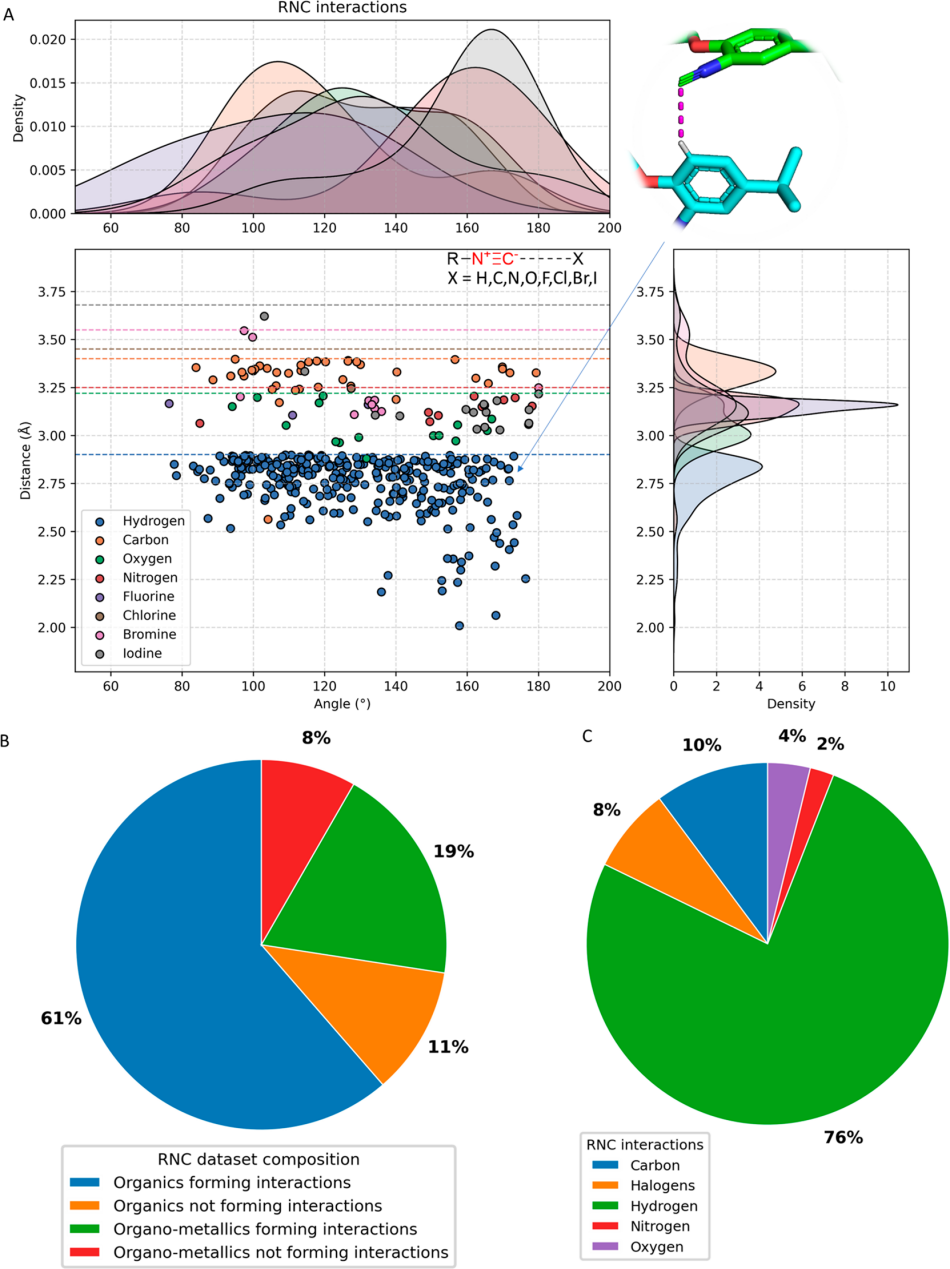
Summary of the query results. (A) Scatter plot (RNC···X distance, angle) and density of states (DOS) of the interactions between RNC and any neighbor atoms at a distance of ≤3.7 Å. (B) Distribution of the main classes of isocyanides in the CSD. (C) Classification of the observed interactions of the isocyanides.

The CSD contains a total of 277 isocyanides; 76 of them are metal complexes. Among the remaining isocyanide complexes (207), 170 displayed a nearest neighbor atom distance to the isocyanide C of ≤3.7 Å (Figure 4B). The Supporting Information provides a comprehensive list of all non-metal isocyanide complexes. The distance angle scattered plot of the query results (Figure 4A) indicates that the largest number of isocyanide–C interactions are hydrogen bonds, followed by short C–C, −N, −O, and −halogen interactions.

In the following sections, we present various examples of these complexes, showcasing different types of interactions along with our attempt to classify and interpret them.

### Hydrogen Bonding (RNC···HX)

**Figure 5:**
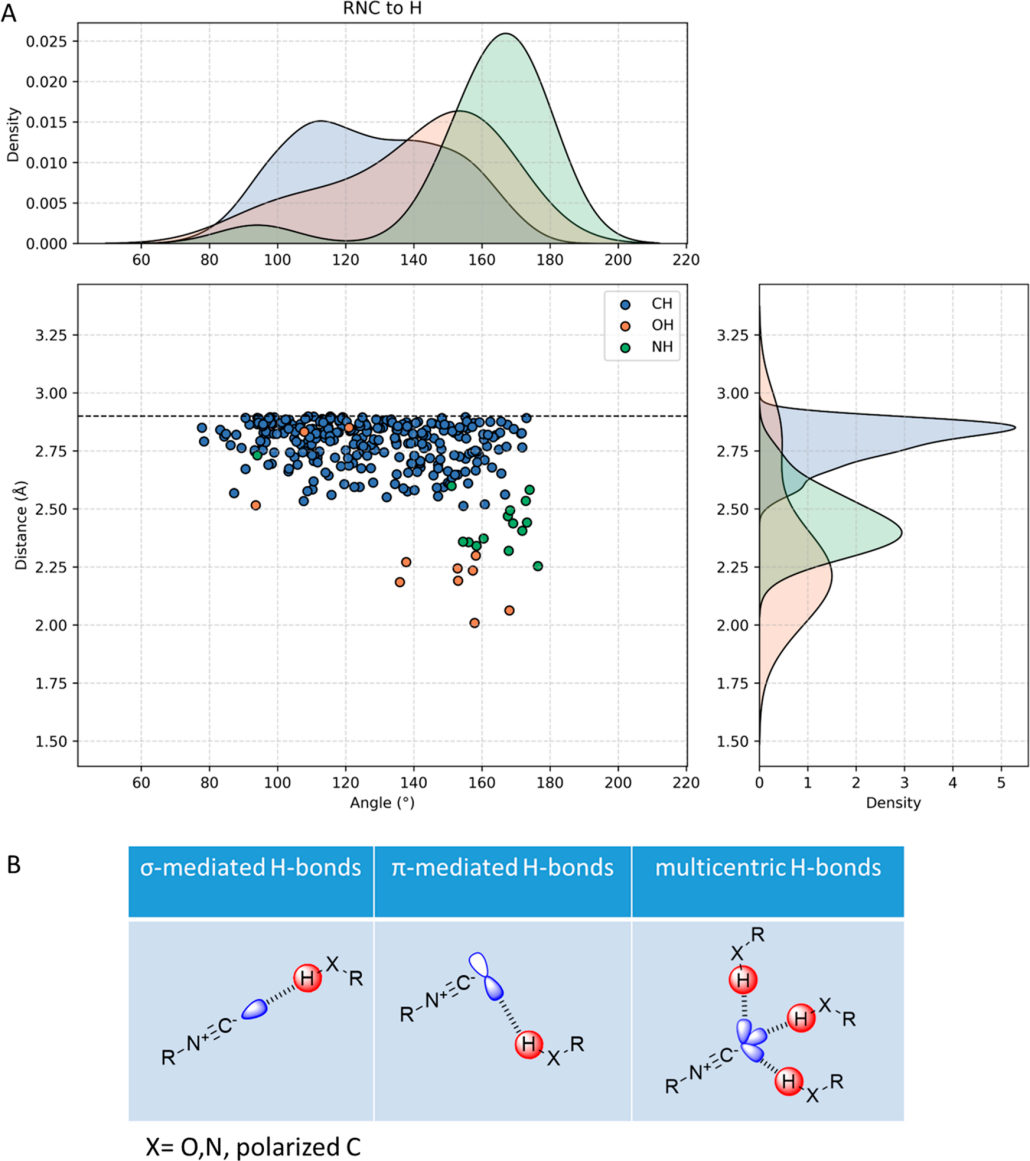
Overview of isocyanide C hydrogen bonds (RNC···HX). (A) Scatter plot of the isocyanide C interacting with a hydrogen bonded to an electronegative atom, such as oxygen, nitrogen, and carbon. The *x*-axis represents the distribution of α(N–C–H) angles, and the *y*-axis the distance between the carbon of the isocyanide and the hydrogen. The isobars corresponding to the sum of the van der Waals radii of H and C are shown as dotted lines. (B) Three classes of hydrogen bonding interactions with the isocyanide C. The major interacting RNC-based filled orbital is colored blue, and the interacting σ orbital of the -XH is colored red.

Hydrogen bonds play a fundamental role in supporting life on Earth, serving vital functions in biological structure, function, and conformational dynamics. While a simplistic definition of hydrogen bonds describes them as interactions between a hydrogen atom covalently bound to an electronegative donor and the lone pair of electrons of an acceptor, their actual nature is far more intricate. To gain a deeper understanding, interested readers are encouraged to refer to comprehensive reviews on the subject.^44−46^ Traditionally, nitrogen (N) and oxygen (O) are predominantly recognized as hydrogen bond acceptors, while carbon (C) is rarely associated with this function. However, isocyanides exhibit an intriguing property whereby the carbon atom possesses energetically accessible filled σ and π orbitals, enabling it to act as a hydrogen bond acceptor group (Figure 1B,C). Notably, in the RNC functional group, the acceptor site is located on the carbon atom rather than the more electronegative nitrogen atom. Considering the orientations of the C-centered filled orbitals, hydrogen bonding is expected to occur in two distinct directions (Figure 5B): (A) along the axis of RNC through the σ orbital and (B) perpendicular to the RNC axis through the C-centered π orbital. From an orbital hybridization perspective, an approximately sp^3^-type orientation could facilitate multipolar hydrogen bonding interactions involving up to three hydrogen bonding partners in a multipolar fashion.

Analyzing the CSD, we were surprised that the hydrogen bonding is the most abundant of all spotted interactions, accounting for 77% of all interactions. A vast majority of these hydrogen bonds appear to be to CH hydrogen, though the closest ones appear to be to OH and NH hydrogens. Probably, a large number of the observed CH hydrogen bonds are caused by the crystal packing effect. We found that the isocyanide C can form hydrogen bonds to hydroxyl -OH, amine -NH, or unpolarized and polarized -CH (Figure 5A). In our investigation, we spotted 322 isocyanides that form different hydrogen bonds, with distances of the carbon of the isocyanide and the hydrogen atom with which it interacts, *d*(XH), varying from 2.0 to 2.9 Å (Figure 5). Remarkably, a significant proportion of all non-metal isocyanides, 77% within our query, demonstrated the ability to form hydrogen bonds, seemingly a general phenomenon in isocyanide chemistry. This observation highlights the potential utility of isocyanides as ligands in medicinal chemistry, but its understanding can also facilitate the design of chiral ligands for catalysis or material design.

### Hydrogen Bonding (RNC···HO)

**Figure 6:**
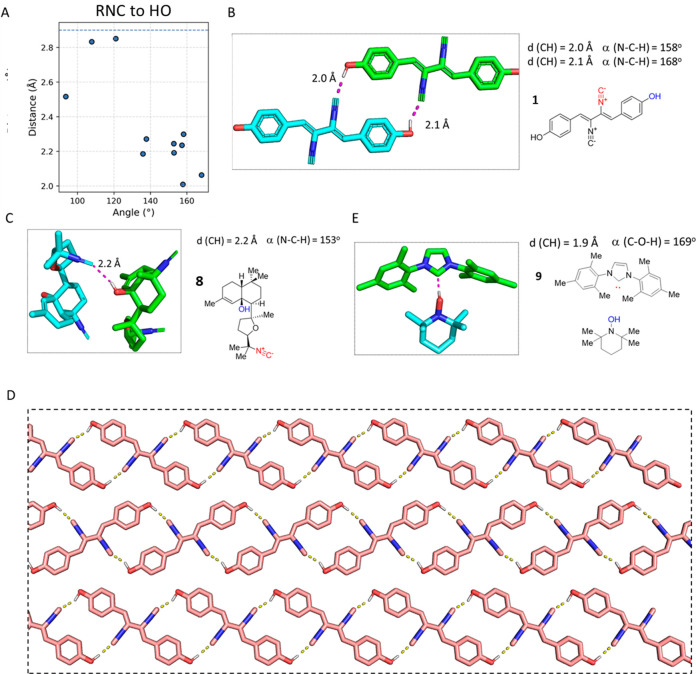
Examples of RNC···HO hydrogen bonds and comparison to isomorphic carbene C hydrogen bonds. (A) Scatter plot of all of the interactions between isocyanide C and OH. (B) Formation of two hydrogen bonds between the carbon atom of the isocyanide and the phenolic hydroxyl group of xanthocilin **1** (BAVHUB, CCDC 1106505). The carbon of the isocyanide acts as a hydrogen bond acceptor mediated by the σ orbitals. (C) In kalihinene diterpene **8** (HEXZIT, CCDC 1175506), the isocyanide interacts with the aliphatic hydroxyl group via the C-centered π and σ orbitals indicated at an NCH angle of 153°. (D) Infinite 2D sheet of xanthocillin mediated by four hydrogen bonds per molecule. (E) Carbene complex **9** (YENBUP, CCDC 299518) forming a hydrogen bond.

Eleven RNC···HO hydrogen bonds were found in the CSD (Figure 6A). An aromatic phenol included in an isocyanide C hydrogen bond is exemplified within the antibiotic natural product xanthocillin **1** (Figure 6B). The hydrogen of the hydroxyl group interacts with the carbon of the isocyanide at distances of 2.0 and 2.1 Å. This interaction clearly involves the σ orbitals of the isocyanide moiety, while the hydrogen bonding occurs at an α(N–C–H) angle of 153°, an extension of the RNC axis. The extended hydrogen bonding network in xanthocillin **1** is leading to the formation of an infinite two-dimensional (2D) sheet in the crystal lattice with the aromatic ring system all coplanar (Figure 6D), indicating their potential use as an element in noncovalent interactions in the design of new materials. Another interesting example of hydrogen bonds in isocyanides is seen in cytotoxic kalihinene **8**, a diterpene natural product isolated from the marine sponge *Acanthella cavernosa*. In this example, isocyanide **8** interacts through the C-centered π orbital and the σ orbitals with the aliphatic OH (Figure 6C).^47^ The interaction is formed at a distance *d*(CH) of 2.2 Å and an angle α(N–C–H) of 153°. Interestingly, the carbene C that is isoelectronic to the isocyanide C can also form OH···C hydrogen bonds. An example found in the CSD is electron poor imidazole-based **9** forming a very short 1.9 Å C···HO bond to the hydroxylamine partner at an angle (C–H–O) of 169° (Figure 6E).^48^

### Hydrogen Bonding (RNC···HN)

**Figure 7:**
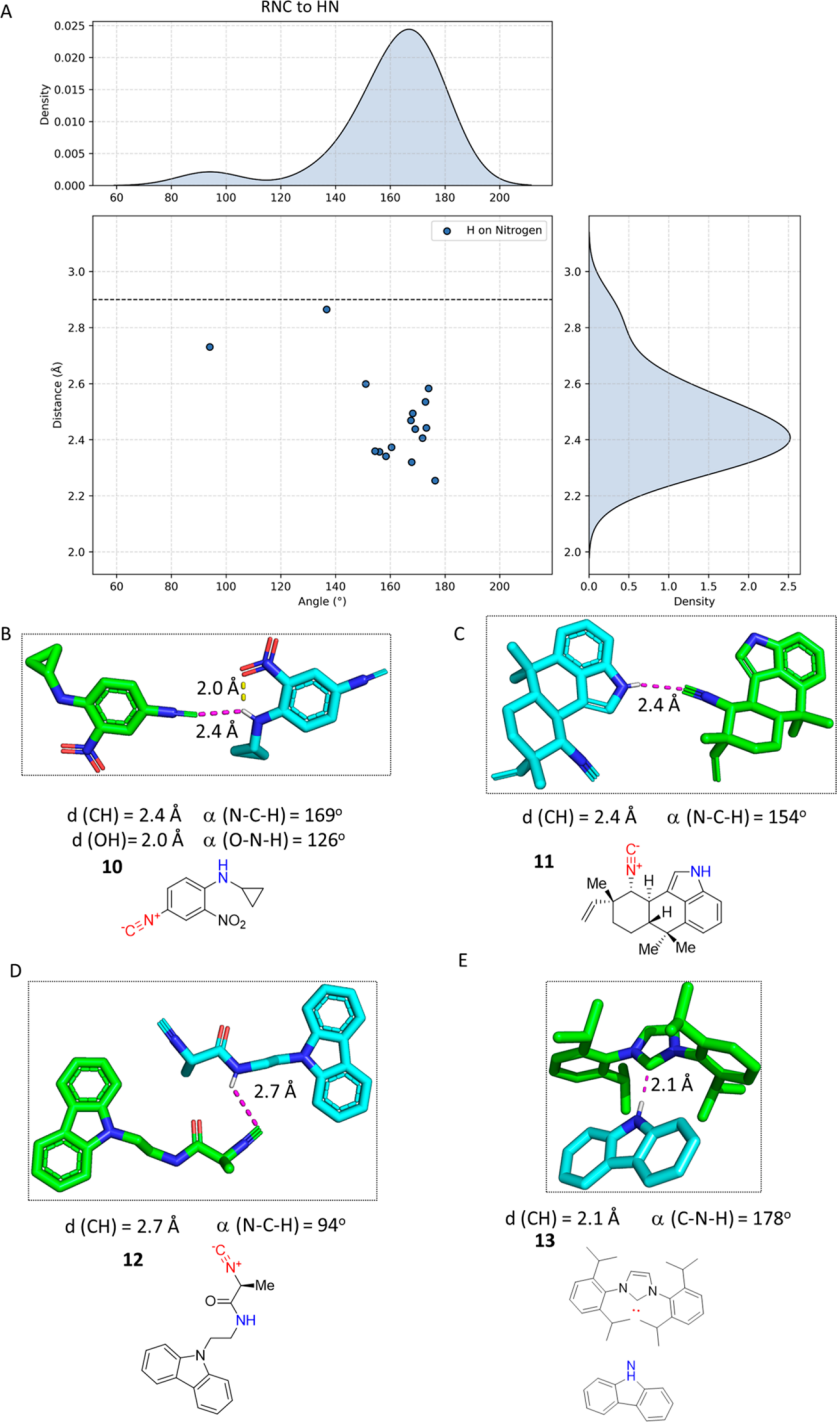
Examples of RNC···HN interactions and comparison to a carbene complex. (A) Scatter plot of all of the NH···CNR hydrogen bonds from CSD, including the isobar of the sum of the vdW radii of C and H as a blue dotted line. (B) *p*-Isocyano aniline **10** (LAVQUY, CCDC 2091122) next to the hydrogen bond coplanarity between adjacent phenyls. (C) The NH···CNR hydrogen bond in hapalindole H **11** (ROMNUE, CCDC 991945) is close to linear with all atoms being almost coplanar. (D) Isocyanide **12** (SUSYIP, CCDC 755663) exhibits a distinct behavior, approaching the hydrogen with the π orbital close to the rectangle, 94°. (E) Example of an isoelectronic carbene complex **13** (HOKSUY, CCDC 1901810) forming a NH···C hydrogen bond by engaging to the carbazole NH of 2.1 Å at an angle (CHN) of ∼180°.

Fifteen RNC···HN hydrogen bonds are detected in the CSD (Figure 7A). The RNC···HN group closely follows the OH···CNR binding principles. *p*-Isocyano aniline **10** forms a one-dimensional (1D) chain in the crystal mediated by an RNC···HN hydrogen bonding interaction (Figure 7B).^49^ The distance of 2.4 Å and the angle of NCH 169° suggest a clear σ orbital-mediated hydrogen bond. Interestingly, the *o*-nitro group forms a short intramolecular hydrogen bond of 2.0 Å to the NH, as well. The phenyl rings of the adjacent interacting molecules are coplanar, suggesting a certain degree of π electron delocalization. The complex, NF-κB inhibitory marine natural product hapalindole H **11**, produced by the *Stigonematales* genus of cyanobacteria, forms a hydrogen bond through the isocyanide C of one molecule with the indole NH of the neighboring molecule at a distance of 2.4 Å and an angle (NCH) of 154° (Figure 7C).^50^ The intriguing bioactivity diversity of the hapalindole isocyanide family of natural products has been comprehensively reviewed.^51^ Another example of a close RNC···HN crystal contact is isocyanide **12** (Figure 7D).^52^ In contrast to the previous examples, the isocyanide approaches the NH of the neighbor molecule involving the π orbital at a distance of 2.7 Å and an angle (NCH) of 94°. Overall, the angular distribution in the scattered plot suggests an α(NCH) preference of ∼180°. Similarly, to the hydroxyl hydrogen bond, a carbene complex to a HN is present in the CSD. Imidazole-derived carbene **13** forms a short bond to the cocrystallized carbazole NH of 2.1 Å at an angle (CHN) of ∼180° (Figure 7E).^53^

### Hydrogen Bonding (RNC···HC)

**Figure 8:**
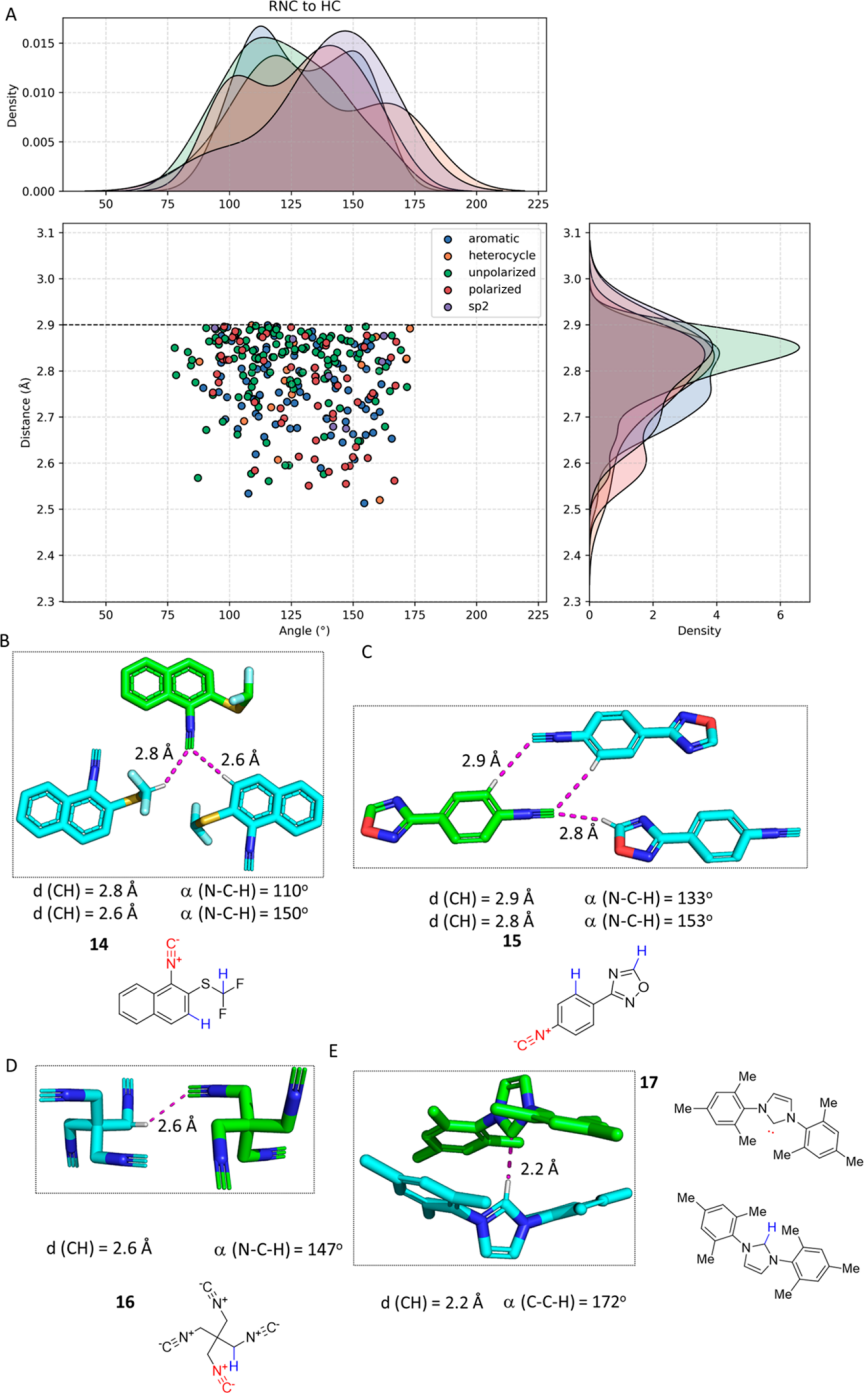
Examples of RNC···HC hydrogen bonds. (Α) Scatter plot of RNC···HC from CSD, including the isobar of the sum of the vdW radii C and H as a blue dotted line. (Β) Bifurcated hydrogen bond between isocyanide **14** (CAGDEX, CCDC 2031759) and the adjacent polarized difluoromethylene H and the *o*-phenyl group. (C) The structure of **15** (FEZZAP, CCDC 2213853) exhibits two symmetric contacts between the isocyanide carbon and the aromatic ring *o*-hydrogen, as well as a hydrogen bonding interaction between the isocyanide C and the polarized hydrogen at position 5 bound to a sp^2^ carbon of the five-membered oxadiazole. (D) The methylene group H in **16** (MURXIJ, CCDC 2002330) is polarized and interacts with the isocyanide C of an adjacent molecule over a short distance. (E) Carbene complex **17** (USINAM, CCDC 793073) forming a short hydrogen bond (C···HC).

Hydrogen bonds CH···X (X = N or O) are considered as weak and have been less reported, because the acidity of the CH bond is mostly very low compared to that of NH or OH hydrogens.^54,55^ Even more weak would be a CH···C hydrogen bond. Interestingly, with 296 examples, we found quite some crystallographic evidence that hydrogen bonds between the isocyanide C can be involved in hydrogen bonding with the less electronegative CH, comprising a very rare C···HC hydrogen bond. Figure 8 shows several examples of this interaction. More specifically, in the crystal structure of **14**, two distinct C···HC hydrogen bonds are present on the basis of polarized CH groups. Herein, we define polarized CH as a hydrogen bound to a carbon close to an electron-withdrawing group (e.g., nitrogen, oxygen, halogen, or fluorine) or incorporated into a (hetero)aromatic ring system.^56^ The aromatic naphthyl CH in the *meta* position to the isocyanide and *ortho* position to the electron-withdrawing difluoromethyl thiol substituent features a short contact of 2.8 Å to the neighboring isocyano group. The N–C–H angle of 110° implies the potential involvement of the sp^3^ hybrid orbital of the isocyanide, and coplanarity occurs. Interestingly, a second CH···CNR hydrogen bond can be interpreted, involving the strongly polarized CH of the difluoromethylene thiol group to the neighbor isocyano group exhibiting a distance of 2.6 Å and a more obtuse NCH angle of 150°. In isocyanide **15**, an interaction between the isocyano C and the *o*-CH group of the neighboring molecule and an interaction between the isocyano C and the hydrogen of the heterocycle five-membered 1-oxa-2,4-diazole ring can be observed.^57^ This interaction is assisted by the highly polarized nature of the CH group surrounded by the electronegative oxygen and nitrogens. The isocyanide 1,3-diisocyano-2,2-bis(isocyanomethyl)propane **16** is one of the very few known tetraisocyanides and features *S*_4_ symmetry in solution.^58^ The well-described electronegativity of the isocyano group renders the α-mehylene CH group acidic, and the polarized CH group undergoes a hydrogen bond with the neighboring isocyano group of 2.6 Å and an angle (NCH) of 147°. Carbenes are isoelectronic to isocyandes, and they are considered to be in oxidation state C^II^. Interestingly, there are CSD structures of carbene complexes described in which the carbene C also acts as a hydrogen bond acceptor, comprising another example of the almost elusive CH···C hydrogen bond. In imidazole-derived carbene **17**, a short C···HC distance of 2.2 Å and an angle (CHC) of 172° can be observed.^59^

### Multipolar RNC···(HX)_*n*_ Interactions

**Figure 9:**
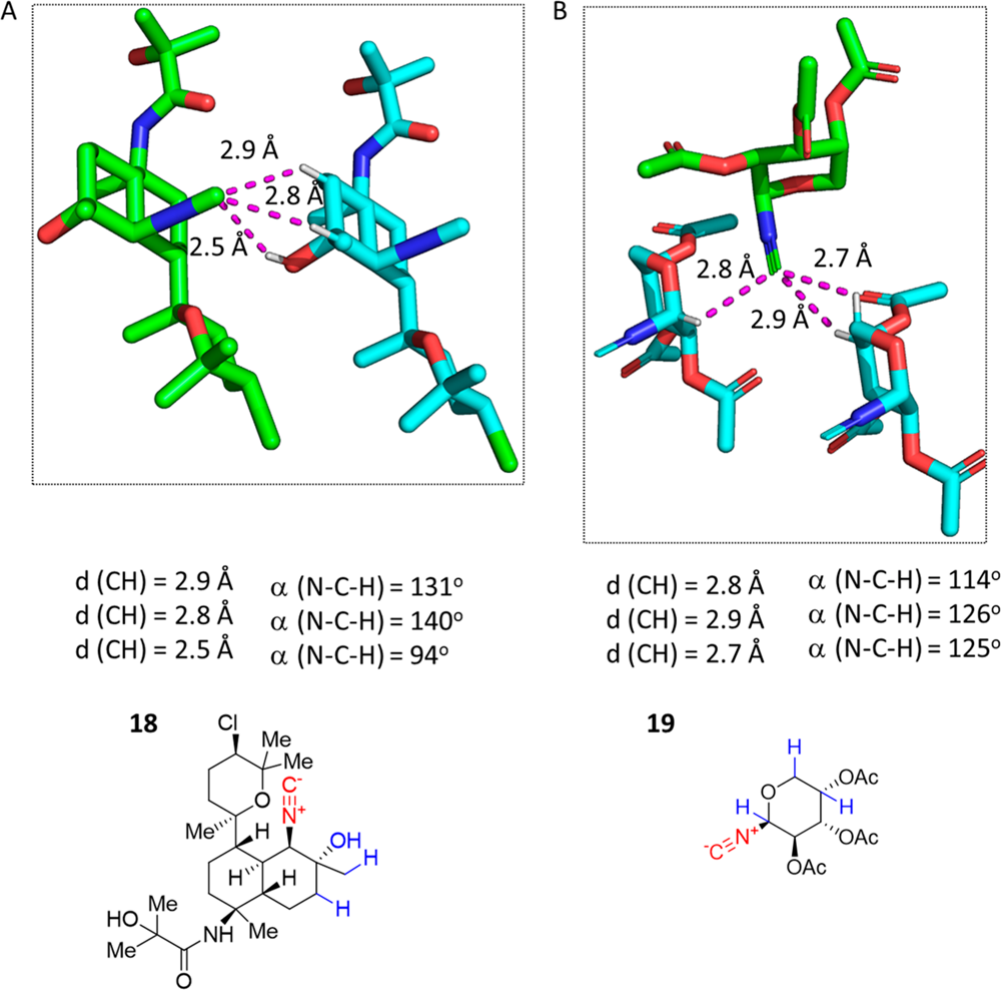
Examples of multipolar hydrogen bonding interactions RNC···(HX)_*n*_. (A) Isocyanide **18** (JEVSEM, CCDC 2155533) features interactions with three separate hydrogens at close range, in an approximate sp^3^ orientation. The contact also happens for hydrogens linked to inactivated carbon atoms. (B) In glycosyl isocyanide **19** (RIXDEK, CCDC 1869943), polarized CH groups from two neighbors sit close to the isocyanide C.

In a hybridization model, the isocyanide C orbital can be described in an approximately sp^3^-type orientation that may support multipolar hydrogen bonding interaction with up to three hydrogen partners. In our search, we spotted eight examples in which the isocyanide C orbital exhibits three interactions. Cytotoxic kalihinene diterpene **18**, isolated from the marine sponge *A. cavernosa*, depicts such a case (Figure 9A).^60^ The isocyanide C interacts with three different hydrogens of a neighboring molecule at short distances, which can be described as a sp^3^-type interaction. This involves a hydrogen bond with OH at 2.5 Å and two interactions with unpolarized aliphatic CH groups at 2.8 and 2.9 Å. Another multipolar hydrogen bonding interaction occurs in β-arabinose-derived peracylated isocyanide **19**.^61^ The isocyanide C undergoes three interactions with polarized CH groups at distances between 2.7 and 2.9 Å.

### RNC···X Interactions (X = O, N, or C)

In addition to close contacts that can be interpretated as hydrogen bonding, we also encountered 67 short RNC···O, RNC···N, and RNC···C contacts, shorter than the sum of the van der Waals radii, implying an attractive interaction. Many of the close contacts between the RNC and X are mediated by hydrogen bonds, but few show no hydrogen.

### Isocyanide O Interactions (RNC···O)

**Figure 10:**
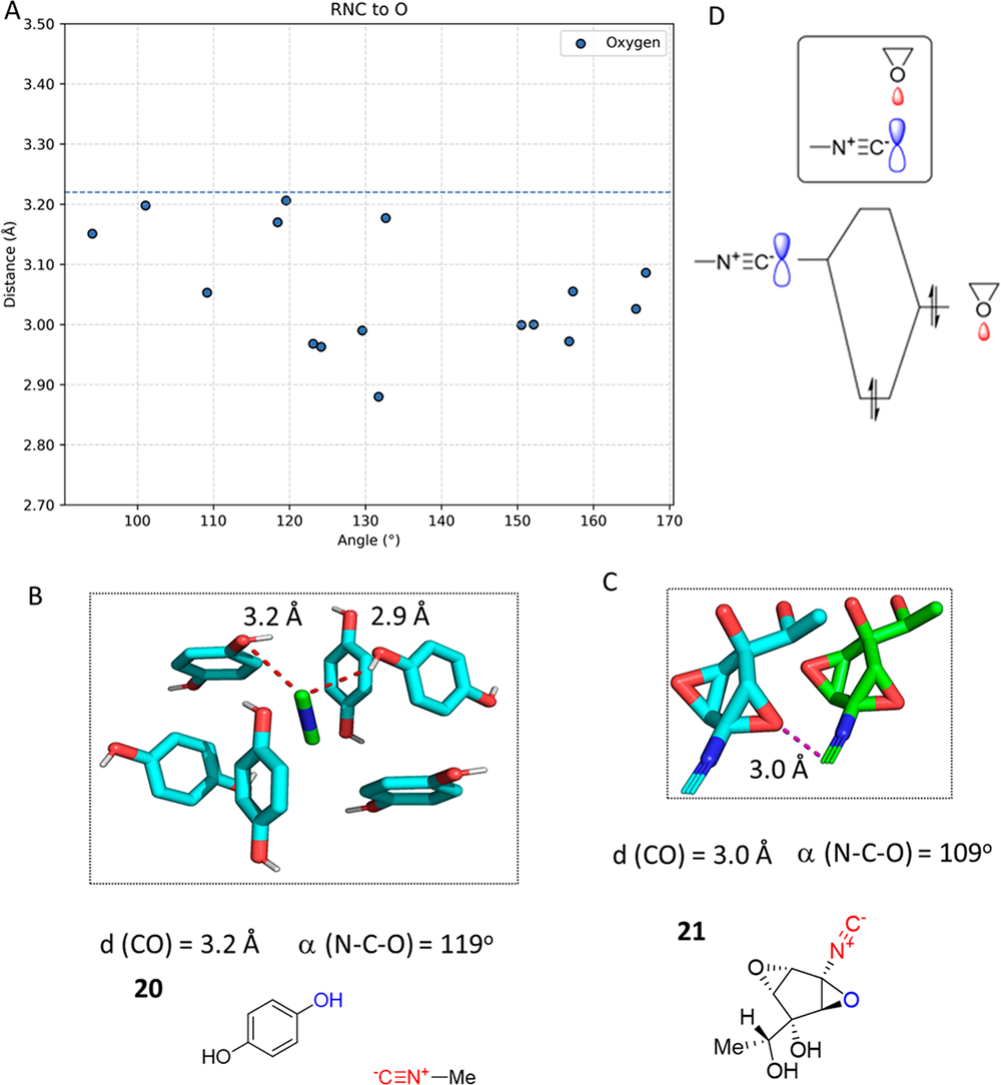
Examples of RNC···O interactions in the CSD. (A) Scatter plot of the interactions between the carbon of the isocyanide and oxygen atoms. The *y*-axis represents the interaction distances, and the *x*-axis represents the interaction angle. (B) In the cocrystal structure of **20** (BUSPAG, CCDC 1117233), the methylisocyanide forms a hydrogen bond to the hydroquinone’s hydroxyl group, and the oxygen atom interacts with the isocyanide over a short distance, 3.2 Å. (C) Trichoviridine **21** (TRIVIR01, CCDC 1275717) forms a short isocyano C–epoxide O contact of 3 Å. (D) FO interaction diagram of **21** isocyano C and epoxide O supporting the electrophilic character of isocyanides.

A total of 16 structures with short RNC···O contacts were found in the CSD (Figure 10A). *p*-Hydrochinone forms a clathrate with methylisocyanide **20** (Figure 10B).^62^ In this structure, the methylisocyanide is surrounded by six hydrochinone molecules. The isocyanide forms a hydrogen bond with the hydrogen of the hydroxyl group at 2.9 Å, and the hydroxyl oxygen atom interacts with the isocyanide at a short distance, 3.2 Å.

Another interesting case of short RNC···O interaction is seen in the solid state of antibiotic trichoviridine **21** (Figure 10C).^63^ Trichoviridine was isolated from a soil-borne fungus *Trichoderma* sp. and has been widely used for “biological” crop pathogen control. The structure comprises a highly unusual cyclopentane isocyanide diepoxide with a very low molecular weight, 183 Da. The isocyano C sits 3.1 Å close to the epoxy O of a neighboring molecule. The unusual interaction can be described as the filled HOMO O σ orbital interacting with the LUMO π* orbital with the highest orbital coefficient on isocyanide C (Figure 10D), supporting the ambivalent electrophilic character of the isocyanide.

### Isocyanide N Interactions (RNC···N)

**Figure 11:**
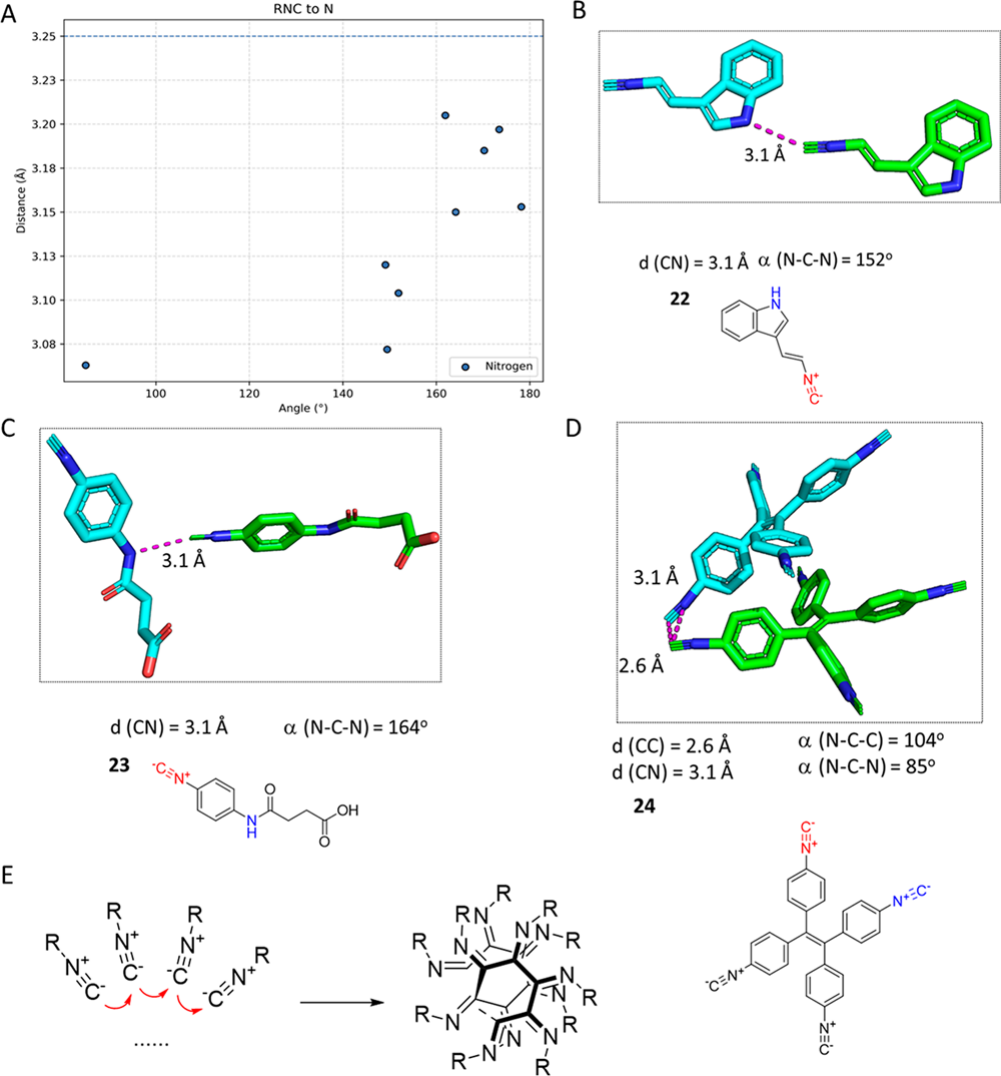
Examples of RNC···N interactions. (A) Scatter plot of the interactions between the carbon of the isocyanide and nitrogen atoms. The *y*-axis represents the distances of the interactions, and the *x*-axis the angle of the interaction. Nine different interactions were identified in this category. (B) α,β-Unsaturated indole isocyanide **22** (TAYGUW, CCDC 273844) forms a hydrogen bond-mediated short RNC···N contact. (C) *p*-Succinamide phenylisocyanide **23** (XAZQEW, CCDC 889762) forms another hydrogen bond-mediated RNC···N contact (3.1 Å), which is less than the sum of the van der Waals radius. (D) In tetraisocyanophenylethylene **24** (HEBNIP, CCDC 2112911), hydrogen bond donors are absent. Nevertheless, two isocyanide functional groups of two adjacent molecules of **24** form short isocyanide C···isocyanide C and isocyanide C···isocyanide N contacts. (E) Schematic presentation of the isocyanide polymerization.

We also found nine structures of isocyanides close to N, shorter than the sum of the van der Waals radii (Figure 11A). As in RNC···O, most interactions are mediated by hydrogen bonds (RNC···HN). In cases of isocyanides **22**(64) and **23**,^65^ there is clearly also hydrogen bond formation between the carbon of the isocyanide and the N group (Figure 11B,C). Thus, it is not clear whether these interactions occur because of the formation of hydrogen bonds or because of the ambivalent behavior of the isocyanide moiety, as a nucleophile and as an electrophile, or as a combination of both effects. Interestingly, in the case of isocyanide **24**,^66^ the 3.1 Å N···C interaction occurs without any hydrogen bonding involved (Figure 11D). The carbons of the neighboring isocyanides interact with each other over a short distance, 2.6 Å, which is the shortest interaction between carbon atoms that we observed. It is well established that some isocyanides have a tendency to spontaneously polymerize or polymerize with the support of catalysis to stable α-helical polyisocyanides.^67^ This short C–C distance can be interpreted as the first committed step of isocyanide polymerization (Figure 11E). It is noteworthy that tetraisocyanophenylethylene **24** exhibits interesting aggregation-induced emission properties in the solution state and mechanochromic behavior in the solid state.^66^

### sp^2^-C=X Isocyanide Interaction (RNC···C=X)

**Figure 12:**
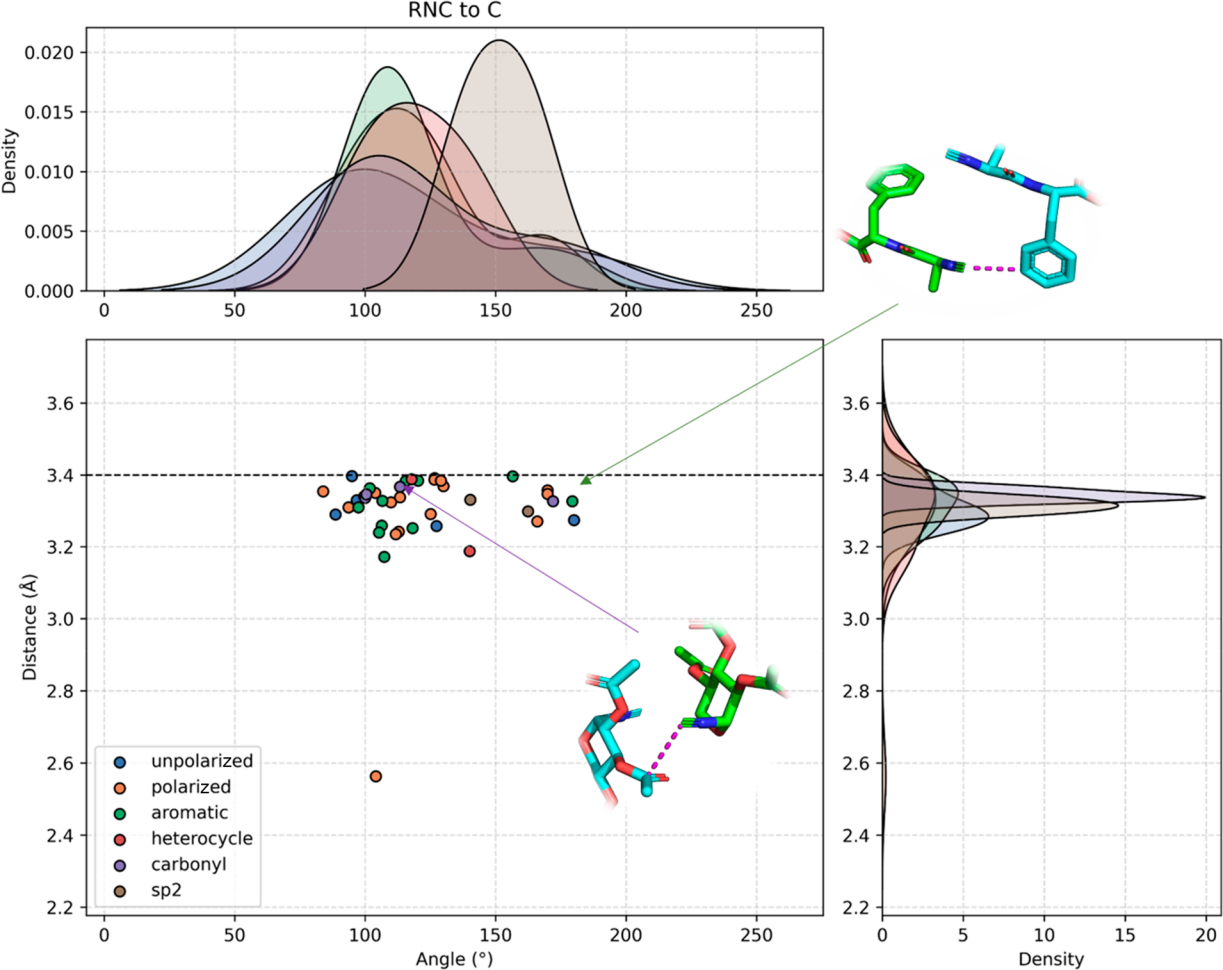
Examples of C···CNR interactions. Different categories are shown involving the interaction between the isocyanide C and carbons that are unpolarized, polarized, aromatic, heterocyclic, carbonyl, or sp^2^-hybridized.

Among the 42 CSD-spotted RNC···C interactions (Figure 12), the most interesting involve sp^2^-C binding partners. Intermolecular interactions with π systems are often observed in biochemical interactions, e.g., π stacking interactions between aromatic amino acids or between an aromatic moiety of a ligand in the aromatic receptor pocket. However, π interactions are not restricted to biochemistry and medicinal chemistry but play an equally important role in materials. Isocyanides can interact with the π orbitals of the partner. Several types of isocyanide π interactions can be observed in the solid state.

### Isocyanide C Carbonyl/Imine C Interaction (RNC···C=O/RNC···C=N)

**Figure 13:**
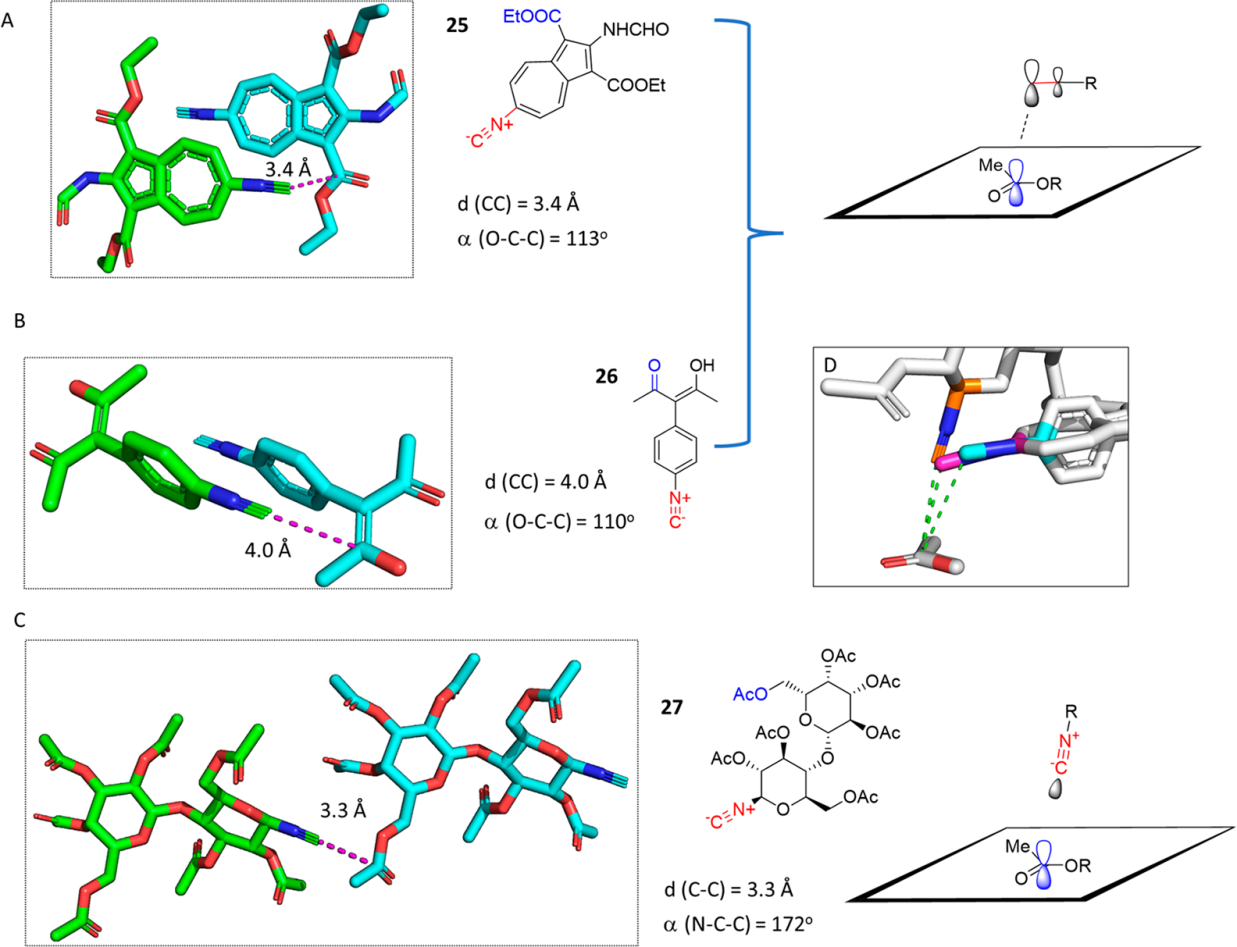
Crystallographic evidence of the Bürgi–Dunitz trajectory in nucleophilic attack of isocyanide C on nearby carbonyl C atoms. Isocyanides (A) **25** (XEDRII, CCDC 601010) and (B) **26** (DUZXUS, CCDC 790503) approach the carbonyl plane coplanar, suggesting the involvement of primarily isocyanide C σ and π orbital contributions. (C) **27** (RIXDUA, CCDC 1869946) approaches orthogonal to the carbonyl plane. The crystal structures of β-lactosyl isocyanide and azulene isocyanide (XEDRI, CCDC 601010) are shown. (D) Alignment of the carbonyl part (white–red sticks) of the three structures to underscore the Bürgi–Dunitz trajectory. The isocyano part and the first adjacent atom are shown as pink (**25**), cyan (**26**), and gold (**27**) sticks. The remaining molecules are shown as white sticks.

The Bürgi–Dunitz angle is a popular way to describe the geometry of an attack of a nucleophile on a trigonal unsaturated center in a molecule, initially the carbonyl center in an organic ketone but later extended to aldehyde, ester, and amide carbonyls as well as alkenes. Honoring its discoverers, the crystallographers Hans-Beat Bürgi and Jack D. Dunitz, it is called the Bürgi–Dunitz trajectory.^68−70^ Crystallographic analysis revealed the optimal angle of attack as 105°. Recent quantum mechanical calculations attributed the origin of the favored trajectory to several factors, including electrostatic interactions, Pauli repulsion between the nucleophile HOMO and ketone π(C=O), and HOMO (nucleophile) π*(C=O) LUMO orbital interactions.^71^ The observation of the interactions of isocyanide C with carbonyl C and imine C is particularly significant for the understanding of the mechanism of isocyanide-based multicomponent reactions such as the Ugi and Passerini MCRs. In a number of crystal structures, the isocyanide C approaches a nearby carbonyl C, which can be interpreted as representatives of the Bürgi–Dunitz trajectory (Figure 13A–C). The Bürgi–Dunitz trajectory traces point along the pathway of bond formation between a nucleophile and an electrophile.^68,72^ In a peracylated lactose derivative, β-lactosyl isocyanide **27**, the isocyanide C approaches the acetyl C within 3.3 Å, nearly perpendicular to the plane of the acetyl group with an angle (RNC···C=O) of 102°.^73^ Another example of a Bürgi–Dunitz approach of an isocyanide C to a carbonyl can be observed in the structure of substituted azulene isocyanide **25** with a distance (RNC···C=O) of 3.4 Å.^74^ Here, the isocyanide approached the carbonyl C in a coplanar fashion, indicating major π orbital contributions. A third case involves the 3-(4-isocyanophenyl)-2,4-pentanedione C approaching acetylacetone **26** at position 4 at a longer distance of 4 Å.^75^ The isocyano group is again coplanar with the C=O group, suggesting the possible involvement of the isocyanide π-HOMO–1.

**Scheme 1:**
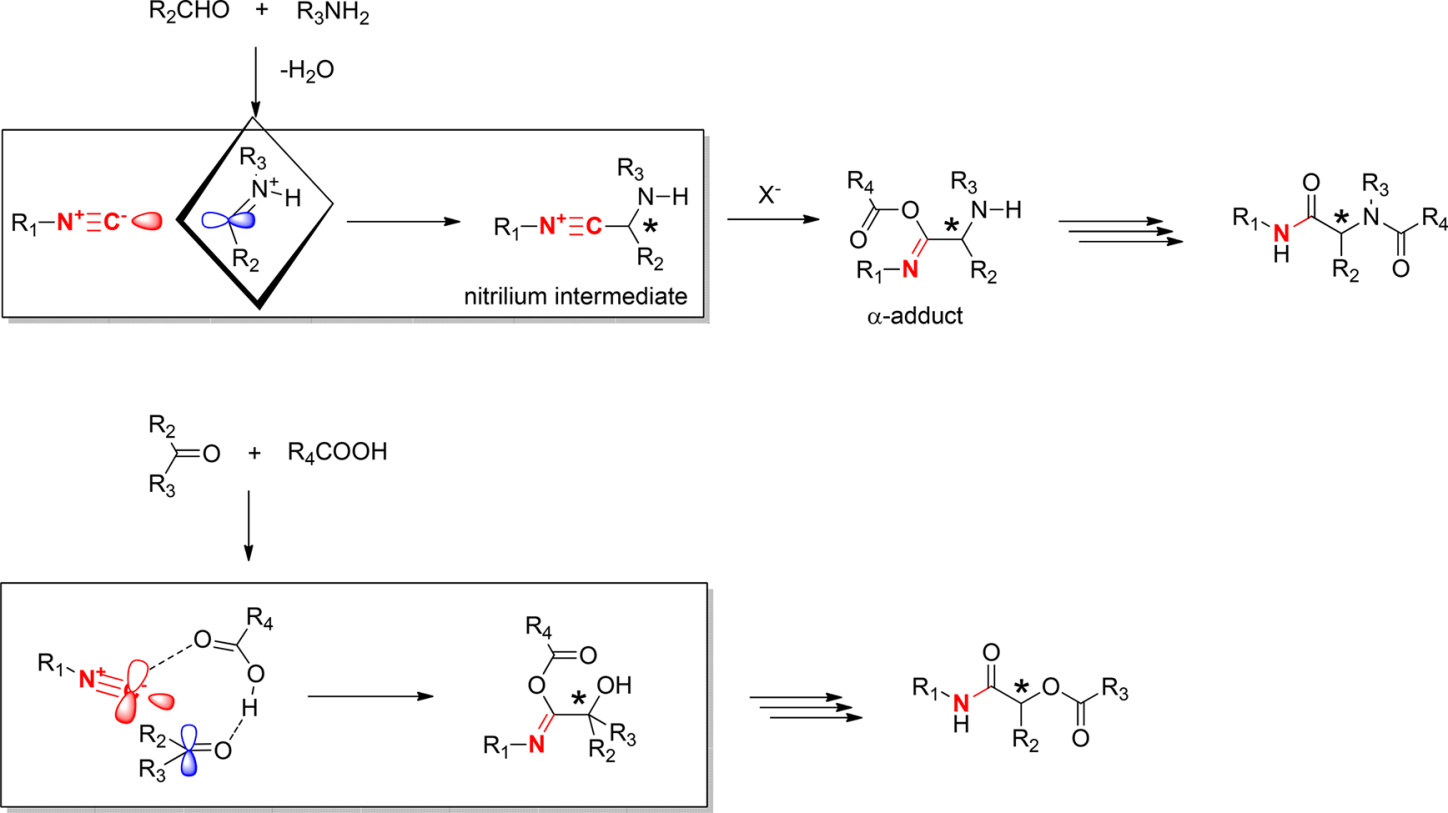
Key Steps of the Ugi and Passerini MCRs Involving the Nucleophilic Attack of the Isocyanide C on the Imine and Carbonyl C, respectively

The discovery of these X-ray structures is helpful in understanding the mechanism of the famous isocyanide-based Ugi and Passerini reactions. Both Passerini and Ugi reactions have a nucleophilic attack of the isocyanide C on a sp^2^-C, the carbonyl C (Passerini) or the imine C (Ugi) in common. In fact, in both reactions, it is the only stereochemistry-determining step (Scheme 1).

In the Ugi reaction, the nucleophilic isocyanide is believed to attack the imine C, to form the nitrilium ion intermediate, which also can be observed as an intermediate by mass spectrometry (Scheme 1). It is plausible that the nucleophilic attack occurs through the C-centered HOMO σ orbital and the LUMO π orbital on the imine C, reminiscent of a classical Bürgi–Dunitz trajectory. This step can be catalyzed by a Lewis or Bronsted acid, by increasing the electrophilicity of the imine through addition to the imine N. The prochiral imine is converted into the chiral nitrilium ion, which upon nucleophile addition to the nitrilium C and subsequent rearrangement irreversibly yields the final Ugi product. In the case of a carboxylic acid nucleophile, the product is the well-known α-amino acylamide. Today, chiral catalysts are available, which can reliably induce the new stereocenter with a very large enantiomeric excess.^76^ While the Ugi reaction is conducted in a polar protic solvent, the Passerini reaction requires an apolar aprotic environment. The oxo component is activated by the carboxylic acid to form a hydrogen bond-mediated noncovalent molecular pair. The isocyanide attacks the activated carbonyl C to form a nitrilium ion, the stereochemistry-determining step. Upon addition of the carboxylate to the nitrilium C forming the α adduct and further transacylation, the Passerini product α-hydroxy acylamide is formed. Due to the aprotic environment of the Passerini reaction, chiral catalysts could be developed much earlier than for the protic Ugi reaction.^77^ The crystallographic experimental evidence described herein for the first time for nucleophilic isocyanide C attack on carbonyl C atoms corroborates the evidence for the mechanism of several isocyanide-based MCRs and can stimulate further theoretical and crystallographic studies.

### RNC···Aromatic Interaction and RNC···C=C Interaction

**Figure 14:**
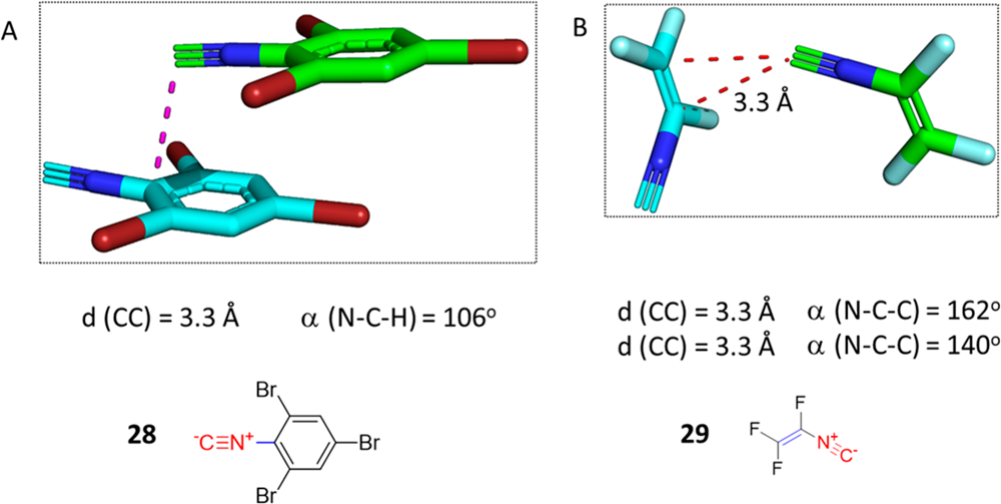
Examples of RNC···sp^2^-C interactions. (A) 2,4,6-Tribromophenyl isocyanide **28** (TBZINT01, CCDC 1445499) exhibits π–π stacking interaction between the isocyanide C and the ipso aromatic carbon. (B) Trifluorovinyl isocyanide **29** (MELFOY, CCDC 147927) interacts with both sp^2^ vinyl carbons at the same distance (3.3 Å).

Continuing our search, we found crystallographic evidence in nine examples that isocyanide C atoms can interact with aromatic and alkene carbons through π–π stacking interactions. The interaction involves the π orbitals of the carbon of isocyanide and π orbitals of the aromatic carbon. A representative example is phenyl isocyanide **28** that is sitting on top of the ipso-C of an adjacent parallel phenyl isocyanide molecule with a short distance of 3.3 Å (Figure 14A).^78^ In trifluorovinyl isocyanide **29**, which was measured by low-temperature X-ray crystallography, the isocyano C exhibits a T-shaped conformation on top of the π cloud of the vinyl group of an adjacent molecule at the same distance to both sp^2^ carbons in the sp^2^ system (Figure 14B).^79^

### Isocyanide–Halogen Interaction

**Figure 15:**
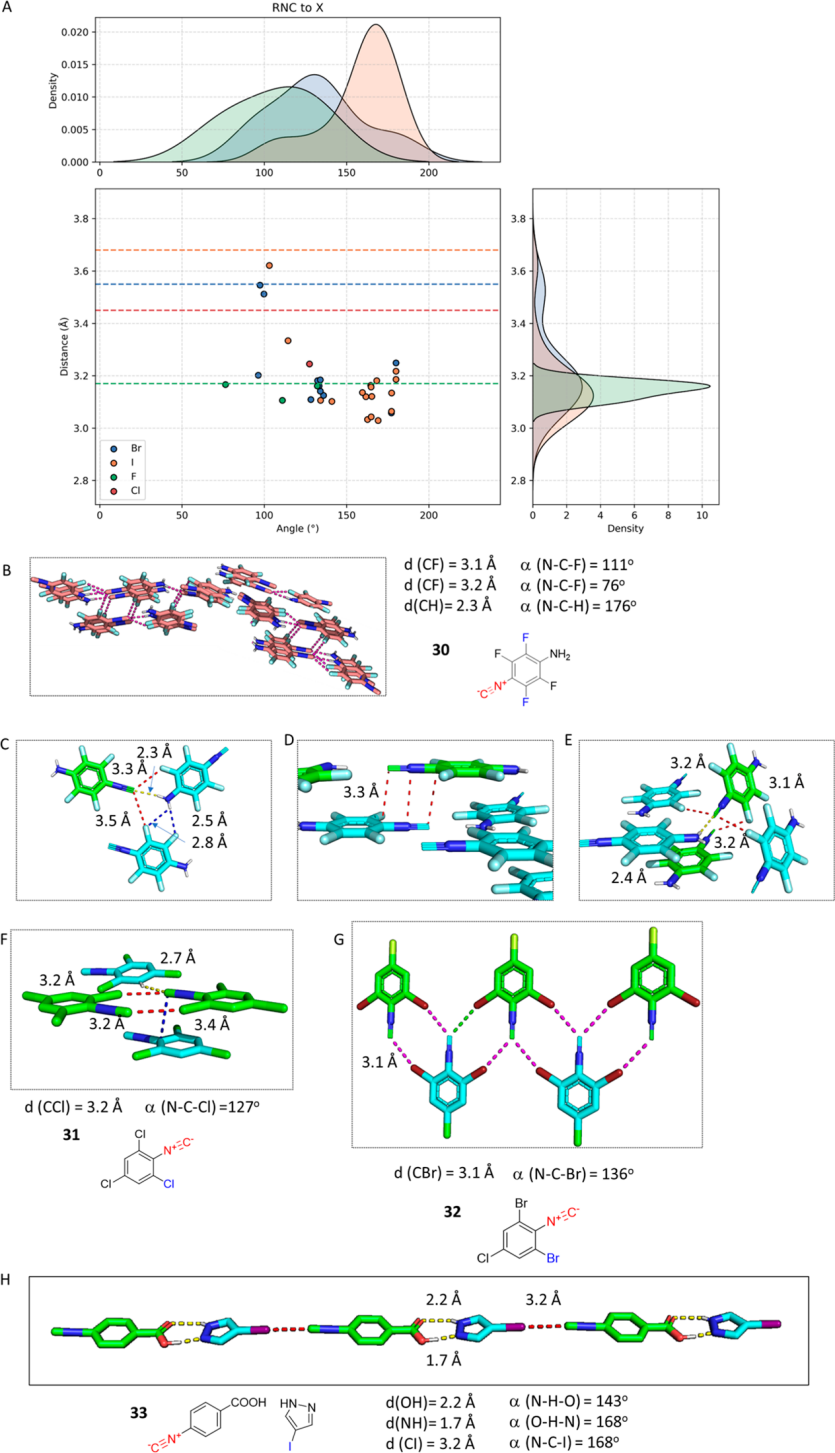
Examples of RNC···X halogen bonding. (A) Scatter plot of all of the halogen bonds, including the van der Waals radius isobars of the halogen C indicated as dotted lines. (B) Fish bone arrangement of a layer of 4-isocyano-2,3,5,6-tetrafluoro aniline **30** (FOGFUD, CCDC 253149). (C) Multipolar interaction hydrogen bonding-mediated motif including three RNC···X contacts and a bifurcated NH···F contact. (D) Molecules **30** in one layer are arranged coplanar and parallel to the adjacent layer, at a short average distance of 3.3 Å. (E) Multipolar interaction hydrogen bonding-mediated motif of **30** at the interface of two fish bones, including two RNC···F bonds and one RNC···HN hydrogen bond. (F) 2,4,6-Trichlorophenylisocyanide **31** (FUGVAE, CCDC 152633) assembles also in a parallel layer with an interlayer distance of 3.4 Å. Two neighboring isocyanides exhibit two RNC···Cl bonds (3.2 Å), resulting in dimers of **32** and also featuring a RNC···H bond of 2.7 Å. (G) Layered 2,6-dibromo-4-chlorophenylisocyanide **32** (MESRAG, CCDC 1812522) exhibits infinite coplanar antiparallel arrangements with bifurcated RNC···(Br)_2_ bondings of 3.1 Å. (H) The cocrystal of 4-isocyano benzoic acid with 4-iodo pyrazole **33** (PEKWIN, CCDC 898814) features a short quasi-linear RNC···I distance. The incorporation of an intermolecular bifurcated hydrogen bond between carboxylic acid -COOH and pyrazole NNH leads to an infinite chain arrangement of **33** in the crystal.

Halogen bonding (HB) comprises an important albeit often weak interaction and has found extensive applications in medicinal chemistry and materials science.^80−82^ While the detailed quantum mechanical nature is still under debate, a simple definition of HB is the interaction of a nucleophile with the electron hole on the tip of the σ orbital of the heavier halogens.^82−84^ Advanced electron microscopy techniques, including kelvin probe force microscopy, successfully visualized the anisotropic charge distribution of the σ hole.^85^ HB is a beneficial addition to the set of advantageous interactions in molecular recognition and, in some circumstances, can result in large increases in affinity. According to statistical analysis of crystallographic data and quantum mechanical calculations, nucleophiles approach the halogens “head on” while electrophiles preferentially create “side-on” interactions with them.^82^ Recent investigations also point to a considerable polarization impact dependence in the halogen’s local environment. This suggests that extra cooperative interactions in the binding region, in addition to tuning effects based on changes to the scaffold, may improve or weaken the halogen bond strength. The binding site should affect iodine more than bromine and bromine more than chlorine because the polarizability dramatically increases with atom size. Moreover, the electrostatic attraction between the electron-deficient regions of the halogen’s σ hole and the Lewis base interaction partner, here the carbon atom of the isocyanide, is a key factor driving halogen bonding, with polarization and dispersion effects also playing significant roles. Therefore, by the addition of electron-withdrawing substituents to a given scaffold, it is possible to adjust the strength of halogen bonds. In our query, we found 31 short X···CNR contacts, *ecce* halogen bonds, where X = fluorine, bromine, chlorine, or iodine, and some examples are discussed below (Figure 15). 2,3,5,6-Tetrafluoro-4-isocyano aniline **30** has an overall herringbone pattern in which layers of **30** are arranged in an alternating skipped fashion. **30** exhibits a remarkable wealth of shorter than van der Waals contacts between layers of coplanar molecules, but also interlayer π stacking contacts (Figure 15B).^86^ One intralayer motif contains three parallel molecules of **30** in which the isocyanide C is making three close contacts (Figure 15C), two RNC···F (3.3 and 3.5 Å) and one RNC···HN hydrogen bond (2.3 Å). The short interlayer distance between adjacent layers of molecules of **30** of 3.3 Å is noteworthy, pointing to extensive π stacking interactions (Figure 15D). Interestingly, there is an antiparallel orientation of **30** in two adjacent layers with an attractive dipole momentum orientation (Figure 15D). The isocyano groups of **30** in the adjacent layer are arranged on top of each other in an antiparallel fashion and with a short isocyano N···N contact of 3.3 Å. Another binding hot spot is at the interface of two fish bones (Figure 15E). The isocyanide C is forming two short F contacts (3.2 and 3.1 Å), one in the same bone and one to a molecule in the adjacent bone, mediated by a short hydrogen bond in **30**-NH_2_ (2.4 Å). 2,4,6-Trichlorophenyl isocyanide **31** shows a symmetrical bifurcated HB between two neighboring molecules, involving a short isocyanide C···Cl contact of 3.2 Å and an angle (NCCl) of 127° (Figure 15F).^87^ Isomorphic 2,6-dibromo-4-chlorophenyl isocyanide **32**, however, undergoes a bifurcated HB network of similar distance and angle as **31** of adjacent coplanar molecules leading to a 1D indefinite assembly (Figure 15G).^88^ Cocrystal **33** of *p*-isocyanobenzoic acid and 4-iodopyrazole is a nice example of a crystal engineering approach, involving the head-to-head pyrazole–carboxylic acid synthon and another head-to-head I···CNR bonding for the construction of cocrystals and extended architectures in organic solids (Figure 15H).^89^

## Summary

**Figure 16:**
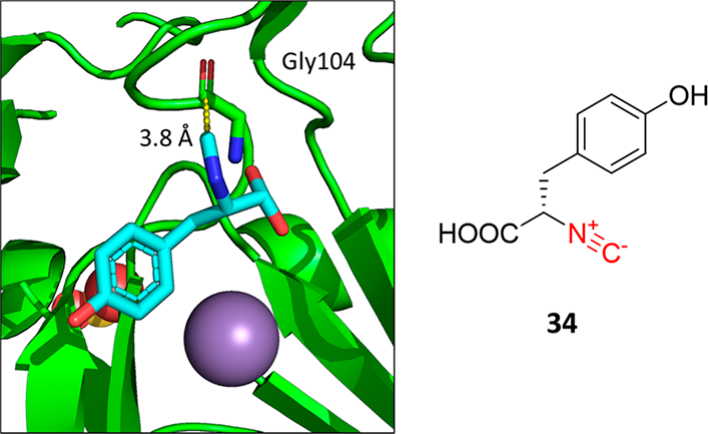
Isocyanide protein cocrystal structure (PDB entry 7TCL). The protein secondary structure is shown as a green cartoon, and Tyr isocyanide **34** as cyan sticks. The manganese atom and sulfate ion are represented as gray and red/yellow spheres, respectively. The isocyano C forms a contact to the Gly104 amide carbonyl C at 3.8 Å (yellow dotted lines).

The isocyanide, despite its small size and diatomic nature, displays an exceptionally rich structural chemistry. By analyzing the CSD, we found examples of the isocyanide involved in polar hydrogen bonds, including -NH and aromatic and aliphatic -OH. Moreover, hydrogen bonds to polarized CH groups are quite common. Multipolar hydrogen bonds involving one isocyanide C bond and up to three surrounding hydrogens were observed. Surprisingly, isocyanide C–carbonyl C interactions were found, suggesting a Bürgi–Dunitz trajectory, which can help to explain the mechanism of the Ugi and Passerini reactions. Interactions between isocyanide C and aromatic or isolated π electron systems are common. Close contacts between isocyanide C and fluorine and the heavier halogens are common. Notably, in almost all interactions, only the isocyanide C is involved and not the N, based on the distance analysis. This and the rich structural chemistry can be rationalized in the framework of the frontier orbital theory of this amphiphilic functional group. The key frontier orbitals are a C-centered HOMO σ orbital, a C-centered HOMO–1 π orbital, and a C-centered LUMO π orbital. The all-C-centered orbital distribution accounts for the nucleophilic and electrophilic character, the Lewis acid and base, the hydrogen bond acceptor behavior, and *ecce* the chameleonic behavior of the isocyanide. We also analyzed carbenes that are isoelectronic to isocyanides and were found to undergo similar intermolecular interactions in the CSD. We and others believe that isocyanides have a bright future not only in synthetic chemistry and the application of materials but also in medicine.^30^ Currently, the structural biology of isocyanides is mostly restricted to heme–Fe interactions, and only one isocyanide cocrystallized with the protein undergoing a different interaction is known (Figure 16).^90^ In this structure, Tyr isocyanide **34** is cocrystallized with an iron- and 2-oxoglutarate-dependent (Fe/2OG) enzyme that is part of the biosynthetic gene cluster involved in the biosynthesis of isocyanide-containing natural products. This isocyanide **34** is bound in the substrate pocket on top of the catalytic Mn^2+^ ion. Interestingly, the isocyano group is not involved in the metal complexation but rather features a 3.8 Å contact to the Gly104 backbone amide carbonyl C, approaching it orthogonally in the sense of a Bürgi–Dunitz trajectory.

Many known biological activities of isocyanides depend on their ability to coordinate metals, which accounts for the antibiotic activity of the drug xanthocillin **7**.^23^ From the perspective of a ligand receptor interaction, we predict that isocyanides can play an exquisite role as ligands in drug discovery beyond metal binding, being able to interact through polar hydrogen bonds with backbone amide groups as well as the side chains of Tyr, Ser, Thr, His, Tyr, Trp, Asn, and Gln. Moreover, stacking and isocyanide C interactions with the π cloud of aromatic amino acid side chains and the amide carbonyl C interactions can be expected. The prevalence of isocyanide intermolecular noncovalent interactions, along with evidence of their widespread occurrence in natural products, raises questions about the potential usefulness of this class of small molecules in targeting biomolecules, emphasizing a new opportunity to expand our current arsenal of functional groups in medicinal chemistry and include isocyanide in the design of drugs for unmet medical needs. Against the widespread prejudice that isocyanides are chemically and metabolically unstable, a recent investigation of the hepatic metabolism of six model isocyanides revealed that the stability indeed can be fine-tuned and secondary and tertiary isocyanides are metabolically stable.^91^

With regard to the method applied in this work, it has to be noted that careful interpretation is required when analyzing the results and drawing conclusions, as crystal packing effects can influence the assembly and orientation of molecules in relation to their neighbors. The analysis of molecular interactions in the CCDC relies on the statistical analysis of a large number of crystal structures. However, the number of non-metals coordinating isocyanides is currently relatively low. While the association of molecules in crystals does not establish the causation of pairwise functional group interactions, it is also important to consider the overall packing arrangement. Nevertheless, the observed intermolecular interactions in the solid state align with our current understanding of isocyanide reactivity, providing valuable insights into their potential interactions. We hope that our findings will stimulate further investigations, including the design and discovery of novel isocyanide reactivity, detailed quantum mechanical descriptions, systematic crystallographic studies, and analyses of cocrystal structures involving isocyanides in proteins, such as fragment screening.

## Data Availability

The data underlying this study are available in the published article and its Supporting Information.

## Supporting Information Available

A comprehensive list of all isocyanide complexes found in the CSD (June 2023.1) and data mining in crystal structure databases (PDF)
