# Supplementary material for: Crystal Clear: Decoding Isocyanide Intermolecular Interactions through Crystallography
Source: J Org Chem. 2024 Jan 4;89(2):957–74. doi: 10.1021/acs.joc.3c02038 (PMC10804414; doi:10.1021/acs.joc.3c02038)
Supplement: Supplementary file 1 — jo3c02038_si_001.pdf [file jo3c02038_si_001.pdf]

Supplementary Information

Crystal Clear: Decoding Isocyanide Intermolecular Interactions through Crystallography

Eleftheria Chatziorfanou,[1] Atilio Reyes Romero,[2,3,4] Lotfi Chouchane,[2,3,4] Alexander Dömling[1]*

[1] Institute of Molecular and Translational Medicine, Faculty of Medicine and Dentistry and Czech Advanced Technology and Research Institute, Palacky University in Olomouc, Olomouc, Czech Republic

[2] Genetic Intelligence Laboratory, Weill Cornell Medicine-Qatar, Qatar Foundation, Doha, P.O. Box 24144, Qatar

[3] Department of Microbiology and Immunology, Weill Cornell Medicine, New York, New York, 10021, USA.

[4] Department of Genetic Medicine, Weill Cornell Medicine, New York, New York, 10021, USA.

* email: E-mail: alexander.domling@upol.cz

Table of Contents

1. Molecular orbitals and FO's of MeNC……S2

2. Database……S3

   2.A) Isocyanides that form intermolecular interactions……S3

   2.B) Rest of organic isocyanides that do not form intermolecular interactions……S34

   2.C) Organometallic isocyanides forming interactions……S35

   2.D) Organometallic isocyanides not forming interactions……S38

3. Data mining in crystal structure databases……S39

4. References……S40

1. Molecular orbitals and FO's of MeNC

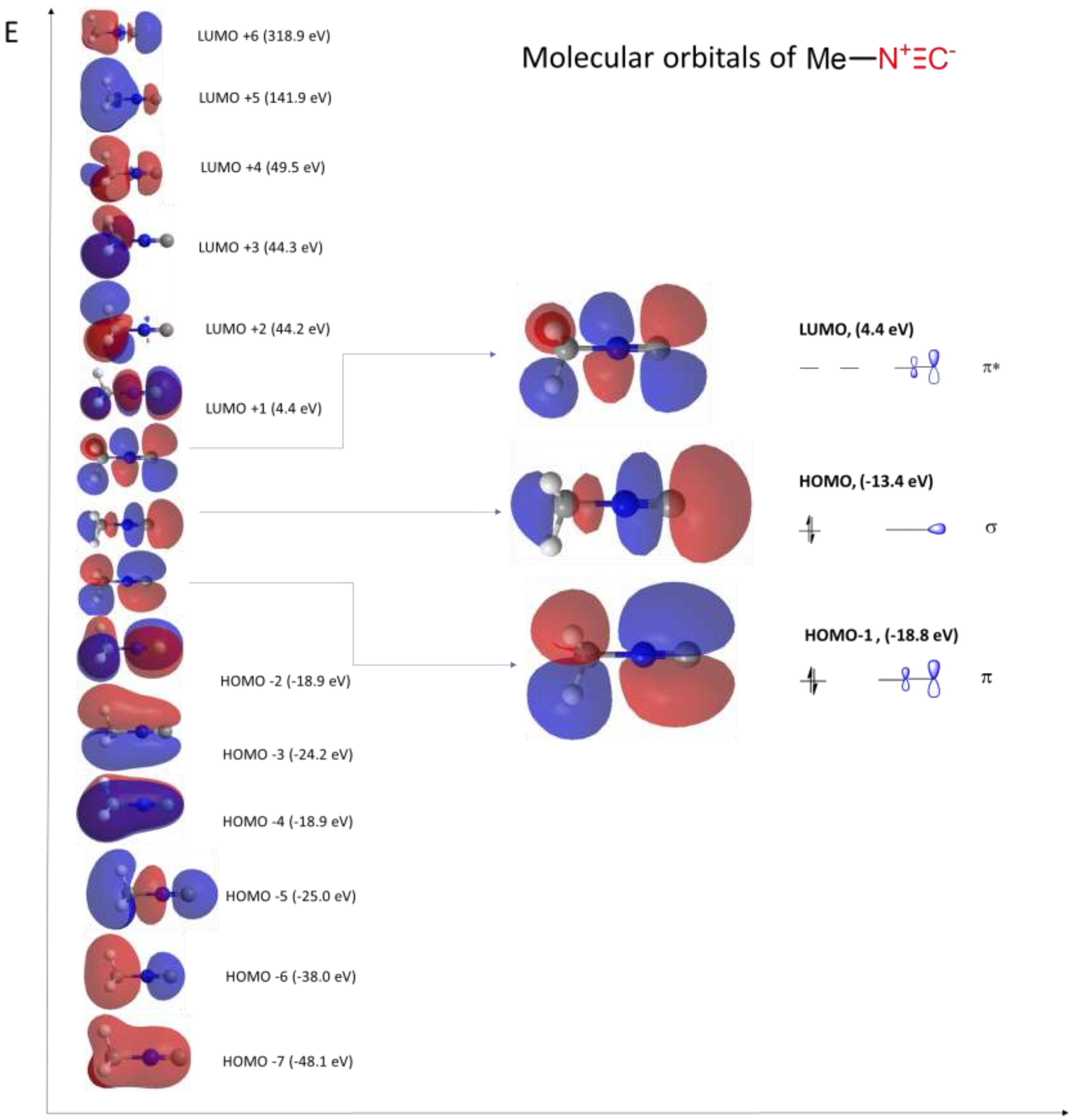

Figure S1. Molecular Orbitals of Methylisocyanide

2. Database

A) Isocyanides that form intermolecular interactions (van der Waal's radius ≤3.6 Å)

CCDC code	no	2D Structure	Distance (Å)	Angle (°)	Ref.	3D Structure
ACEZER<br>CCDC 2180959	1.	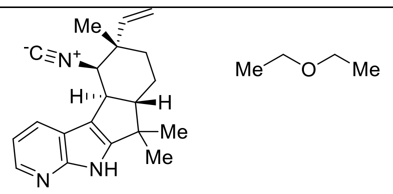	2.9	108.8	(1)	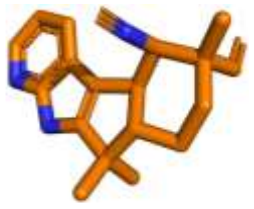
AHOGUA<br>CCDC 711704	2.	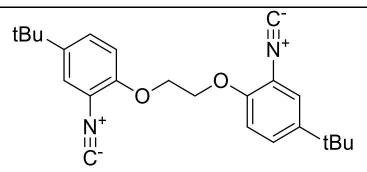	2.8<br>2.9<br>2.7	113.9<br>99.1<br>98.3	(2)	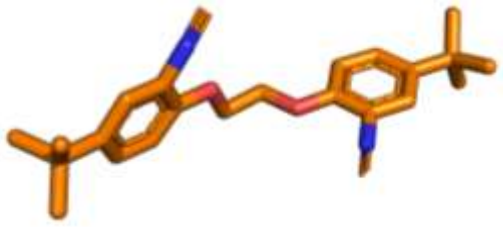
AKIFOP<br>CCDC 199143	3.	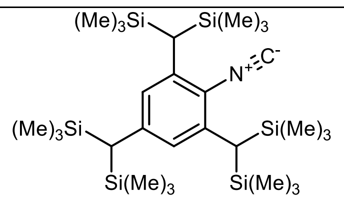	2.9	173.0	(3)	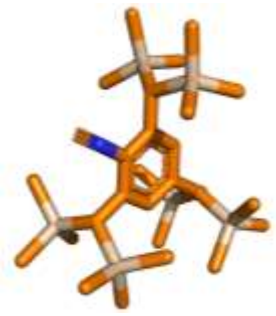
ASAKAI<br>CCDC 1454590	4.	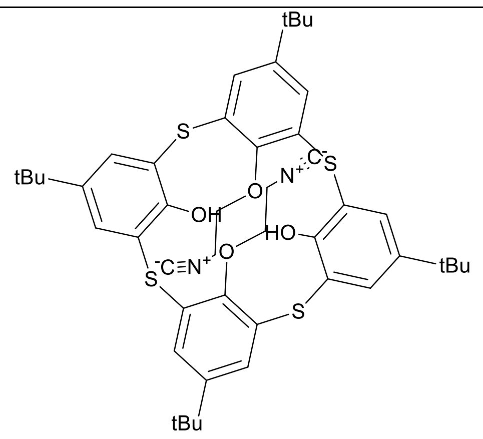	3.3<br>2.6<br>2.8<br>2.9<br>2.8<br>2.7	127.3<br>138.1<br>164.0<br>94.1<br>145.9<br>110.7	(4)	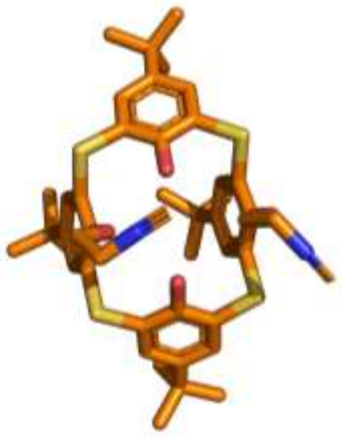

AXISNT<br>CCDC 1104008	5.	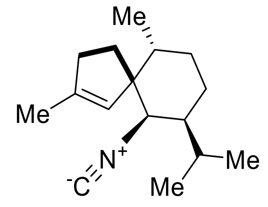	2.5	147.1	(55)	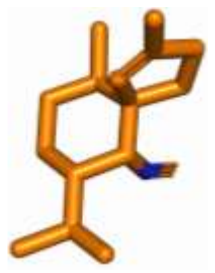
BAVHUB<br>CCDC 1106505	6.	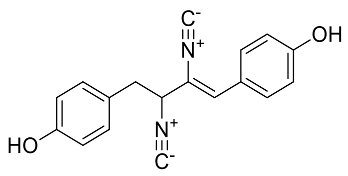	2.1<br>2.9<br>2.1<br>3.1<br>3.1<br>3.0<br>3.0	168.0<br>115.0<br>157.2<br>166.9<br>166.9<br>165.9<br>165.6	(6)	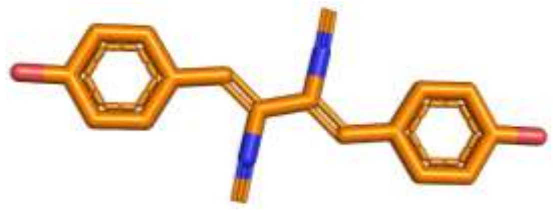
BBZICN<br>CCDC 1107148	7.	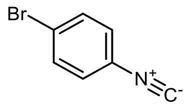	3.2	180	(7)	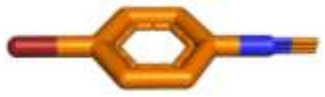
BECKUP<br>CCDC 1107627	8.	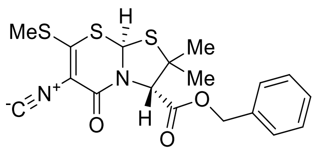	2.7<br>2.8	162.9<br>83.2	(8)	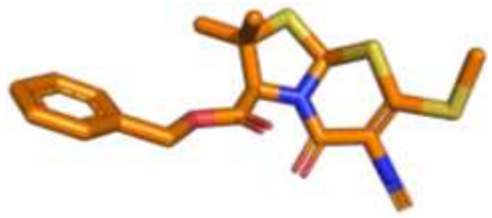
BIQJOD<br>CCDC 1870509	9.	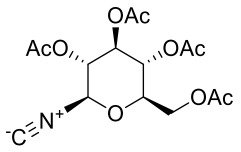	2.6	110.2	(9)	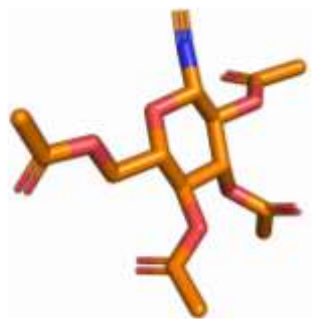
BIVFER<br>CCDC 1111836	10.	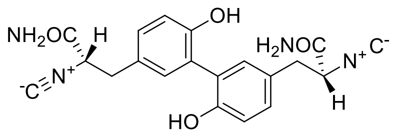	2.8<br>2.6<br>3.1<br>3.1	107.8<br>124.1<br>94.1<br>94.1	(10)	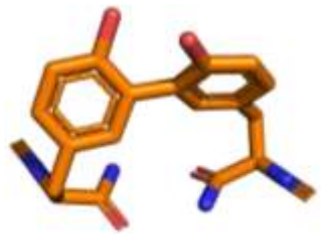

BOCQAO<br>CCDC 1855000	11.	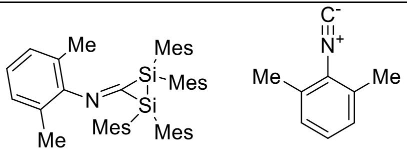<br>Mes= mesyti group	2.9	103.4	(11)	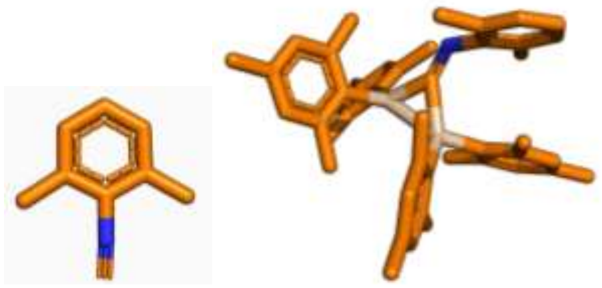
BUSPAG<br>CCDC 1117233	12.	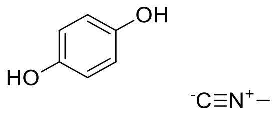	3.2<br>2.8<br>3.2	118.4<br>121.0<br>119.5	(12)	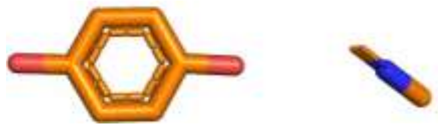
CAGDEX<br>CCDC 2031759	13.	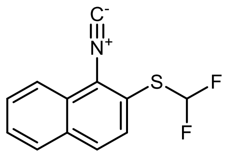	2.8<br>2.8<br>2.6<br>2.6	112.1<br>110.3<br>148.7<br>150.0	(13)	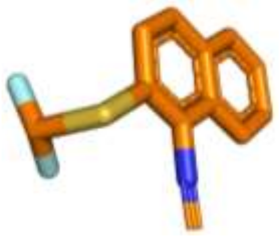
CAXTAZ<br>CCDC 1516436	14.	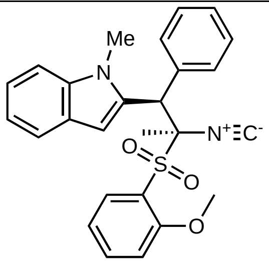	2.8<br>2.8	105.1<br>161.4	(14)	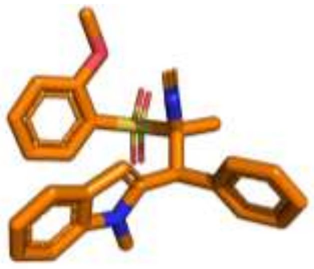
CESCIP<br>CCDC 1525814	15.	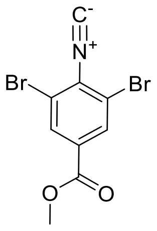	3.2<br>2.9<br>2.9	112.9<br>97.8<br>145.7	(15)	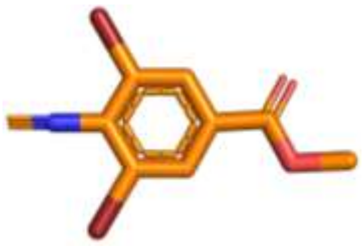
COGDUX<br>CCDC 1128672	16.	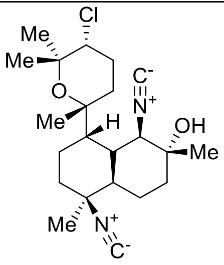	2.9<br>2.9<br>2.8<br>2.8	104.2<br>130.7<br>139.0<br>165.3	(16)	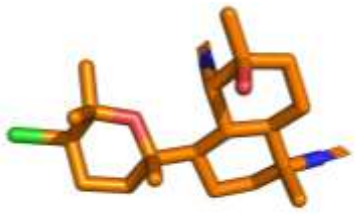
COXKOP	17.		2.8	122.6	(17)	

CCDC 1130516		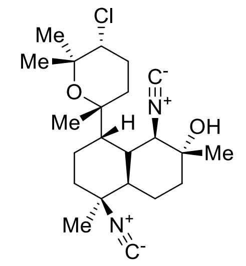	2.9<br>2.8<br>2.6<br>2.8<br>2.8<br>2.9<br>3.0<br>3.0<br>3.0<br>3.0	124.3<br>110.3<br>166.7<br>151.9<br>101.8<br>137.8<br>124.2<br>123.1<br>124,2<br>123.1		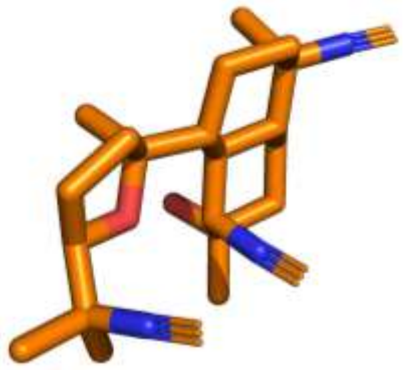
CUTREQ<br>CCDC 1405897	18.	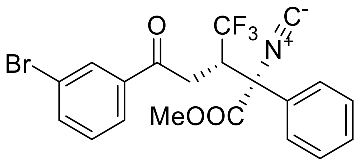	3.3<br>3.2<br>2.6<br>2.8	113.3<br>111.7<br>150.5<br>124.9	(18)	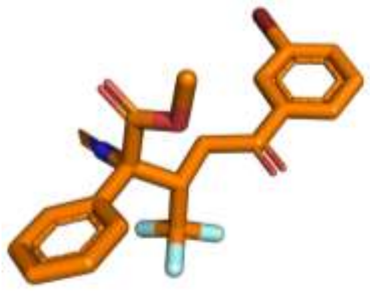
DALDEC<br>CCDC 1463862	19.	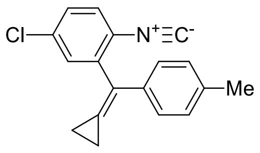	2.9<br>2.9	102.6<br>157.6	(19)	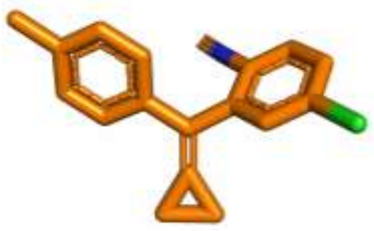
DICADC<br>CCDC 114026	20.	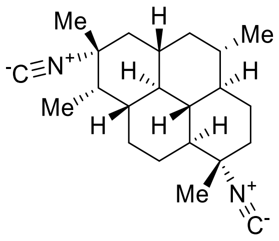	2.8<br>2.9	134.9<br>132.0	(20)	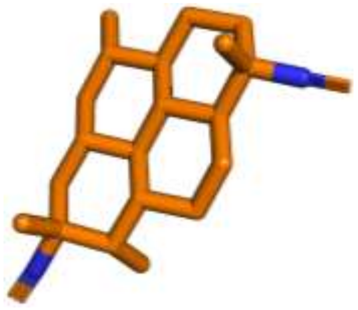
DUWGOR<br>CCDC 1147329	21.	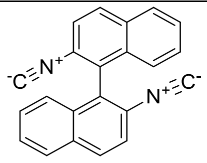	2.5<br>2.8	154.5<br>127.8	(21)	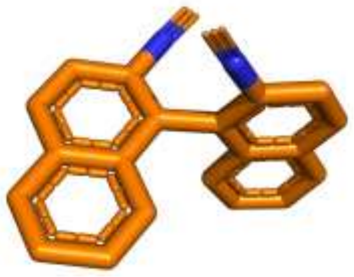

DUZXUS<br>CCDC790503	22.	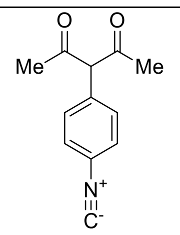	2.9	94.0	(22)	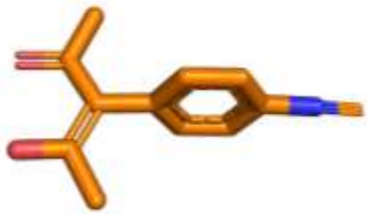
EBONAK<br>CCDC 155748	23.	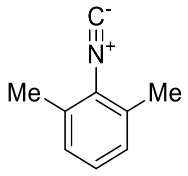	2.8<br>2.8<br>2.8<br>2.9	106.8<br>104.9<br>155.7<br>155.0	(23)	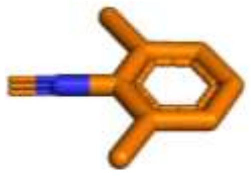
EBONAK01<br>CCDC 1938757	24.	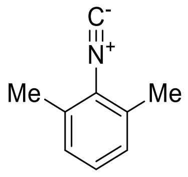	2.8<br>2.9	105.0<br>155.7	(24)	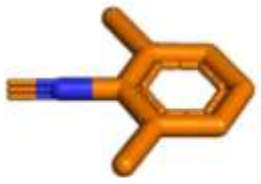
EVOSAJ<br>CCDC 243606	25.	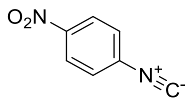	2.7<br>2.7	123.3<br>155.2	(25)	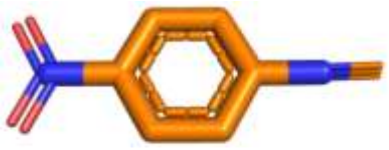
EVOSEN<br>CCDC 243607	26.	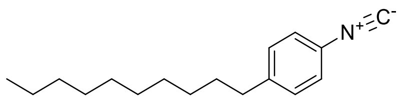	2,	128.5	(26)	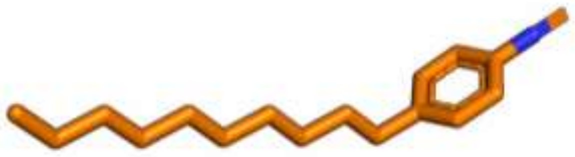
FEHBID<br>CCDC 1154077	27.	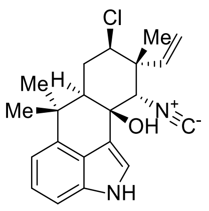	2.6	151.0	(27)	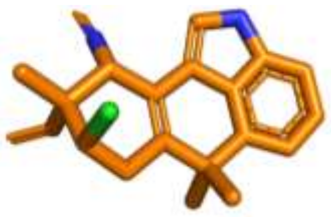
FEZZAP<br>CCDC 2213853	28.	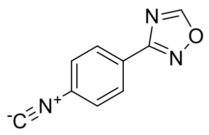	2.8<br>2.9<br>2.8	153.4<br>133.5<br>123.1	(28)	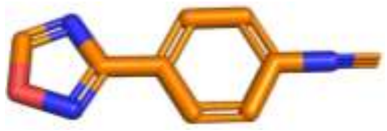

FIXXIT<br>CCDC 1157541	29.	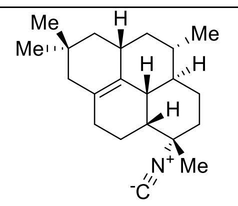	2.9	106.7	(29)	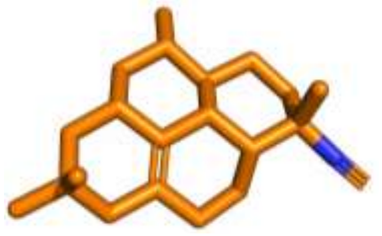
FIYDAS<br>CCDC 1157562	30.	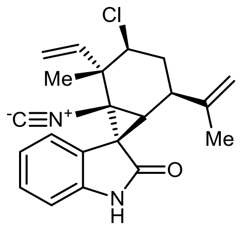	2.7	141.9	(30)	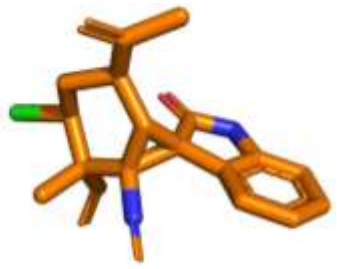
FOGFUD<br>CCDC 253149	31.	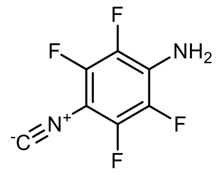	3.2<br>3.3<br>2.4<br>2.2<br>3.1<br>3.2<br>3.1<br>3.2<br>3.2	107.3<br>97.4<br>171,8<br>176.4<br>178.4<br>170.2<br>111.0<br>132.2<br>76.4	(31)	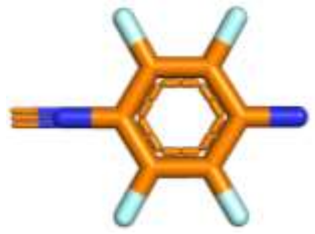
FUGVAE<br>CCDC 152633	32.	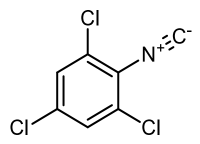	3.4<br>2.7<br>3.2	101.8<br>156.2<br>127.4	(32)	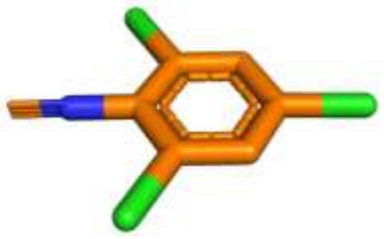
FUMWIV<br>CCDC 1047214	33.	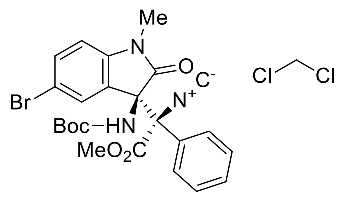	2.6	155.9	(33)	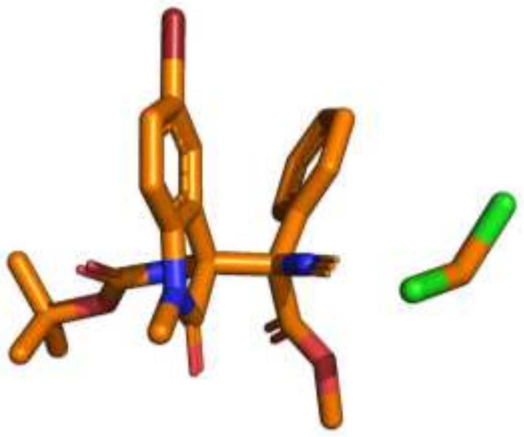

FUPPUE<br>CCDC 1839577	34.	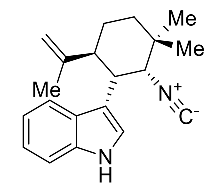	2.6	145.7	(34)	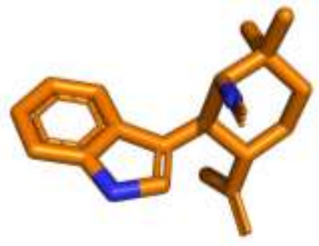
GESJOD<br>CCDC 116639	35.	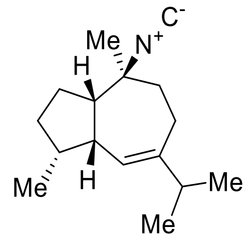	2.9	110.6	(35)	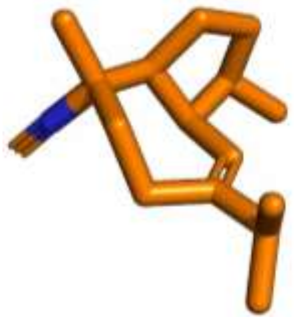
GEZMOR<br>CCDC 2216961	36.	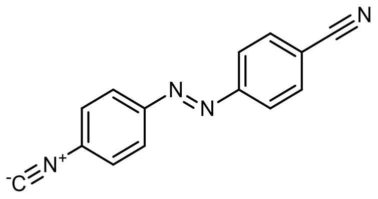		155.4<br>136.9	(36)	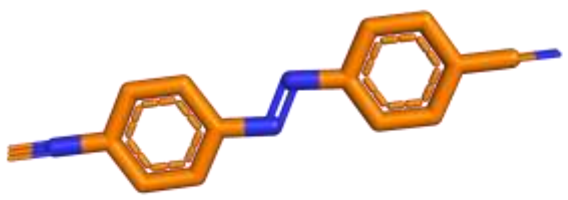
GUNVAN<br>CCDC 743242	37.	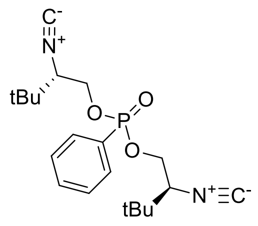	2.8<br>2.8	77.8<br>148.0	(37)	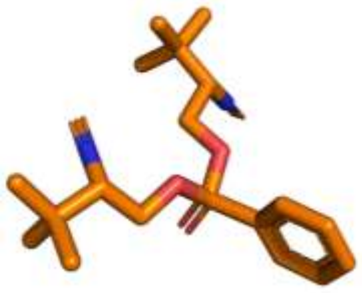

HAJQOB<br>CCDC 1521778	38.	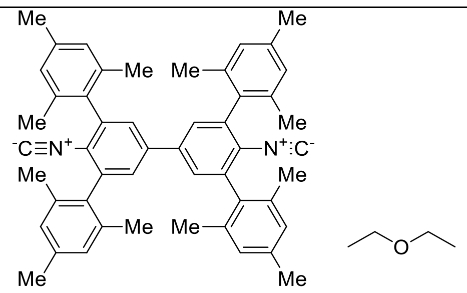	2.8<br>3.2<br>2.7<br>2.8<br>2.8	111.8<br>88.6<br>103.2<br>96.1<br>138.3	(38)	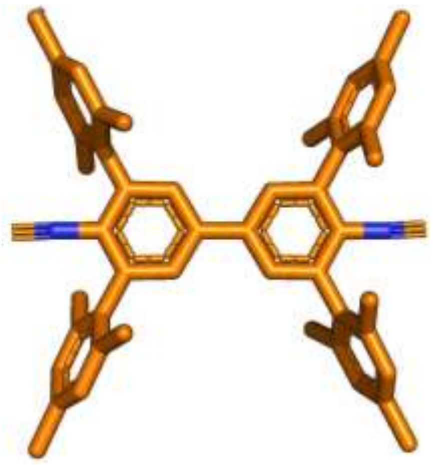
HANQAO<br>CCDC 100935	39.	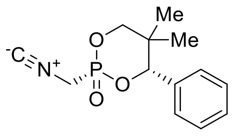	2.8<br>2.8	136.1<br>87.9	(39)	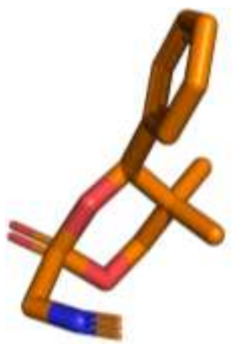
HANQES<br>CCDC 116846	40.	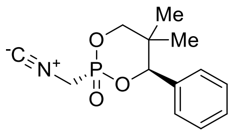	2.8<br>3.3	95.6<br>103.8	(39)	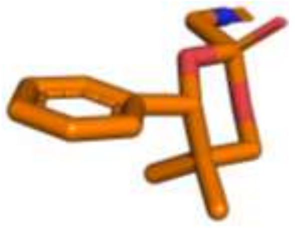
HEBNIP<br>CCDC 2112911	41.	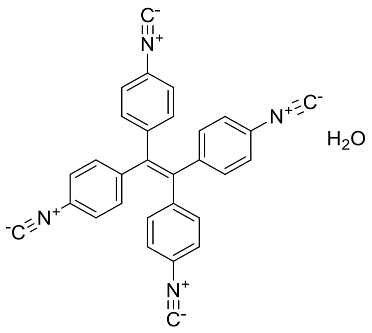	3.3<br>2.6<br>2.7<br>2.7<br>3.1	106.5<br>104.1<br>147.1<br>147.7<br>85.0	(243)	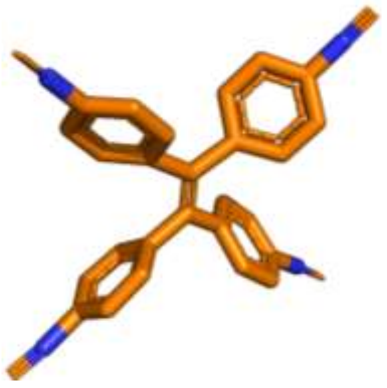
HEXZIT<br>CCDC 1175506	42.		2.9<br>3.0<br>2.2	109.0<br>150.5<br>152.8	(41)	

		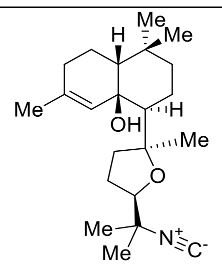				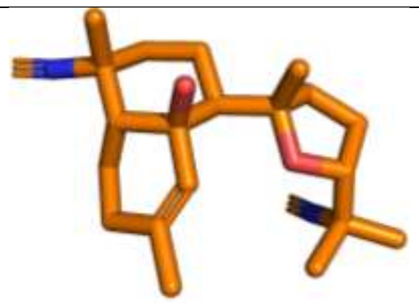
HIBRAO<br>CCDC 1820014	43.	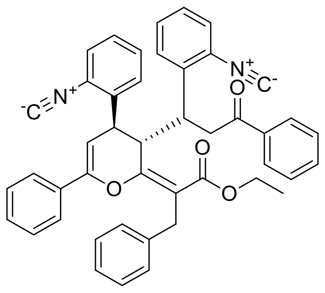	3.4<br>2.8<br>2.9	120.2<br>125.3<br>150.5	(40)	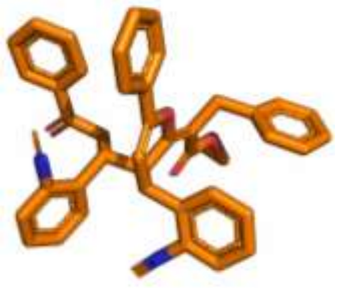
IBZICN<br>CCDC 1179408	44.	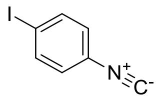	3.2	180.0	(42)	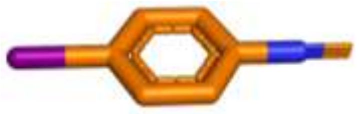
ICAMPH<br>CCDC 117948	45.	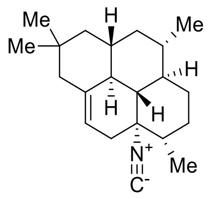	2.8<br>2.7<br>2.9	127.4<br>138.3<br>91.7	(43)	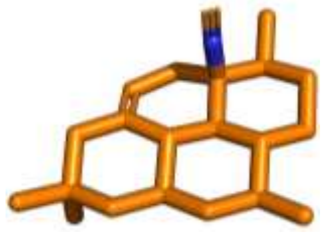
ICEPAM<br>CCDC 1179497	46.	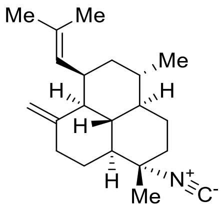	2.8	109.0	(43)	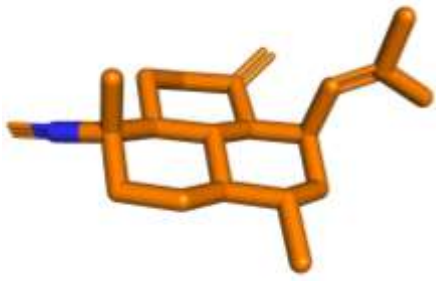

ICIYAU<br>CCDC 174493	47.	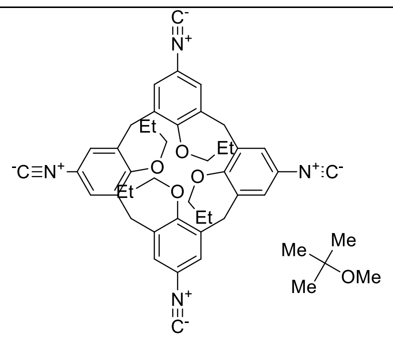	2.9<br>2.9<br>2.8<br>2.8<br>2.9<br>2.7	118.1<br>112.7<br>134.9<br>129.0<br>167.4<br>120.1	(44)	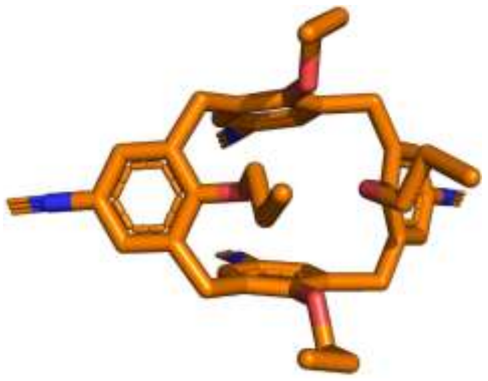
ICPUPK<br>CCDC 1179593	48.	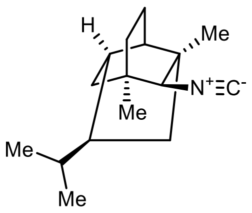	2.6<br>2.8	151.2<br>126.3	(45)	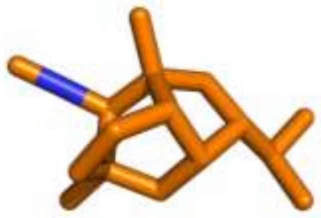
IMIJUJ<br>CCDC 226954	49.	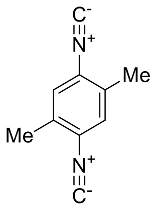	2.7	141.0	(46)	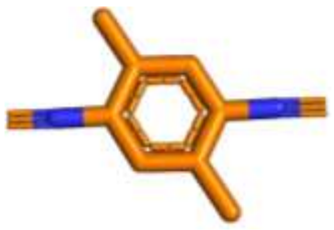
IMIKAQ<br>CCDC 226955	50.	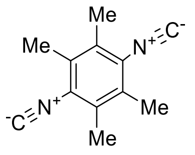	2.6<br>2.8<br>2.9	114.9<br>105.2<br>94.4	(47)	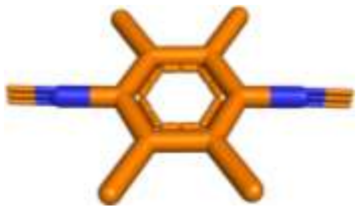
IZIROB<br>CCDC 2085631	51.	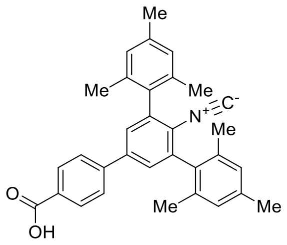	2.7	114.0	(48)	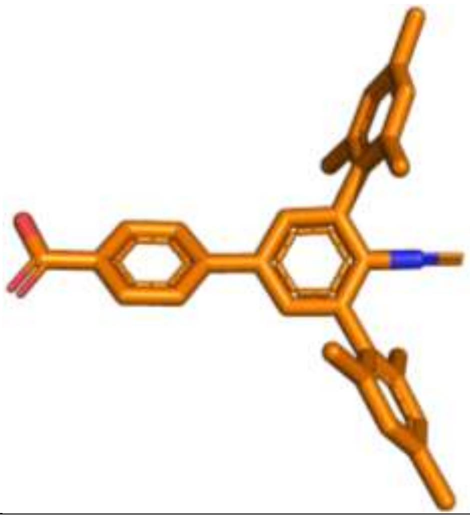

JABHUS<br>CCDC 1060616	52.	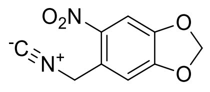	3.4<br>2.7<br>2.8	117.8<br>150.0<br>122.3	(49)	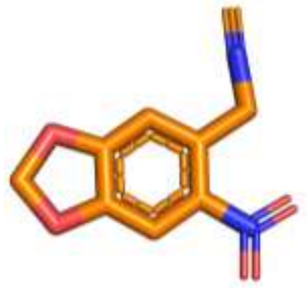
JAJNOY<br>CCDC 211338	53.	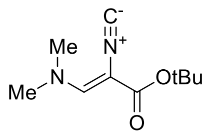	3.4	130.0	(50)	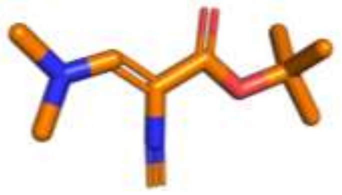
JEBTOC<br>CCDC 1556759	54.	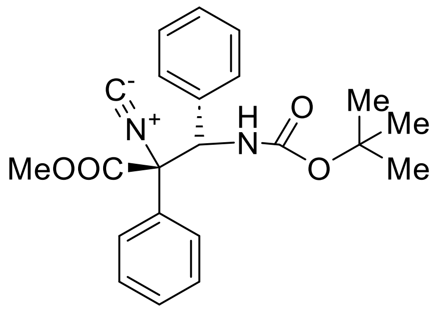	2.8	134.7	(51)	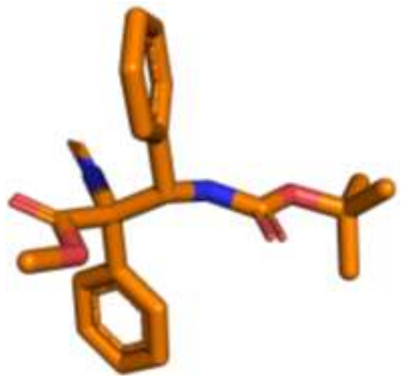
JEVSEM<br>CCDC 2155533	55.	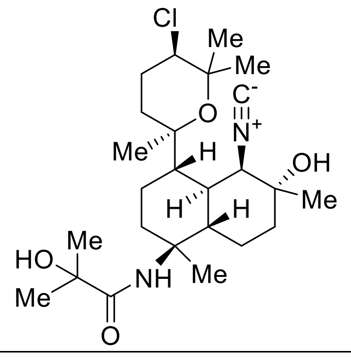	2.8<br>2.8<br>2.5<br>2.8	130.9<br>139.7<br>93.6<br>106.6	(52)	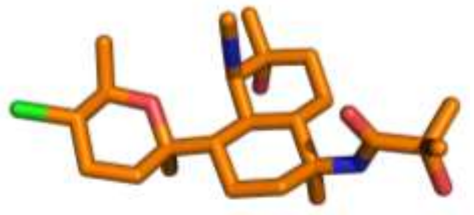

JEVSIQ<br>CCDC 2155535	56.	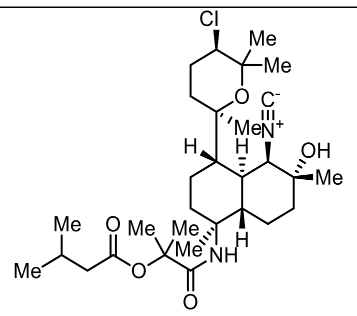	2.8<br>2.8<br>2.8<br>2.9<br>2.9<br>2.5<br>2.5<br>2.5<br>2.6	137.1<br>129.0<br>129.3<br>136.7<br>102.0<br>167.6<br>168.2<br>172.8<br>174.0	(52)	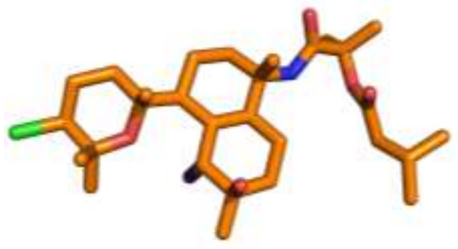
JIFFIN<br>CCDC 1185999	57.	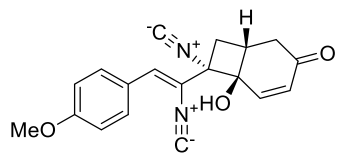	2.3<br>2.9<br>3.0	158.2<br>162.4<br>156.8	(53)	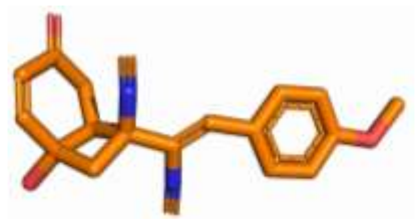
JUBJIZ<br>CCDC 1189920	58.	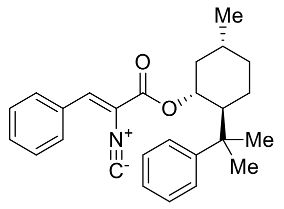	2.9<br>2.9	119.0<br>133.4	(54)	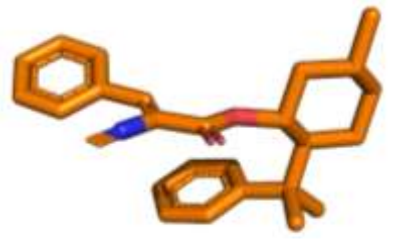
JUJTOA<br>CCDC 1987281	59.	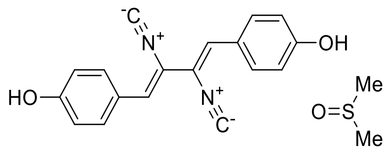	2.9<br>2.8	125.6<br>143.0	(55)	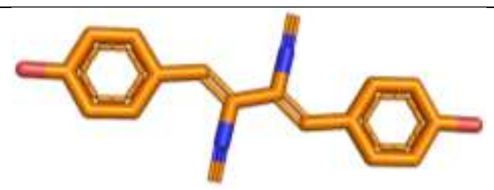
KACFOM<br>CCDC 1424362	60.	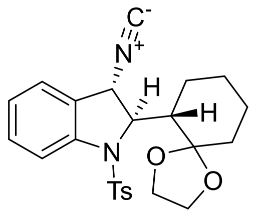	2.8<br>2.5	84.4<br>160.8	(56)	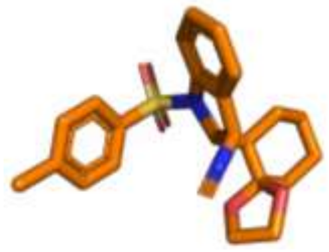
KAGFEE<br>CCDC 148386	61.	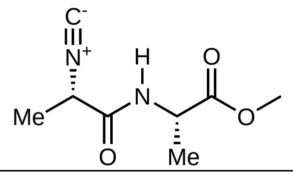	2.9	139.2	(57)	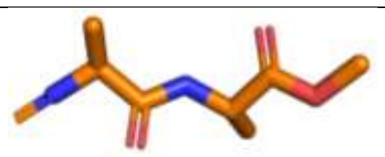
KAGVAT<br>CCDC 2007072	62.		3.3<br>2.8<br>2.8	109.8<br>105.5<br>140.0	(57)	

		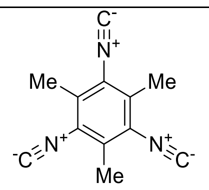	2.8<br>2.9<br>2.8<br>2.9	109.5<br>94.4<br>164.9<br>90.6		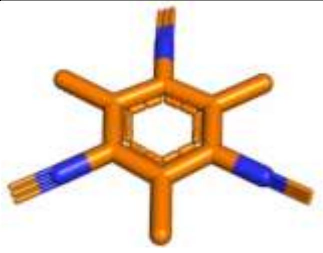
KEDGIM<br>CCDC 1482775	63.	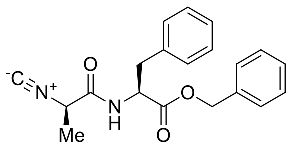	3.2<br>2.5<br>3.4<br>2.6	118.2<br>107.6<br>94.9<br>87.2	(58)	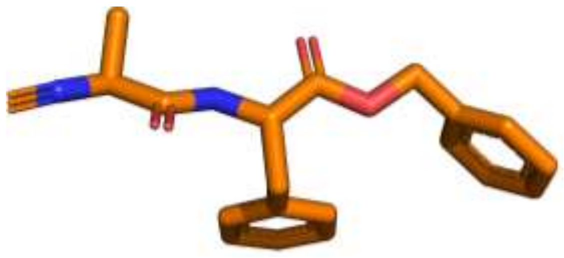
KEJDIP<br>CCDC 1547517	64.	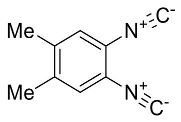	2.8<br>2.8	95.8<br>103.2	(59)	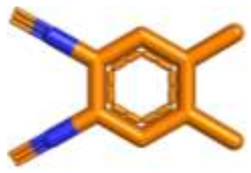
KEJROK CCDC 2178993	65.	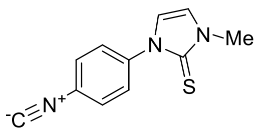	2.8<br>2.8<br>2.7<br>2.7	171.5<br>171.7<br>122.1<br>120.9	(60)	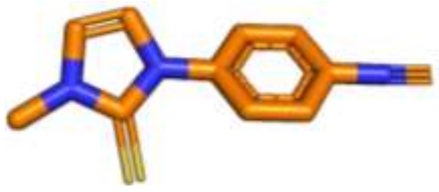
KEPPOM<br>CCDC 917995	66.	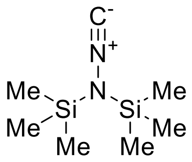	2.8	171.8	(61)	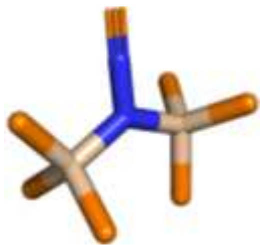
KIBHIN<br>CCDC 623052	67.	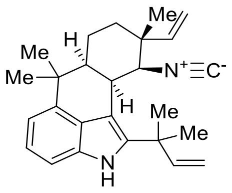	2.7<br>3.2<br>2.4	111.7<br>161.9<br>160.0	(6263)	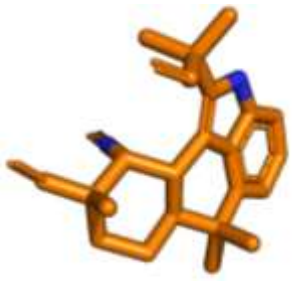

KIGNOF<br>CCDC 948968	68.	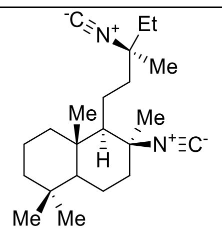	2.9<br>2.9<br>2.8<br>2.8<br>2.6<br>2.6	98.6<br>98.1<br>160.5<br>160.4<br>152.8<br>152.1	(64)	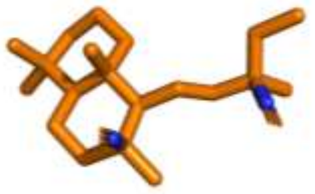
KIGNUL<br>CCDC 949094	69.	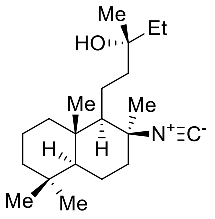	2.8<br>2.8<br>2.7	148.8<br>152.6<br>144.7	(64)	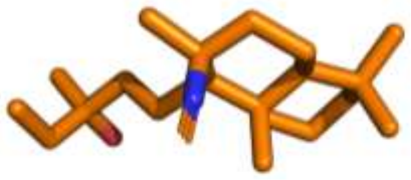
KINTUW<br>CCDC 1197232	70.	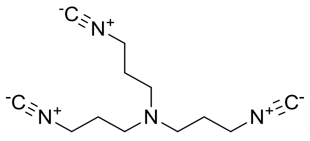	2.8	143.6	(65)	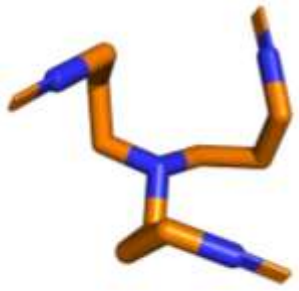
KITBOG<br>CCDC 932205	71.	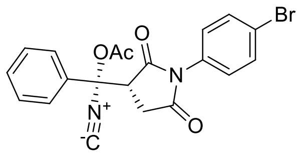	2.9<br>2.7	96.7<br>153.4	(66)	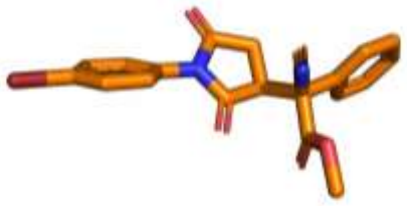
KITBOG01<br>CCDC 844936	72.	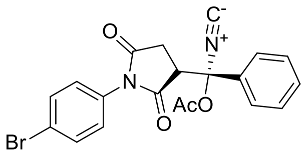	2.7<br>2.8	158.1<br>96.6	(67)	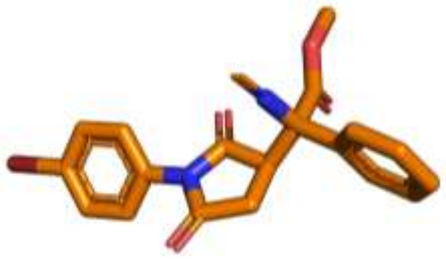
KOTYEX<br>CCDC 1199689	73.	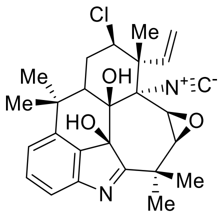	2.9	123.9	(68)	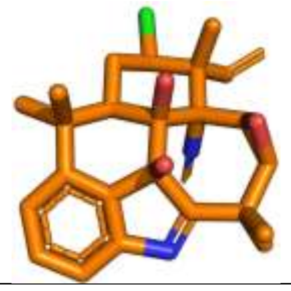

KOVPUG<br>CCDC 1199763	74.	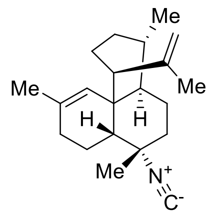	2.9<br>2.9	113.1<br>138.0	(69)	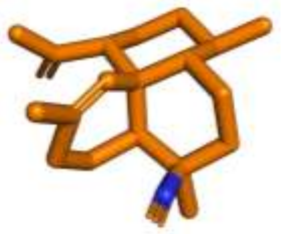
LACTEP<br>CCDC 225216	75.	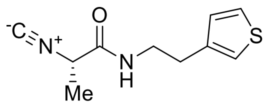	2.8	97.4	(70)	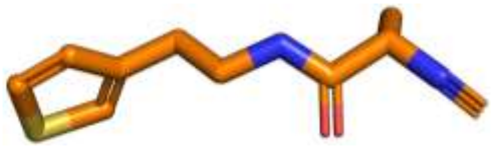
LAVQUY<br>CCDC 2091122	76.	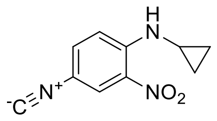	3.2<br>2.8<br>3.2<br>2.7<br>2.4<br>2.8<br>2.9	173.4<br>169.3<br>132.7<br>139.2<br>169.1<br>124.5<br>97.5	(71)	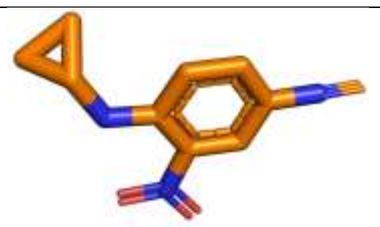
LAVRAF<br>CCDC 2091123	77.	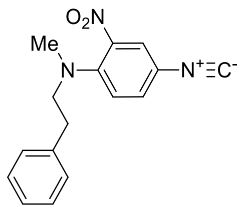	2.8	153.8	(71)	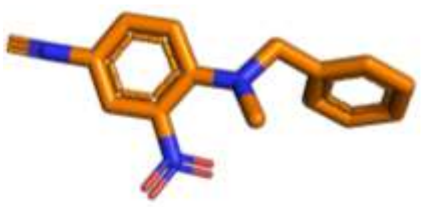
LEGROE<br>CCDC 1204903	78.	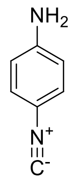	2.8<br>2.4	89.4<br>173.2	**(Error! Reference source not found.)**	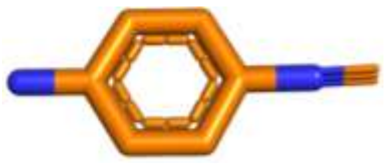
LUKVUK<br>CCDC 1063415	79.	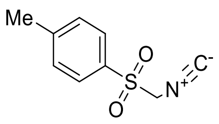	2.7<br>2.9	159.1<br>100.8	(73)	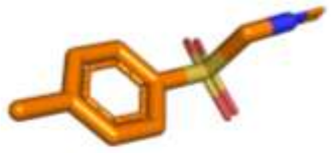
MADKAF<br>CCDC 756983	80.	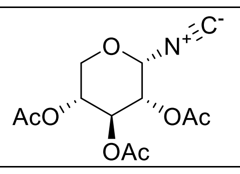	2.8<br>2.8<br>3.3<br>3.3	121.2<br>121.4<br>99.9<br>100.4	(74)	

						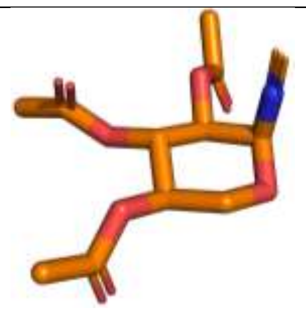
MELFOY<br>CCDC 147927	81.	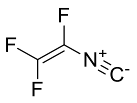	3.3<br>3.3	140.2<br>162.4	(75)	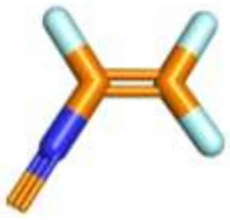
MERROR<br>CCDC 612759	82.	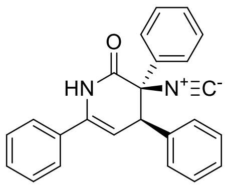	2.9<br>2.7	13.6<br>104.9	(76)	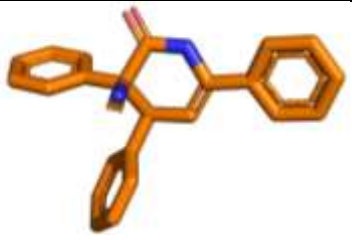
MESRAG<br>CCDC 1812522	83.	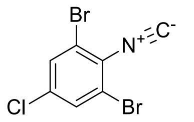	3.1	135.9	(77)	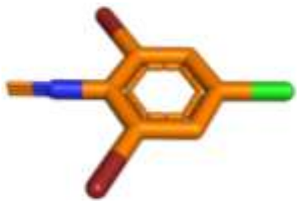
MESRIO<br>CCDC 1581218	84.	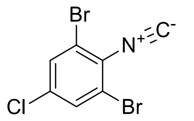	3.1<br>3.2	134.2<br>103.0	(78)	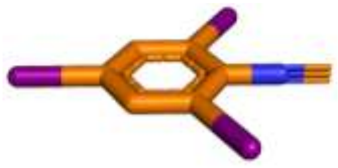
MEWNOT<br>CCDC 935506	85.	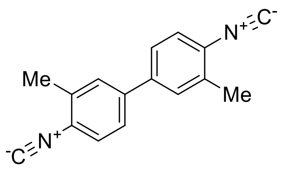	2.8<br>2.9<br>2.8	163.3<br>115.4<br>143.7	(79)	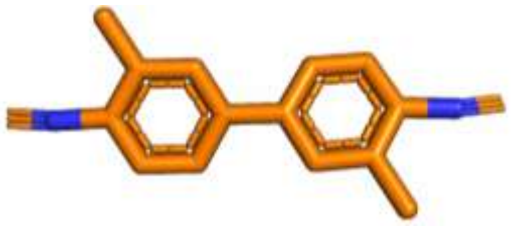
MIGGEO<br>CCDC 153962	86.		2.9<br>2.8<br>2.7<br>2.8	141.2<br>142.7<br>124.6<br>126.2	(80)	

		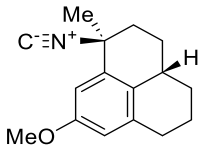				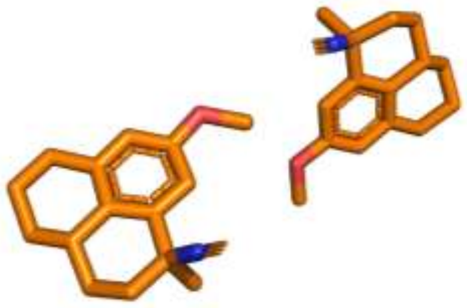
MIYTEW<br>CCDC 1872444	87.	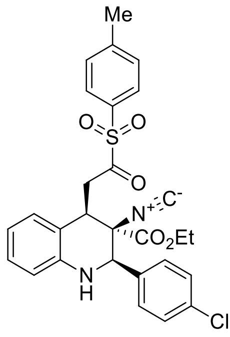	2.7	127.4	(81)	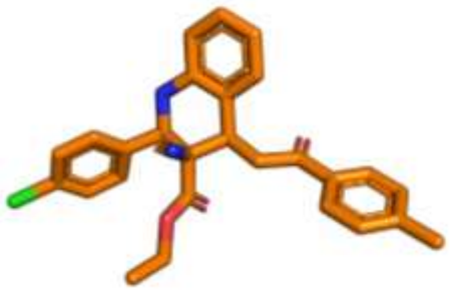
MOFLEZ<br>CCDC 624648	88.	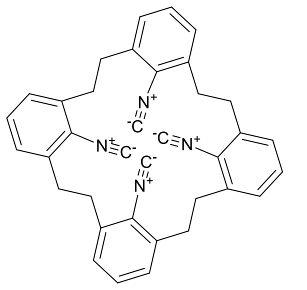	3.3<br>2.7<br>2.9	83.9<br>160.8<br>78.4	(82)	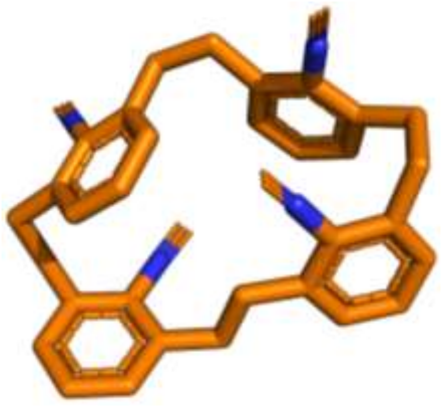
MOPJUW<br>CCDC 192968	89.	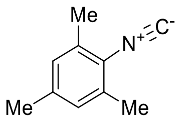	2.8	135.1	(83)	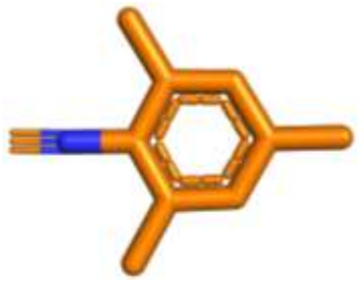

MUBXUE<br>CCDC 1035106	90.	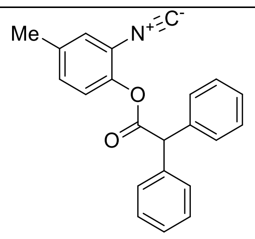	2.8<br>2.7	110.0<br>158.6	(84)	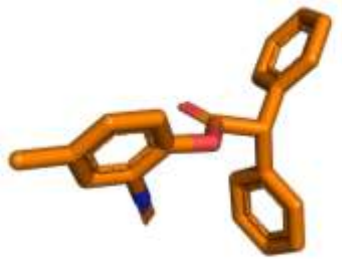
MURXIJ<br>CCDC 2002330	91.	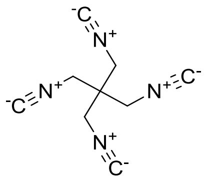	2.6	146.7	(85)	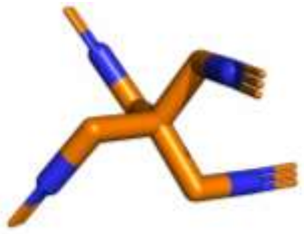
NEBQAM<br>CCDC 100304	92.	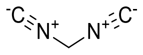	3.4<br>3.4<br>2.7<br>2.8<br>2.9<br>2.7	12.6<br>169.9<br>141.9<br>134.<br>101.8<br>157.1	(86)	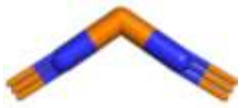
NEBQAM01<br>CCDC 120952	93.	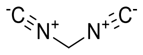	3.4<br>3.4<br>2.7<br>2.8<br>29<br>2.7	126.5<br>169.9<br>141.9<br>134.6<br>101.2<br>158.0	**(Error ! Reference source not found .)**	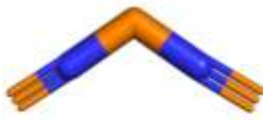
NIHXAF<br>CCDC 945642	94.	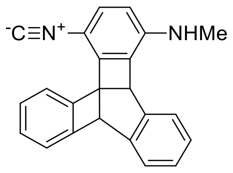	3.4<br>2.4<br>2.8<br>2.6<br>3.1	115.5<br>156.0<br>128.2<br>115.8<br>149.5	(87)	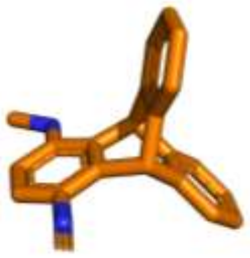
OGEWIH<br>CCDC 758871	95.	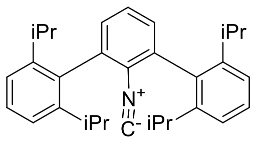 iPr= isopropyl group	2.7<br>2.8	118.6<br>117.9	(89)	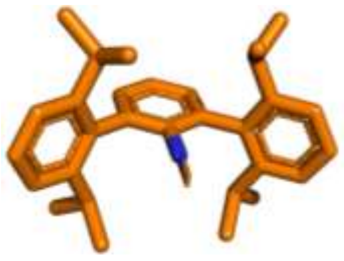
OJOYES<br>CCDC 751206	96.		2.8	151.2	(90)	

		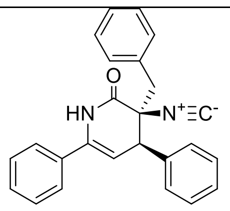				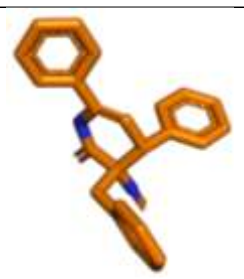
OLEWOS<br>CCDC 753606	97.	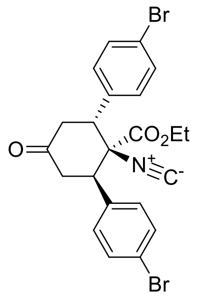	2.7	109.8	(91)	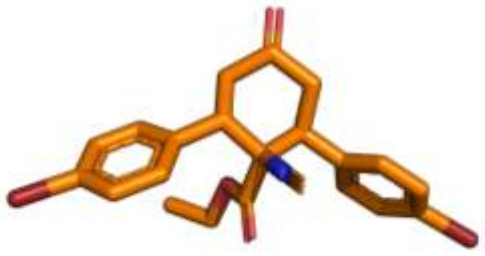
OLEWOS01<br>CCDC 1011009	98.	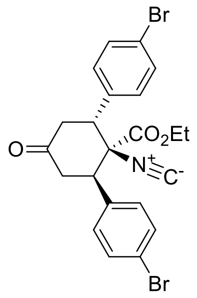	2.7	109.8	(92)	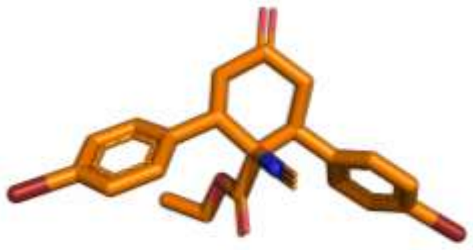
OSIGED<br>CCDC 833707	99.	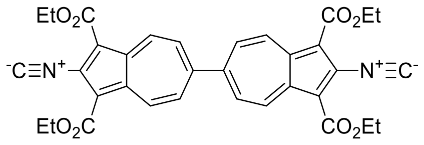	2.7	147.1	(93)	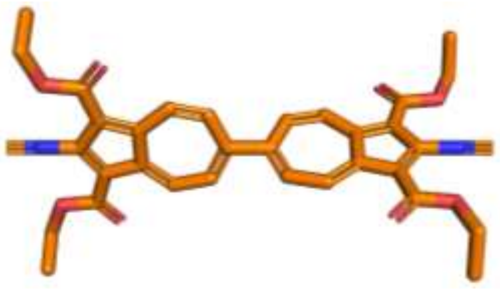
OZATUG<br>CCDC 1491066	100.	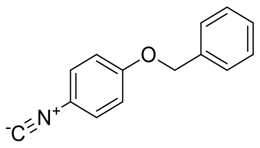	2.9<br>2.8	102.3<br>143.8	(94)	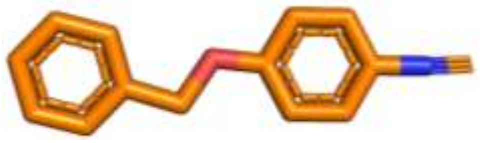
PEKWIN<br>CCDC 898814	101.	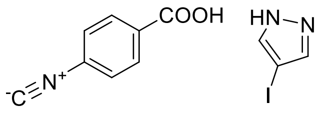	3.2<br>2.7	168.3<br>116.9	(95)	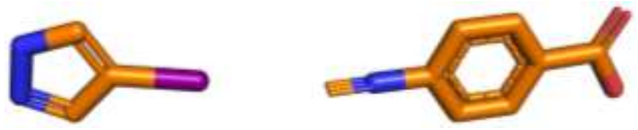

QAPHOG<br>CCDC 842072		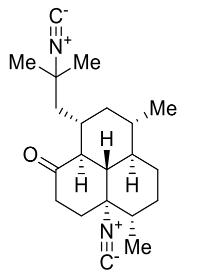	2.8<br>2.9	113.7<br>108.1	(96)	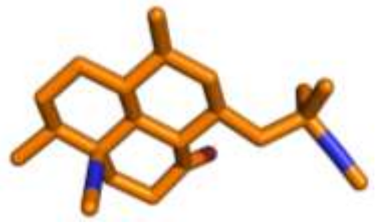
QUXWOY<br>CCDC 2005977	103.	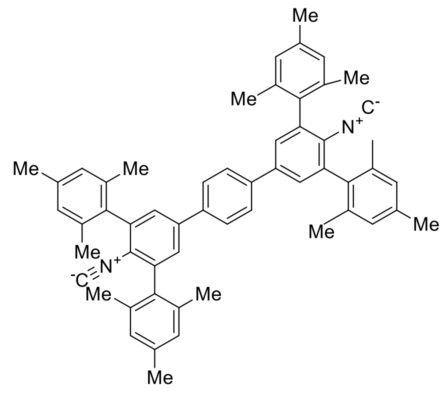	2.7<br>2.9<br>2.7<br>2.7	158.7<br>115.8<br>103.3<br>155.1	(97)	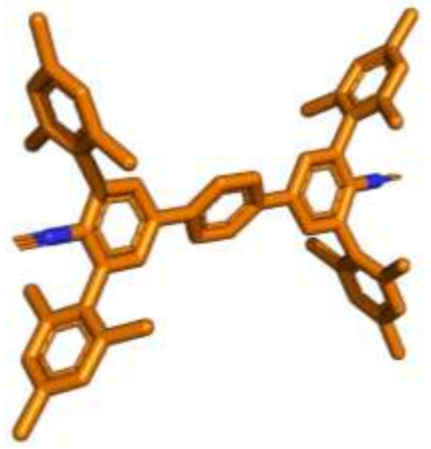
RECPUN<br>CCDC 1566922	104.	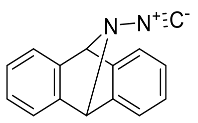	2.6	110.6	(98)	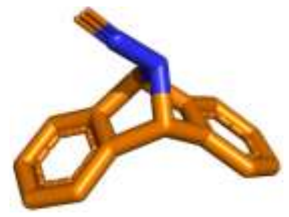
REMLIG<br>CCDC 867331	105.	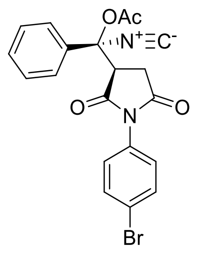	2.5<br>3.2<br>3.1	132.0<br>101.1<br>128.3	(99)	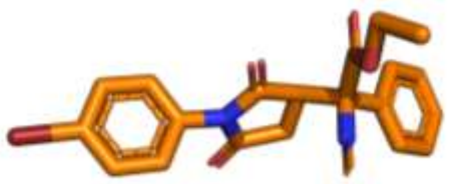
RIXDEK<br>CCDC 1869943	106.	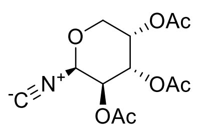	2.7<br>3.2<br>3.3<br>2.9<br>2.8	125.2<br>140.0<br>99.5<br>126.0<br>113.8	(99)	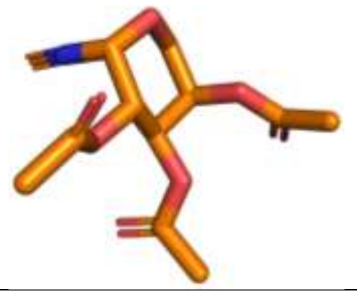

RIXDIO<br>CCDC 1869944	107.	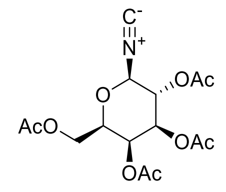	2.9<br>2.9	147.7<br>155.1	(9)	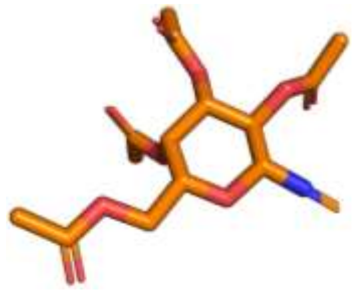
RIXDUA<br>CCDC 1869946	108.	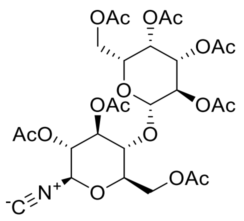	3.3	172.0	(9)	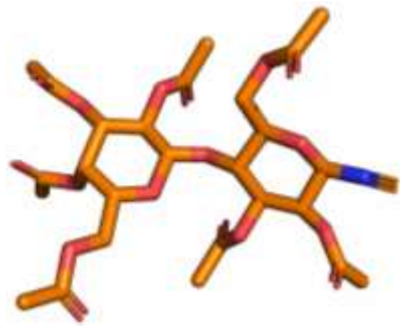
RIXFEM<br>CCDC 1870510	109.	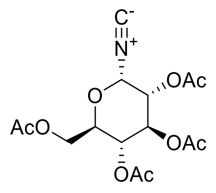	2.6	119.6	(9)	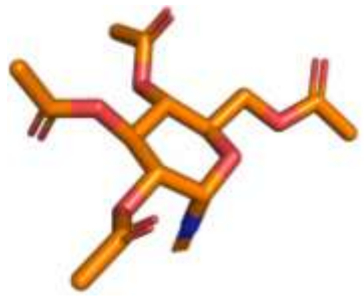
ROBJEX<br>CCDC 1251089	110.	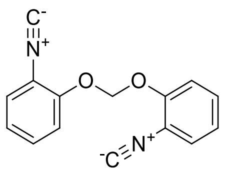	3.3<br>2.7<br>2.8<br>2.9<br>2.8<br>2.9<br>2.7	93.6<br>112.2<br>114.9<br>119.3<br>114.9<br>119.1<br>112.2	(100)	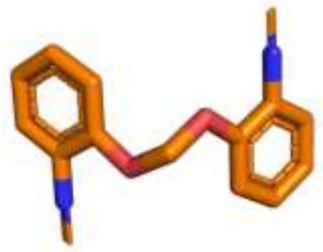
ROBJEX01<br>CCDC 180914	111.	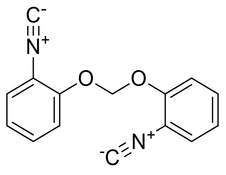	3.3	93.6	(101)	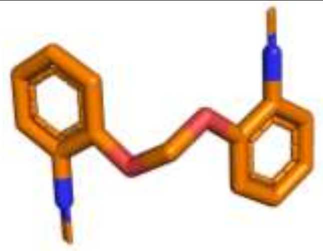

RODJAX<br>CCDC 988359	112.	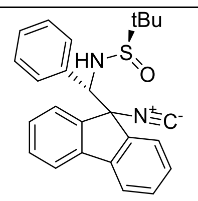	2.8	109.4	(102)	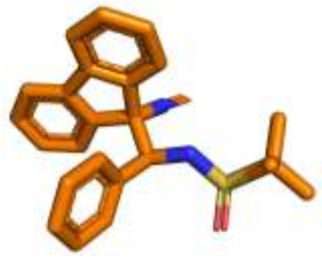
ROJYAQ<br>CCDC 1251532	113.	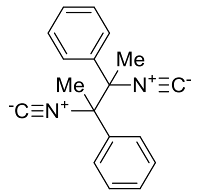	2.9<br>2.8	142.3<br>130.8	(103)	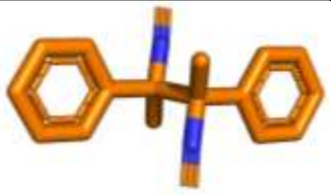
ROMNUE<br>CCDC 991945	114.	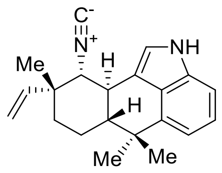	2.4<br>3.1	154.4<br>149.1	(104)	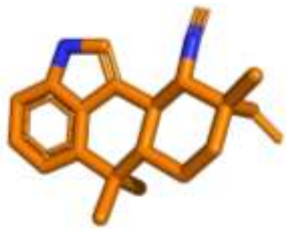
RUHGEG<br>CCDC 125242	115.	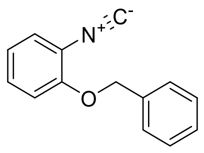	2.8	154.5	(105)	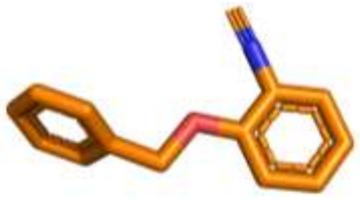
SAWBUP<br>CCDC 836916	116.	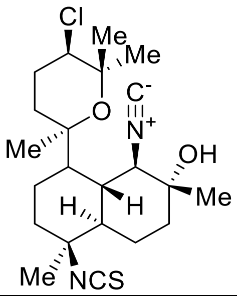	2.2<br>3.0	152.9<br>152.1	(106)	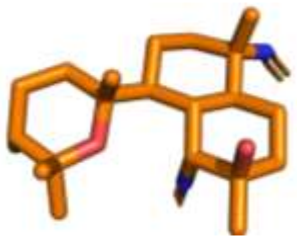
SAWCAW<br>CCDC 836917	117.	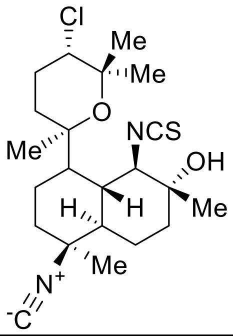	2.2<br>2.7<br>2.8<br>2.7<br>3.0	157.3<br>97.3<br>86.2<br>90.6<br>157.3	(106)	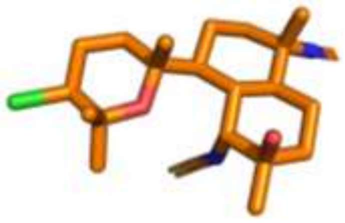
SAZPUF<br>CCDC 279551	118.		2.7<br>2.8	139.4<br>134.4	(107)	

		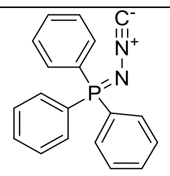	2.7	95.6		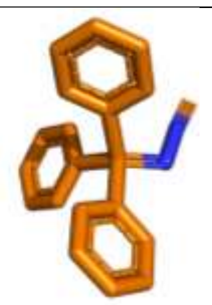
SEKHEW<br>CCDC 294875	119.	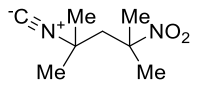	2.9	128.9	(108)	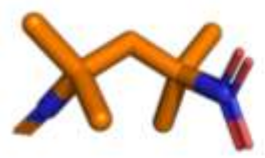
SIDBIQ<br>CCDC 1258627	120.	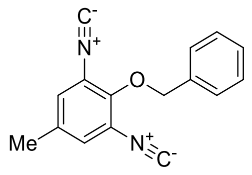	2.9<br>2.8	124.2<br>117.4	(109)	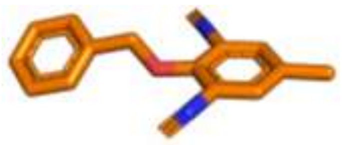
SUDGED<br>CCDC 1263629	121.	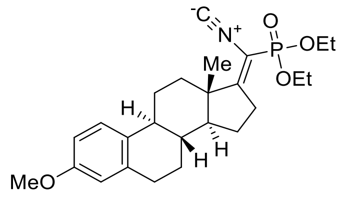	2.9<br>2.9<br>2.9<br>2.8	148.2<br>135.0<br>145.1<br>116.5	(110)	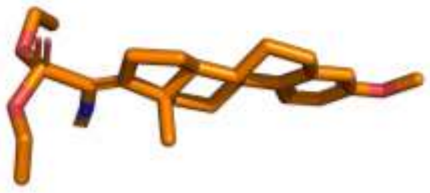
SUSYIP<br>CCDC 755663	122.	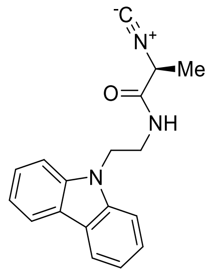	2.8<br>2.7<br>2.6	96.6<br>94.0<br>165.7	(111)	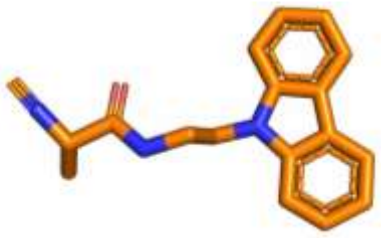
SUWJID<br>CCDC 131749	123.	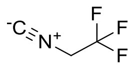	2.6	150.4	(112)	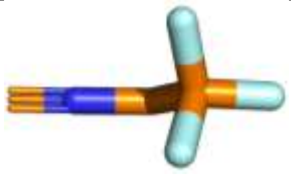
TAYGUW<br>CCDC 273844	124.		2.8<br>2.3<br>3.1	140.1<br>158.4<br>151.9	(113)	

		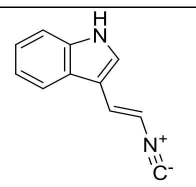				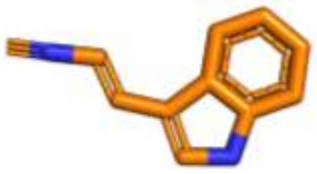
TBZINT<br>CCDC 1267703	125.	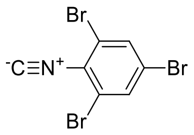	3.3<br>3.2<br>3.2<br>3.5	106.3<br>132.4<br>134.0<br>97.4	(114)	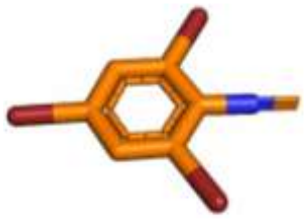
TBZINT01<br>CCDC 1445499	126.	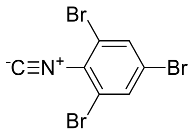	3.2<br>3.1<br>3.2<br>3.5	105.2<br>134.0<br>133.2<br>99.8	(115)	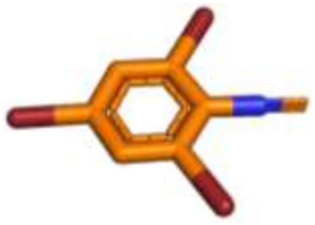
TEKSEH<br>CCDC 127963	127.	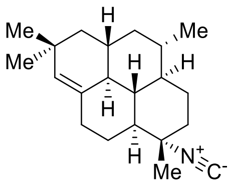	2.6<br>2.8<br>2.8	108.7<br>125.9<br>125.2	(116)	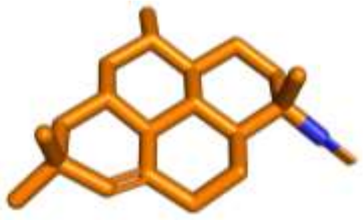
TEKSIL<br>CCDC 127964	128.	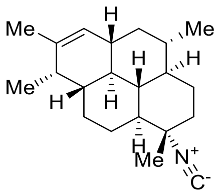	2.8	84.8	(116)	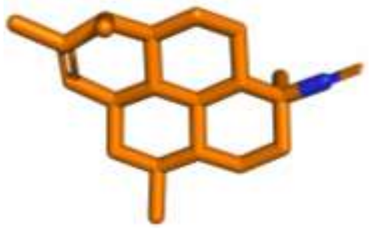
TEKSOR<br>CCDC 127965	129.	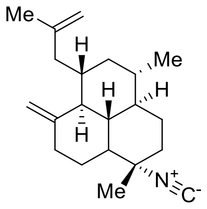	2.8	161.8	(116)	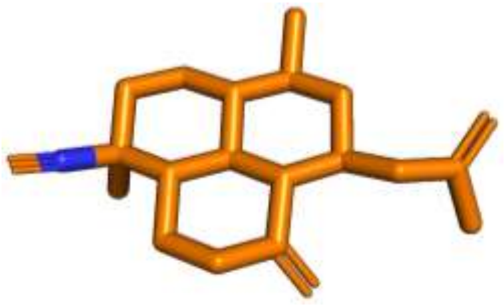
TEKSUX<br>CCDC 127966	130.		2.9<br>2.9	118.0<br>165.6	(116)	

		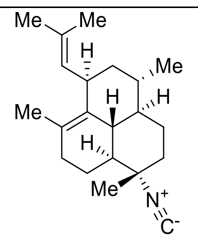				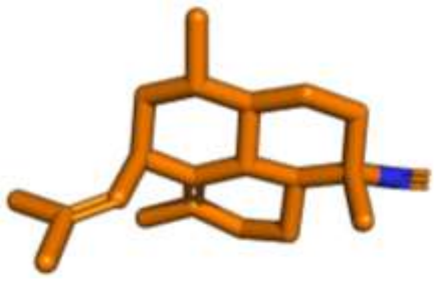
TRIVIR01<br>CCDC 1275717	131.	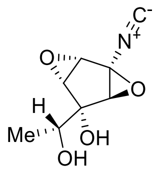	3.0	109.2	(117)	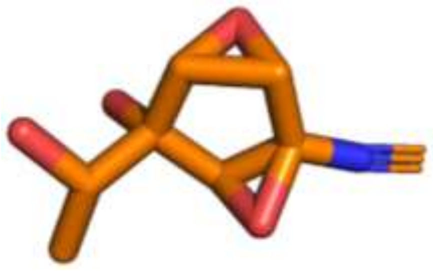
UHANOJ<br>CCDC 1955600	132.	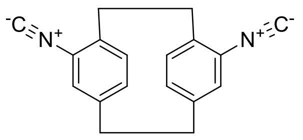	2.8<br>2.9<br>2.7<br>2.9	142.3<br>124.0<br>140.1<br>95.7	(118)	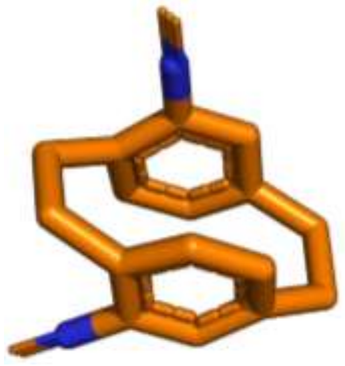
UJEPEG<br>CCDC 1443750	133.	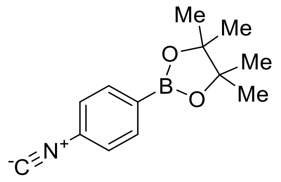	2.9	149.0	(119)	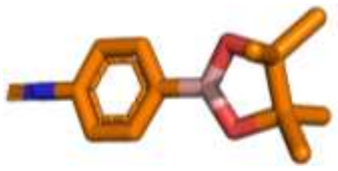
VEFVUA<br>CCDC 1573845	134.	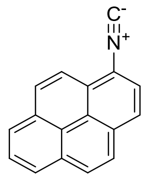	2.8	115.3	(120)	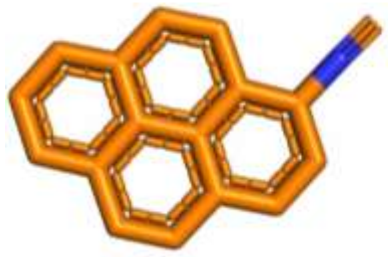
VEPBIF<br>CCDC2145549	135.		3.3<br>2.7<br>2.6	96.8<br>93.2<br>122.5	(121)	

		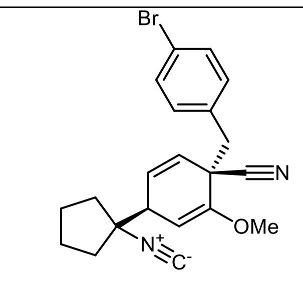				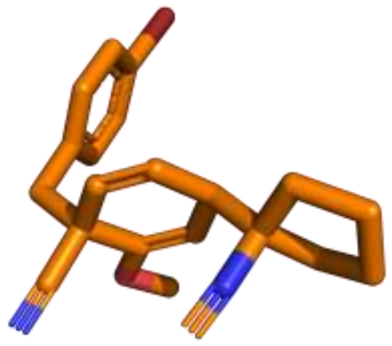
WABXOQ<br>CCDC 2022542	136.	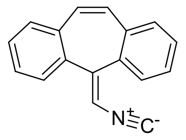	2.8<br>2.7	111.2<br>126.3	(122)	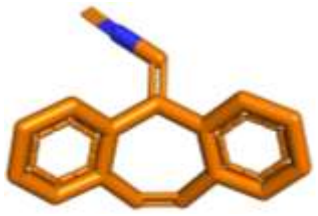
WAGMIC<br>CCDC 722563	137.	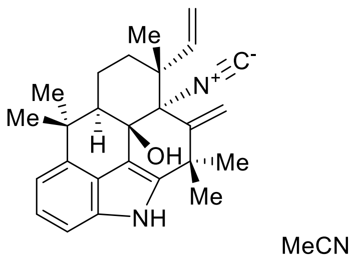	2.9<br>2.8<br>2.2	131.7<br>129.6<br>135.9	(123)	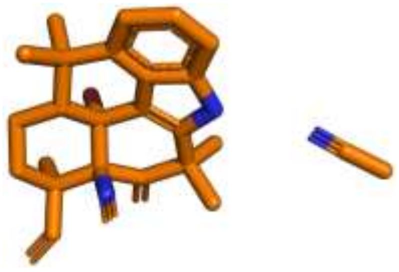
WAYXOL<br>CCDC 867305	138.	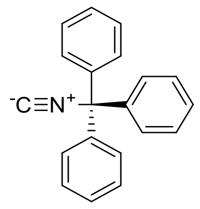	2.9	135.3	(124)	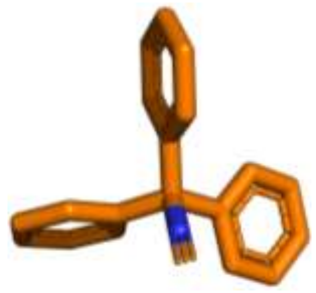
WOJPIV<br>CCDC 707281	139.	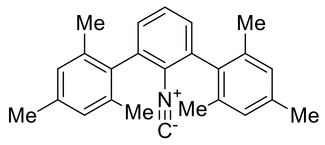	2.8<br>2.8<br>2.8	143.0<br>115.4<br>96.5	(124)	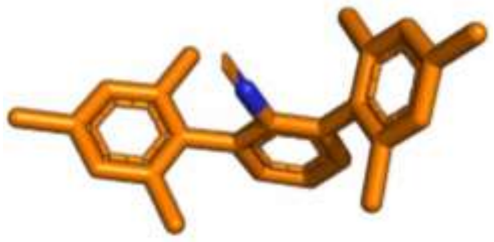
XAVRAO<br>CCDC 267942	140.		2.8	139.7	(125)	

		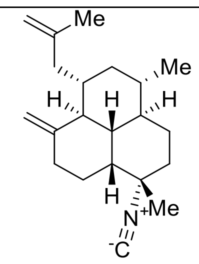				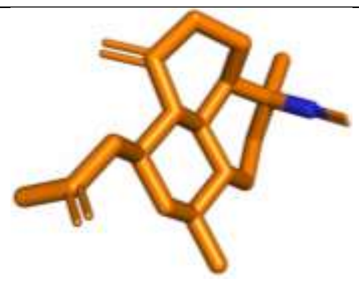
XAZQEW<br>CCDC 267942	141.	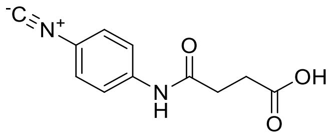	2.3<br>3.1<br>2.9	167.8<br>164.1<br>118.9	(126)	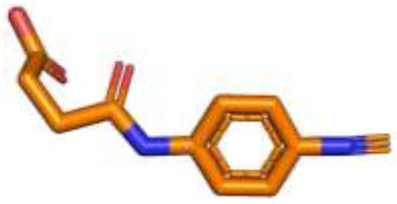
XEDRII<br>CCDC 601010	142.	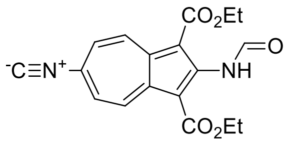	3.4	113.3	(127)	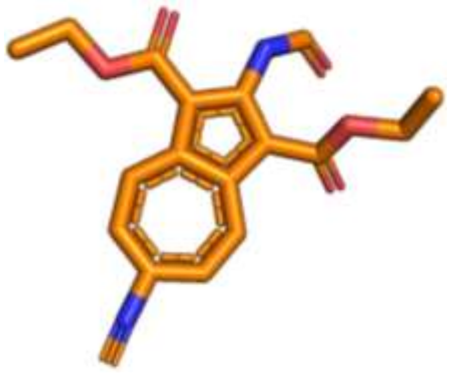
XERPET CCDC<br>2166676	143.	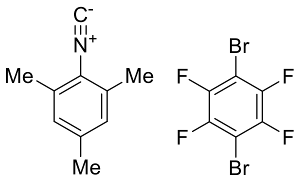	3.1	177.2	(128)	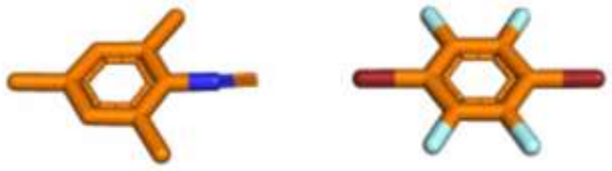
XEVRAV<br>CCDC 2125089	144.	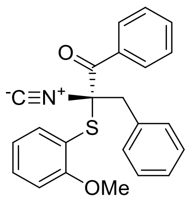	2.7<br>2.8	99.0<br>111.3	(129)	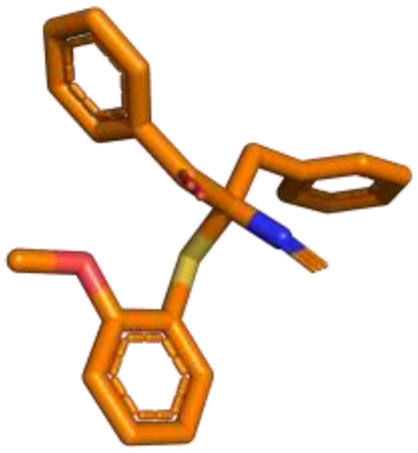
XIDYEO	145.		2.8	153.4	(130)	

CCDC 160930		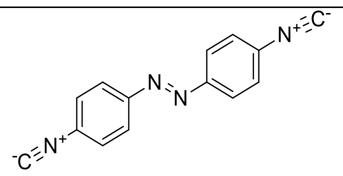	2.8	139.0		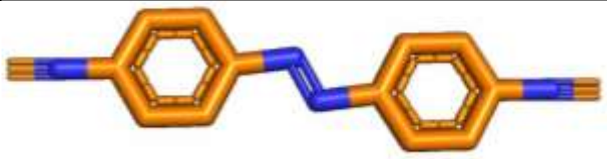
XODXEW<br>CCDC 1499073	146.	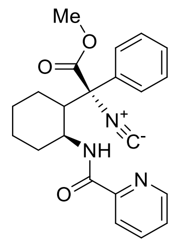	2.9	115.5	(131)	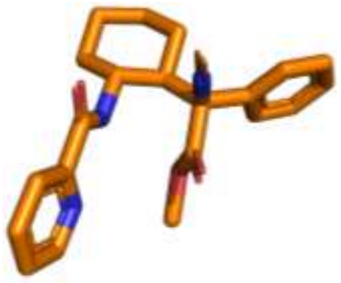
XOFBAX<br>CCDC 1008201	147.	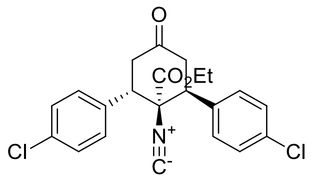	2.7	110.8	(132)	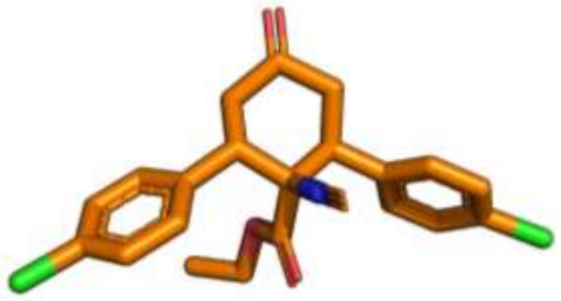
YEGMOQ<br>CCDC 2167174	148.	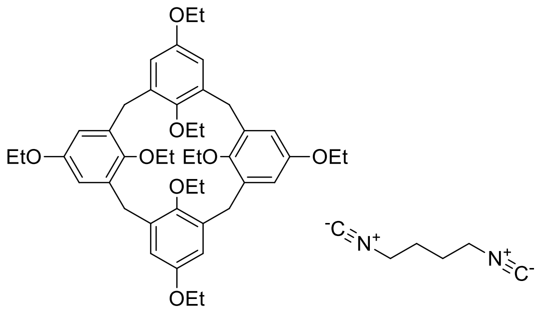	2.8<br>2.7<br>2.7<br>2.7<br>2.9	147.6<br>93.2<br>135.7<br>120.7<br>95.7	(133)	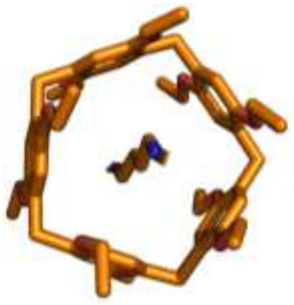
YEGMUW<br>CCDC 2167170	149.	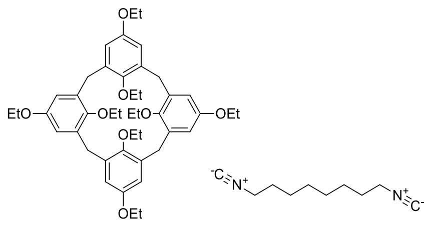	2.8<br>2.8	167.1<br>94.9	(133)	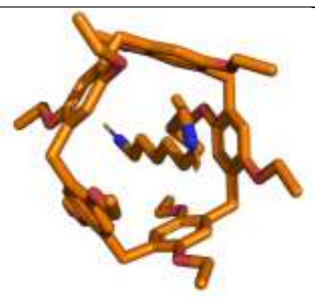

YEGNAD<br>CCDC 2167171	150.	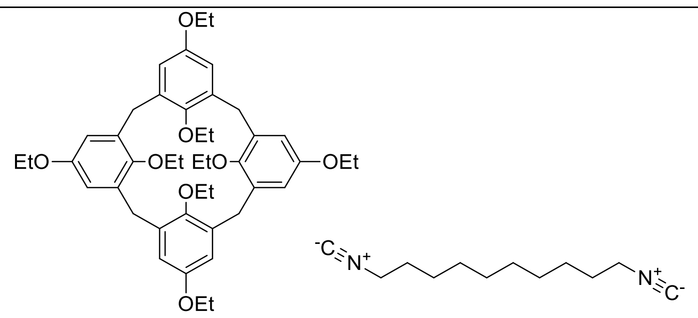	2.9	124.9	(133)	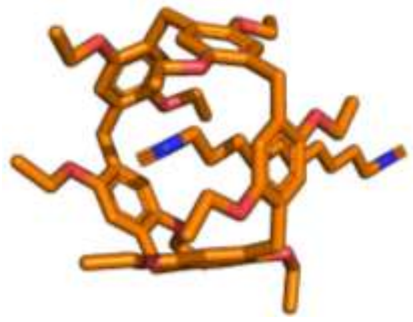
YEGNEH<br>CCDC 2167173	151.	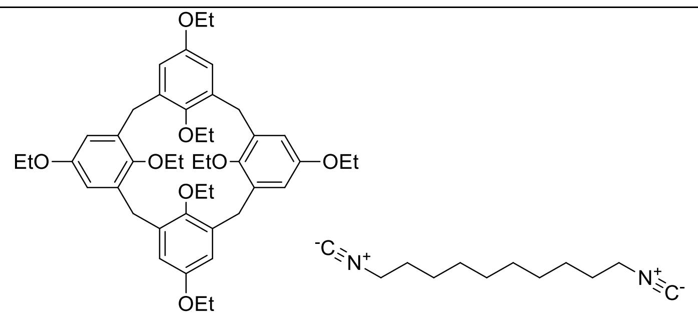	2.9	155.6	(133)	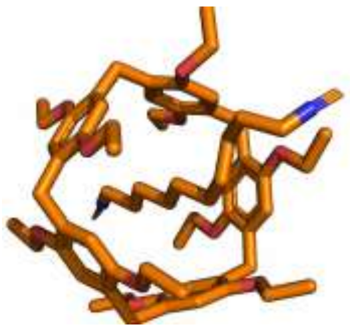
YEGNIL<br>CCDC 2167172	152.	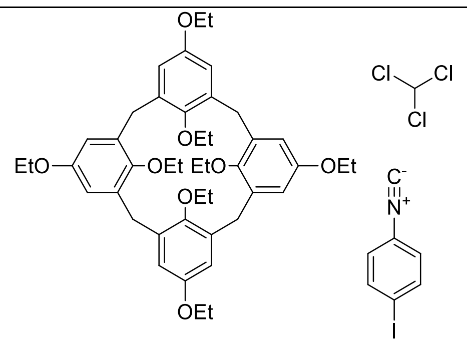	2.9	113.4	(133)	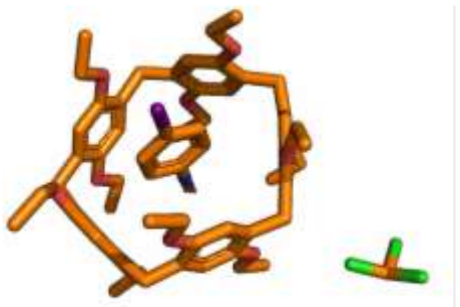
YINSEU<br>CCDC 649970	153.	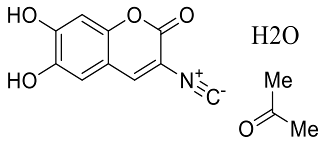	2.9<br>2.9	120.2<br>140.5	(134)	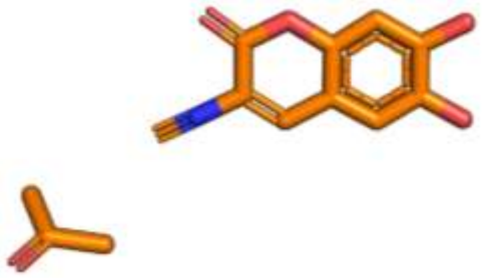
YUBTOE<br>CCDC 1306173	154.	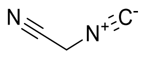	3.3<br>3.3<br>3.4<br>2.6<br>2.6<br>2.7<br>2.8<br>2.8	165.9<br>125.1<br>129.0<br>140.2<br>134.4<br>151.8<br>98.9<br>99.7	(1351<br>36)	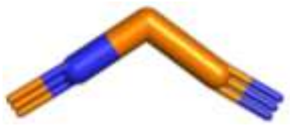
YUNZAJ	155.		2.3	137.7	(137)	

CCDC 716191		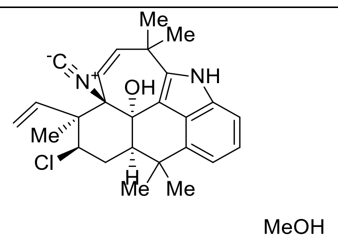	3.0	129.6		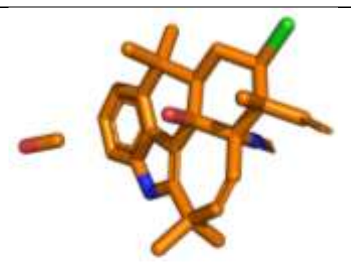
ZAFWOV<br>CCDC 1411352	156.	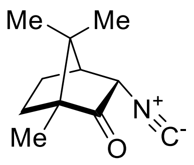	2.8	109.4	(138)	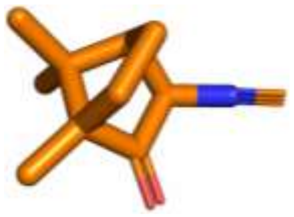
ZAMTEM<br>CCDC 130892	157.	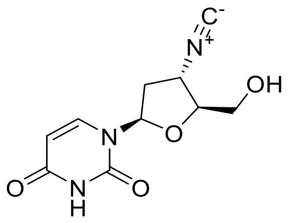	2.9<br>2.7	159.3<br>105.9	(139)	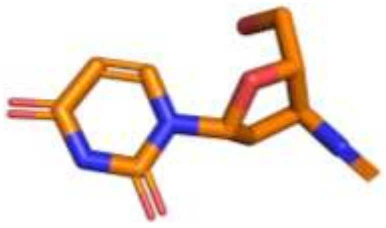
ZIRQID<br>CCDC 1047215	158.	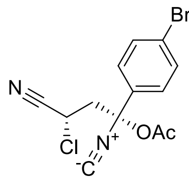	2.8<br>2.7	157.274<br>110.145	(140)	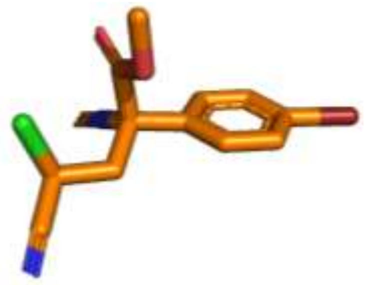
ZOYWAN<br>CCDC 1056657	159.	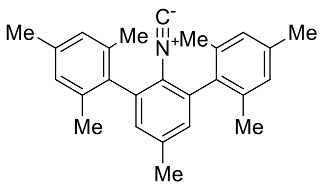	2.7	111.7	(141)	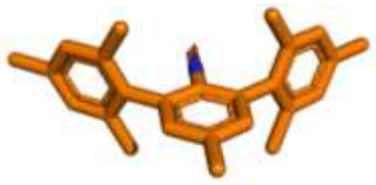
ZUTKIL<br>CCDC 1957698	160.	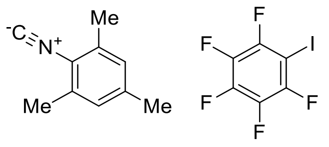	2.7<br>3.1	107.8<br>177.3	(142)	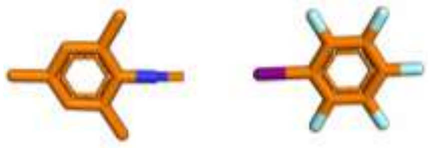
ZUTKIL01<br>CCDC 1981526	161.	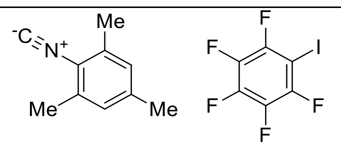	2.7<br>3.1	107.3<br>177.3	(142)	

						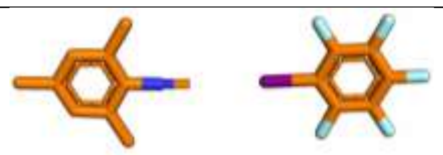
ZUTKOR<br>CCDC 1957699	162.	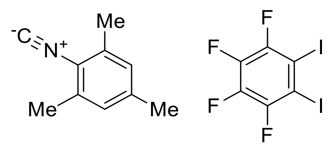	2.7<br>3.0	103.6<br>169.2	(142)	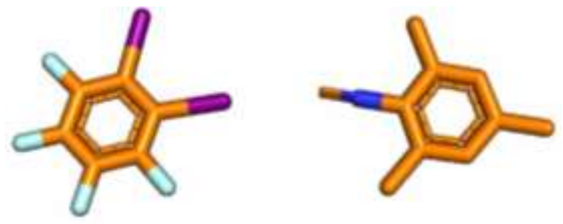
ZUTKUX<br>CCDC 1957700	163.	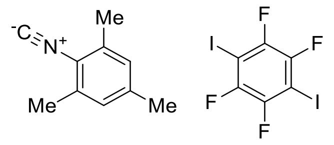	3.1	165.3	(142)	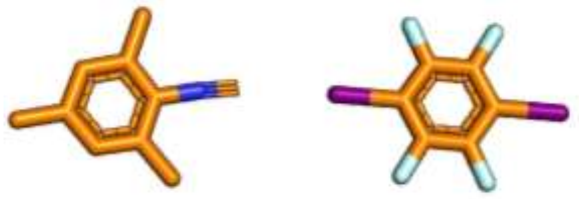
ZUTKUX01<br>CCDC 1981527	164.	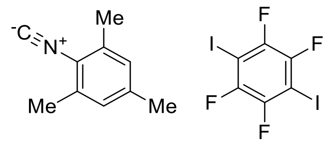	3.2	164.9	(142)	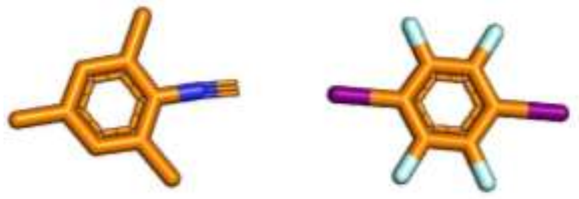
ZUTLAE<br>CCDC 1957701	165.	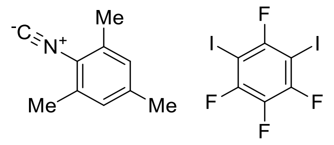	3.3	114.4	(142)	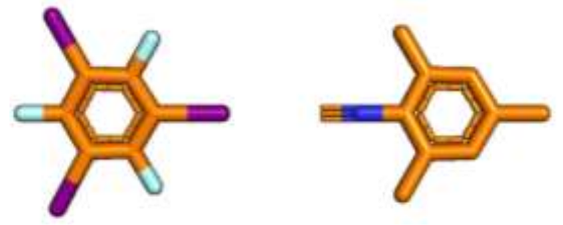
ZZZIZA01<br>CCDC 1319012	166.	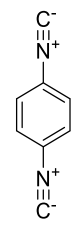	2.8	142.4	(143)	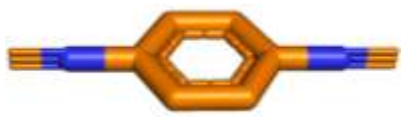
BIDJAD<br>CCDC 2233166	167.	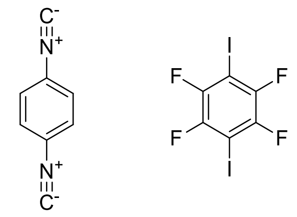	3.7<br>2.9	161.7<br>126.3	(218)	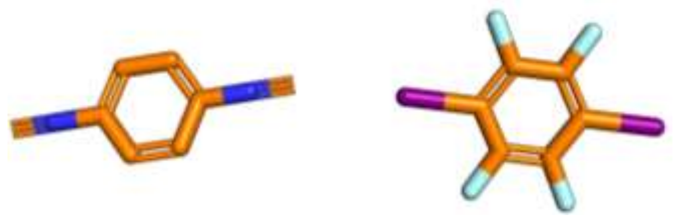

BIDJEH<br>CCDC 2233161	168.	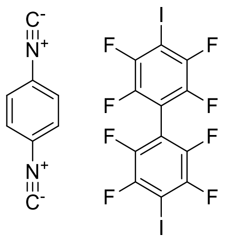	2.7<br>3.1	131.2<br>159.6	(218)	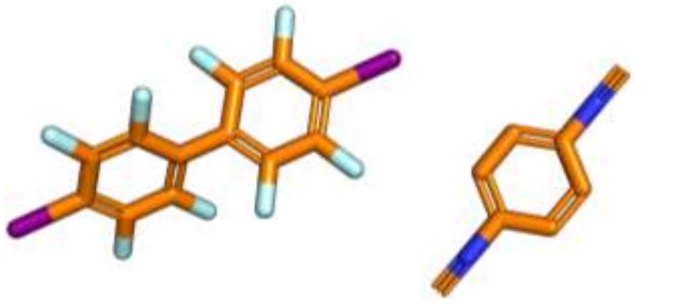
BIDJIL<br>CCDC 2233162	169.	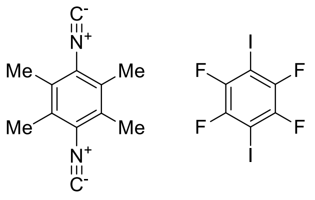	3.0	162.7	(218)	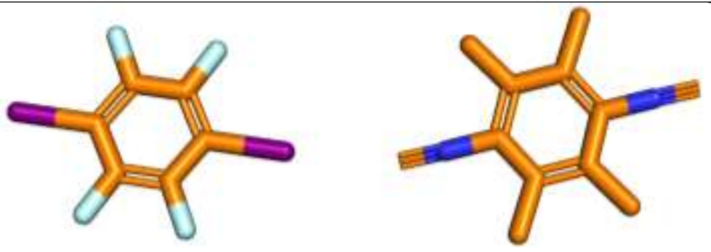
BIDJOR<br>CCDC 2233164	170.	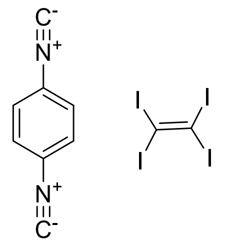	3.1<br>3.2<br>2.6<br>2.8	141.0<br>164.6<br>128.0<br>124.4	(218)	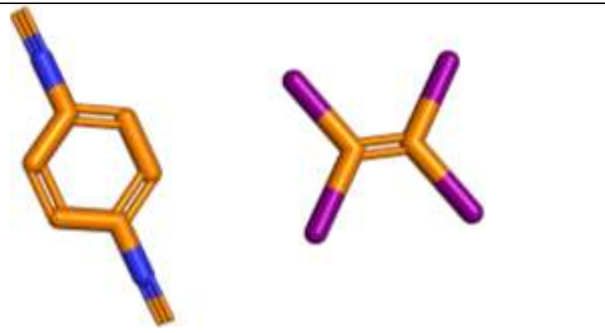

B) Organic isocyanides that do not form intermolecular interactions

CCDC code	no	Ref
BZICPC<br>CCDC 1118264	1.	(219)
CAXROL<br>CCDC 1052082	2.	(144)
DICAMP<br>CCDC 1140217	3.	(220)
ETAGOW<br>CCDC 798180	4.	(145)
FIXBEW<br>CCDC 1587294	5.	(146)
FIXXOZ<br>CCDC 1157542	6.	(147)
GAXMIB<br>CCDC 1164552	7.	(148)
HEZTOV<br>CCDC 1175720	8.	(229)
ICADOC	9.	(149)

CCDC 1179414		
ICOHAK<br>CCDC 607066	10.	(221)
ICTMBU<br>CCDC 1179605	11.	(150)
JABKEF<br>CCDC 1064132	12.	(151)
KELMAS<br>CCDC 1525810	13.	(152)
KIBHAF<br>CCDC 623050	14.	(62)
MADJUY<br>CCDC 756982	15.	(74)
MEFTOJ<br>CCDC 1544053	16.	(154)
ODEQIY<br>CCDC 637825	17.	(154154)
OTONOB<br>CCDC 835612	18.	(222)
OTONOB01<br>CCDC 2080563	19.	(223)
PUYVAG<br>CCDC 189380	20.	(155)
SUVCUI<br>CCDC 760015	21.	(156)
SUVJAW<br>CCDC 1421784	22.	(157)
TACLIS<br>CCDC 1265111	23.	(158)
TRIVIR<br>CCDC 1275716	24.	(228)
WAYXUR<br>CCDC 867306	25.	(159)
YIZNIF<br>CCDC 663020	26.	(224)
YONBEJ<br>CCDC 694486	27.	(160)
YUNMOJ<br>CCDC 1307095	28.	(225)
ZOUWIV<br>CCDC 1056659	29.	(161)
ZZZIZA<br>CCDC 1319011	30.	(226)
ZZZIZA02<br>CCDC 1319013	31.	(227)

c) Organometallic isocyanides forming interactions

CCDC code	No	Ref
AFIVAP<br>CCDC 884016	1.	(163)
ASAZAW<br>CCDC 797232	2.	(164)
BUPHOJ<br>CCDC 1116956	3.	(166)
CEVNOK<br>CCDC 2208757	4.	(167)
CEVPAY<br>CCDC 2208759	5.	(167)
DAPPEQ<br>CCDC 212022	6.	(231)
DUZFAH<br>CCDC 1411394	7.	(168)
DUZZEE<br>CCDC 790510	8.	(169)
EYOTAO<br>CCDC 816520	9.	(170)
EZATEG<br>CCDC 1487253	10.	(171)
FEJDOO<br>CCDC 246240	11.	(172)
FIRVAG<br>CCDC 1889062	12.	(173)
HITXEO<br>CCDC 676435	13.	(174)
HODKET<br>CCDC 1894953	14.	(176)
IFEZID<br>CCDC 602828	15.	(177)
IXAYOX<br>CCDC 1509471	16.	(178)
IXODUU<br>CCDC 144108	17.	(179)
JAMCAC	18.	(180)

CCDC 237096		
JAWNOL<br>CCDC 226896	19.	(181)
JOGDIS<br>CCDC 1188223	20.	(182)
KAWLEC<br>CCDC 1558580	21.	(183)
KAWMUT<br>CCDC 1558588	22.	(183)
KIWXUL<br>CCDC 958991	23.	(184)
KIXHEH<br>CCDC 1448880	24.	(185)
KORGED<br>CCDC 1199430	25.	(186)
LULDIG<br>CCDC 728257	26.	(187)
MATREH<br>CCDC 1546138	27.	(188)
MEBCUU<br>CCDC 1567184	28.	(189)
MEBDAB<br>CCDC 1567192	29.	(189)
MISNUA<br>CCDC 1508503	30.	(190)
MITQUC<br>CCDC 603239	31.	(191)
MOPMEL<br>CCDC 1018993	32.	(192)
NANYAG<br>CCDC 2096548	33.	(193)
NEBQIU<br>CCDC 122005	34.	(194)
QEGPEA<br>CCDC 1547794	35.	(198)
QUKFAG<br>CCDC 1893855	36.	(199)
REDQAT	37.	(200)

CCDC 283045		
SANQIL<br>CCDC 2106562	38.	(201)
TIFWIP<br>CCDC 657589	39.	(203)
UNACOC<br>CCDC 796944	40.	(204)
VEZJOD<br>CCDC 2207618	41.	(230)
WASDAW<br>CCDC 236440	42.	(207)
WETKAK<br>CCDC 1814535	43.	(208)
XAKBOB<br>CCDC 209332	44.	(209)
XEDREE<br>CCDC 601009	45.	(210)
XEMBUN<br>CCDC 619722	46.	(211)
XIJJOQ<br>CCDC 603435	47.	(212)
XIWJUJ<br>CCDC 642174	48.	(213)
YIYDOB<br>CCDC 949021	49.	(214)
YUZYID<br>CCDC 1436187	50.	(215)
ZEMRIS<br>CCDC 1311036	51.	(181)
ZURKOM<br>CCDC 1316949	52.	(217)

D) Organometallic isocyanides not forming interactions

**CCDC code**	**No**	**Ref**
ACIHIE<br>CCDC 271233	1.	(162)

BESHAK CCDC 916808	2.	(165)
CEXVAE CCDC 924957	3.	(232)
EZAVAE CCDC 1478975	4.	(233)
EZEJEA CCDC 1478977	5.	(233)
EZEJIE CCDC 1478978	6.	(233)
HUWVAZ CCDC 2006505	7.	(234)
HITXAK CCDC 676434	8.	(174)
HIXMOQ CCDC 116271	9.	(175)
LIYBIH CCDC 1883485	10.	(235)
NEBQEQ CCDC 122004	11.	(236)
NOTHUC CCDC 1942973	12.	(196)
OGOWEP CCDC 1909950	13.	(197)
POXNIB CCDC 1055059	14.	(237)
QURWEI CCDC 2000158	15.	(238)
QURWIM CCDC 2000159	16.	(238)
SOQGEM CCDC 1004609	17.	(202)
TATVIN CCDC 829997	18.	(239)
VACPIZ CCDC 188214	19.	(205)
VEBTAZ CCDC 874092	20.	(206)
VEGWUD CCDC 2039071	21.	(240)
XYICP CCDC 1298188	22.	(241)
ZADGUK CCDC 1994821	23.	(242)

3) Data mining in crystal structure databases

In this investigation, a thorough examination of isocyanide interactions within the Cambridge Structural Database (CSD) was carried out using ConQuest^{TM} 2023.2.0 (Build 382238). Following that, visual inspection of the crystal structures was conducted using Mercury^{TM} 2023.2.0 (Build 382238). As a result, an initial set of 173 crystal structures displaying isocyanide interactions was obtained, which was subsequently reduced to 170 structures after exclusion of three entries coordinating metal atoms (namely, 'ACIHIE', 'BESHAK', and 'SOQGEM'). To identify relevant isocyanide substructures, a substructure search was carried out through the CSD Python API modules ccdc.io and ccdc.search, matching the SMART string "*N#C". Data processing involved the utilization of Python libraries Pandas and NumPy whereas Seaborn and Matplotlib libraries were employed for graphical representation of the data[244,245,246,247]. Kernel density estimation states were computed using the stats sub-package within the scipy statistical module[248]. The 3D molecular structures were rendered using Pymol (The PyMOL Molecular Graphics System, Version 2.5 Schrödinger, LLC.), while their 2D representations were depicted using ChemDraw® (PerkinElmer) 20.0.0.38. Metalloids (e.g, Boron and Silicon) entries containing structures were kept.

4). References

1. Hohlman, R. M.; Newmister, S. A.; Sanders, J. N.; Khatri, Y.; Li, S.; Keramati, N. R.; Lowell, A. N.; Houk, K. N.; Sherman, D. H. Structural Diversification of Hapalindole and Fischerindole Natural Products via Cascade Biocatalysis. *ACS Catalysis* **2021**, *11* (8), 4670–4681.
2. Clément, S.; Aly, S. M.; Husson, J.; Fortin, D.; Strohmann, C.; Knorr, M.; Guyard, L.; Abd-El-Aziz, A. S.; Harvey, P. D. A-frame-containing Organometallic Oligomers Constructed from Homo- and Heterobimetallic m(M-dppm) 2 M′ (M/M′ = PD, Pt) Building Blocks. *European Journal of Inorganic Chemistry* **2009**, *2009* (17), 2536–2546.
3. Takeda, N.; Kajiwara, T.; Suzuki, H.; Okazaki, R.; Tokitoh, N. Synthesis and Properties of the First Stable Silylene–Isocyanide Complexes. *Chemistry - A European Journal* **2003**, *9* (15), 3530–3543.
4. Shi, S.-J.; Lv, X.-X.; Zhao, M.; Ma, J.-P.; Guo, D.-S. Synthesis, Characterization and Intermolecular Interactions in Crystals of Two P-Tert-Butylthiacalix[4]Arene Diisocyanide and Diamine Derivatives. *Journal of Molecular Structure* **2017**, *1127*, 81–87.
5. Di Blasio, B.; Fattorusso, E.; Magno, S.; Mayol, L.; Pedone, C.; Santacroce, C.; Sica, D. Axisonitrile-3, Axisothiocyanate-3 and Axamide-3. Sesquiterpenes with a Novel Spiro[4,5]Decane Skeleton from the Sponge Axinella Cannabina. *Tetrahedron* **1976**, *32* (4), 473–478.
6. Britton D.; Gleason W.B.; Glick M.; Crystal Structure Communications, 1981, 10, 1497
7. D.Britton; J.Konnert; S.Lam. *Crystal Structure Communications,* **1978**.
8. John, D. I.; Tyrrell, N. D.; Thomas, E. J.; Bentely, P. H.; Williams, D. J. Reactions of Benzyl 6-Lsocyanopenicellanate with Thiocarbonyl Reagents. Novel Rearrangements to 2,3-Dihydrothiazolo [2,3-b][1,3]-Thiazin -5-Ones via Penam C(5)–C(6) Bond Cleavage. X-Ray Crystal Structure and Absolute Configuration of Two Rearrangement Products. *J. Chem. Soc., Chem. Commun.* **1982**, *0* (1), 76–78.
9. Neochoritis, C. G.; Ghonchepour, E.; Miraki, M. K.; Zarganes-Tzitzikas, T.; Kurpiewska, K.; Kalinowska-Tłuścik, J.; Dömling, A. Structure and Reactivity of Glycosyl Isocyanides. *European Journal of Organic Chemistry* **2018**, *2019* (1), 50–55.
10. Wright, J. J.; Cooper, A. B.; McPhail, A. T.; Merrill, Y.; Nagabhushan, T. L.; Puar, M. S. X-Ray Crystal Structure Determination and Synthesis of the New Isonitrile-Containing Antibiotics, Hazimycin Factors 5 and 6. *Journal of the Chemical Society, Chemical Communications* **1982**, No. 20, 1188.
11. Tashkandi, N. Y.; McOnie, S. L.; Bourque, J. L.; Reinhold, C. R.; Baines, K. M. The Diverse Reactivity of Disilenes toward Isocyanides. *Angewandte Chemie International Edition* **2019**, *58* (10), 3167–3172.
12. Chan, T.-L.; Mak, T. C. X-Ray Crystallographic Study of Guest–Molecule Orientations in the β-Hydroquinone Clathrates of Acetonitrile and Methyl Isocyanide. *J. Chem. Soc., Perkin Trans. 2* **1983**, No. 6, 777–781.
13. Wang, X.; Ye, W.; Kong, T.; Wang, C.; Ni, C.; Hu, J. Divergent S- and C-Difluoromethylation of 2-Substituted Benzothiazoles. *Organic Letters* **2021**, *23* (21), 8554–8558.
14. Chepyshev, S. V.; Lujan-Montelongo, J. A.; Chao, A.; Fleming, F. F. Alkenyl Isocyanide Conjugate Additions: A Rapid Route to γ-Carbolines. *Angewandte Chemie International Edition* **2017**, *56* (15), 4310–4313.
15. Noland, W. E.; Herzig, R. J.; Engwall, A. J.; Jensen, R. C.; Tritch, K. J. Crystal Structures of Methyl 3,5-Dibromo-4-Cyanobenzoate and Methyl 3,5-Dibromo-4-Isocyanobenzoate. *Acta Crystallographica Section E Crystallographic Communications* **2018**, *74* (3), 345–348.
16. Chang, C. W.; Patra, A.; Roll, D. M.; Scheuer, P. J.; Matsumoto, G. K.; Clardy, J. Kalihinol-A, a Highly Functionalized Diisocyano Diterpenoid Antibiotic from a Sponge. *Journal of the American Chemical Society* **1984**, *106* (16), 4644–4646.

17. Patra, A.; Chang, C. W.; Scheuer, P. J.; Van Duyne, G. D.; Matsumoto, G. K.; Clardy, J. An Unprecedented Triisocyano Diterpenoid Antibiotic from a Sponge. *Journal of the American Chemical Society* **1984**, *106* (25), 7981–7983.
18. Zhao, M.-X.; Zhu, H.-K.; Dai, T.-L.; Shi, M. Cinchona Alkaloid Squaramide-Catalyzed Asymmetric Michael Addition of α-Aryl Isocyanoacetates to β-Trifluoromethylated Enones and Its Applications in the Synthesis of Chiral β-Trifluoromethylated Pyrrolines. *The Journal of Organic Chemistry* **2015**, *80* (22), 11330–11338.
19. Agnew, D. W.; Gembicky, M.; Moore, C. E.; Rheingold, A. L.; Figueroa, J. S. Robust, Transformable, and Crystalline Single-Node Organometallic Networks Constructed from Ditopic *m*-Terphenyl Isocyanides. *Journal of the American Chemical Society* **2016**, *138* (46), 15138–15141.
20. Kazlauskas, R.; Murphy, P. T.; Wells, R. J.; Blount, J. F. New Diterpene Isocyanides from a Sponge. *Tetrahedron Letters* **1980**, *21* (3), 315–318.
21. Yamamoto, Y.; Hagiwara, T.; Yamazaki, H. Axially Dissymmetric 2,2′-Diisocyano-1,1′-Binaphthyl and Its Platinum Complexes. *Inorganica Chimica Acta* **1986**, *115* (2).
22. Zhang, Y.; Maverick, A. W. Preparation of an Isocyano-β-Diketone via Its Metal Complexes, by Use of Metal Ions as Protecting Groups. *Inorganic Chemistry* **2009**, *48* (22), 10512–10518.
23. Mathieson, T.; Schier, A.; Schmidbaur, H. Supramolecular Chemistry of Gold(I) Thiocyanate Complexes with Thiophene, Phosphine and Isocyanide Ligands, and the Structure of 2,6-Dimethylphenyl Isocyanide. *Journal of the Chemical Society, Dalton Transactions* **2001**, No. 8, 1196–1200.
24. Brennessel, W. W.; Kucera, B. E.; Young, V. G.; Ellis, J. E. Crystal Structures and Spectroscopic Characterization of *m*BR2(Cnxyl) *n* (*m* = Fe and Co, *n* = 4; *m* = Ni, *n* = 2; Xyl = 2,6-Dimethylphenyl), and of Formally Zero-Valent Iron as a Cocrystal of Fe(CNXyl)5 and Fe2(Cnxyl)9. *Acta Crystallographica Section C Structural Chemistry* **2019**, *75* (8), 1118–1127..
25. Zeller, M.; Hunter, A. D. *P*-Nitrophenyl Isocyanide. *Acta Crystallographica Section C Crystal Structure Communications* **2004**, *60* (6).
26. Britton, D.; Sowa, J. R.; Mann, K. R. *P*-Decylphenyl Isocyanide and *p*-Decylbenzonitrile: Isomorphous Isonitrile/Nitrile Isomers. *Acta Crystallographica Section C Crystal Structure Communications* **2004**, *60* (6).
27. Moore, R. E.; Cheuk, C.; Yang, X. Q.; Patterson, G. M.; Bonjouklian, R.; Smitka, T. A.; Mynderse, J. S.; Foster, R. S.; Jones, N. D.; Swartzendruber, J. K.; Deeter, J. B. Hapalindoles, Antibacterial and Antimycotic Alkaloids from the Cyanophyte Hapalosiphon Fontinalis. *The Journal of Organic Chemistry* **1987**, *52* (6), 1036–1043.
28. Li, X.; Zarganes-Tzitzikas, T.; Kurpiewska, K.; Dömling, A. Amenamevir by UGI-4CR. *Green Chemistry* **2023**, *25* (4), 1322–1325.
29. Molinski, T. F.; Faulkner, D. J.; Van Duyne, G. D.; Clardy, J. Three New Diterpene Isonitriles from a Palauan Sponge of the Genus Halichondria. *The Journal of Organic Chemistry* **1987**, *52* (15), 3334–3337.
30. Schwartz, R. E.; Hirsch, C. F.; Springer, J. P.; Pettibone, D. J.; Zink, D. L. Unusual Cyclopropane-Containing Hapalindolinones from a Cultured Cyanobacterium. *The Journal of Organic Chemistry* **1987**, *52* (16), 3704–3706.
31. M.Zeller; A.D.Hunter. *CSD Communication* **2004**.
32. Pink, M.; Britton, D.; Noland, W. E.; Pinnow, M. J. 2,4,6-Trichlorophenylisonitrile and 2,4,6-Trichlorobenzonitrile. *Acta Crystallographica Section C Crystal Structure Communications* **2000**, *56* (10), 1271–1273.
33. Zhao, M.-X.; Jing, L.; Zhou, H.; Shi, M. Cinchona Alkaloid Thiourea Mediated Asymmetric Mannich Reaction of Isocyanoacetates with Isatin-Derived Ketimines and Subsequent Cyclization: Enantioselective Synthesis of Spirooxindole Imidazolines. *RSC Advances* **2015**, *5* (92), 75648–75652.
34. Duewel, S.; Schmermund, L.; Faber, T.; Harms, K.; Srinivasan, V.; Meggers, E.; Hoebenreich, S. Directed Evolution of an Feii-Dependent Halogenase for Asymmetric C(SP3)–h Chlorination. *ACS Catalysis* **2019**, *10* (2), 1272–1277.
35. Tada, H.; Tozyo, T.; Shiro, M. A New Isocyanide from a Sponge. Is the Formamide a Natural Product? The Journal of Organic Chemistry 1988, 53 (14), 3366–3368.
36. Bardají, M.; Font-Bardia, M.; Gallen, A.; Garcia-Cirera, B.; Ferrer, M.; Martínez, M. Iron Complexes of Bridging AZO Ligands in Aqueous Solution: Changes in the Thermal Switching Mechanism on Coordination and Oxidation State of Metal Centres. *Dalton Transactions* **2023**, *52* (6), 1720–1730.
37. Naik, A.; Meina, L.; Zabel, M.; Reiser, O. Efficient Aerobic Wacker Oxidation of Styrenes Using Palladium Bis(Isonitrile) Catalysts. *Chemistry - A European Journal* **2010**, *16* (5), 1624–1628.
38. Agnew, D. W.; Gembicky, M.; Moore, C. E.; Rheingold, A. L.; Figueroa, J. S. Robust, Transformable, and Crystalline Single-Node Organometallic Networks Constructed from Ditopic *m*-Terphenyl Isocyanides. *Journal of the American Chemical Society* **2016**, *138* (46), 15138–15141.

39. Weener, J.-W.; Versleijen, J. P.; Meetsma, A.; ten Hoeve, W.; van Leusen, A. M. Cis- Andtrans-2-(Isocyanomethyl)-5,5-Dimethyl-2-Oxo-4-Phenyl-1,3,2-Dioxaphosphorinane – Synthesis and Structure of the First Chiral Isocyanomethylphosphonate Synthons. *European Journal of Organic Chemistry* **1998**, *1998* (8), 1511–1516.
40. Agnew, D. W.; Gembicky, M.; Moore, C. E.; Rheingold, A. L.; Figueroa, J. S. Robust, Transformable, and Crystalline Single-Node Organometallic Networks Constructed from Ditopic *m*-Terphenyl Isocyanides. *Journal of the American Chemical Society* **2016**, *138* (46), 15138–15141.
41. Rodríguez, J.; Nieto, R. M.; Hunter, L. M.; Diaz, M. C.; Crews, P.; Lobkovsky, E.; Clardy, J. Variation among Known Kalihinol and New Kalihinene Diterpenes from the Sponge Acanthella Cavernosa. *Tetrahedron* **1994**, *50* (38), 11079–11090.
42. D.Britton; J.Konnert; S.Lam. *Crystal Structure Communications* **1978**.
43. Kazlauskas, R.; Murphy, P. T.; Wells, R. J.; Blount, J. F. New Diterpene Isocyanides from a Sponge. *Tetrahedron Letters* **1980**, *21* (3), 315–318.
44. Zheng, Q.; Kurpiewska, K.; Dömling, A. S n Ar Isocyanide Diversification. *European Journal of Organic Chemistry* **2021**, *2022* (3).
45. Hagadone, M. R.; Burreson, B. J.; Scheuer, P. J.; Finer, J. S.; Clardy, J. Defense Allomones of the Nudibranchphyllidia Varicosa Lamarck 1801. *Helvetica Chimica Acta* **1979**, *62* (7), 2484–2494.
46. Zeller, M.; Hunter, A. D. 1,4-Diisocyano-2,5-Dimethylbenzene. *Acta Crystallographica Section E Structure Reports Online* **2003**, *59* (11).
47. Zeller, M.; Hunter, A. D.; Perrine, C. L. *P*-Diisocyanotetramethylbenzene. *Acta Crystallographica Section E Structure Reports Online* **2003**, *59* (11).
48. Balto, K. P.; Gembicky, M.; Rheingold, A. L.; Figueroa, J. S. Crystalline Hydrogen-Bonding Networks and Mixed-Metal Framework Materials Enabled by an Electronically Differentiated Heteroditopic Isocyanide/Carboxylate Linker Group. *Inorganic Chemistry* **2021**, *60* (16), 12545–12554.
49. Neochoritis, C. G.; Zarganes-Tzitzikas, T.; Stotani, S.; Dömling, A.; Herdtweck, E.; Khoury, K.; Dömling, A. Leuckart–Wallach Route toward Isocyanides and Some Applications. *ACS Combinatorial Science* **2015**, *17* (9), 493–499.
50. Kolb, J.; Beck, B.; Almstetter, M.; Heck, S.; Herdtweck, E.; Dömling, A. New Mcrs: The First 4-Component Reaction Leading to 2,4-Disubstituted Thiazoles. *Molecular Diversity* **2000**, *6* (3/4), 297–313.
51. Ji, X.; Cao, W.-G.; Zhao, G. Dual-Reagent Organophosphine Catalyzed Asymmetric Mannich Reactions of Isocyanoacetates with N -BOC-Aldimines. *Tetrahedron* **2017**, *73* (41), 5983–5992.
52. Wang, Z.; Li, Y.; Han, X.; Zhang, D.; Hou, H.; Xiao, L.; Li, G. Kalihiacyloxyamides A-h, α-Acyloxy Amide Substituted Kalihinane Diterpenes Isolated from the Sponge Acanthella Cavernosa Collected in the South China Sea. *Phytochemistry* **2023**, *206*, 113512.
53. Itoh, J.; Takeuchi, Y.; Gomi, S.; Inouye, S.; Mikawa, T.; Yoshikawa, N.; Ohkishi, H. MK4588, a New Antibiotic Related to Xanthocillin. *J. Antibiot. (Tokyo)* **1990**, *43* (5), 456–461.
54. Dyrbusch, M.; Egert, E. Structure of a Cinnamic Acid Derivative. *Acta Crystallographica Section C Crystal Structure Communications* **1992**, *48* (11), 2035–2036.
55. Khan, I.; Zhang, H.; Liu, W.; Zhang, L.; Peng, F.; Chen, Y.; Zhang, Q.; Zhang, G.; Zhang, W.; Zhang, C. Identification and Bioactivity Evaluation of Secondary Metabolites from Antarctic-Derived *Penicillium Chrysogenum* CCTCC M 2020019. *RSC Advances* **2020**, *10* (35), 20738–20744.
56. Andreini, M.; Chapellas, F.; Diab, S.; Pasturaud, K.; Piettre, S. R.; Legros, J.; Chataigner, I. Addition of 4-(Cyclohex-1-En-1-Yl)Morpholine on 3-Nitroindole: An Unprecedented Dearomatizing Process. *Organic & Biomolecular Chemistry* **2016**, *14* (10), 2833–2839.
57. Cornelissen, J. J.; Donners, J. J.; de Gelder, R.; Graswinckel, W. S.; Metselaar, G. A.; Rowan, A. E.; Sommerdijk, N. A.; Nolte, R. J. B-Helical Polymers from Isocyanopeptides. *Science* **2001**, *293* (5530), 676–680.
58. Li, G.-W.; Cao, J.-M.; Zong, W.; Hu, L.; Hu, M.-L.; Lei, X.; Sun, H.; Tan, R. X. Helical Polyisocyanopeptides as Lyotropic Liquid Crystals for Measuring Residual Dipolar Couplings. *Chemistry - A European Journal* **2017**, *23* (32), 7653–7656.
59. Wang, W.; Sun, X.; Qu, J.; Xie, X.; Qi, Z.-H.; Hong, D.; Jing, S.; Zheng, D.; Tian, Y.; Ma, H.; Yu, S.; Ma, J. Aggregation-Induced Visible Light Absorption Makes Reactant 1,2-Diisocyanoarenes Act as Photosensitizers in Double Radical Isocyanide Insertions. *Physical Chemistry Chemical Physics* **2017**, *19* (46), 31443–31451.
60. Ruiz, J.; Mateo, M. A. The First Synthesis of an Isocyanide-Functionalized Imidazolium Salt and Transition Metal Complexes Thereof. *Dalton Transactions* **2022**, *51* (35), 13199–13203.
61. Ibad, M. F.; Langer, P.; Reiß, F.; Schulz, A.; Villinger, A. Catalytic Trimerization of Bis-Silylated Diazomethane. *Journal of the American Chemical Society* **2012**, *134* (42), 17757–17768.
62. Maimone, T. J.; Ishihara, Y.; Baran, P. S. Scalable Total Syntheses of (−)-Hapalindole U and (+)-Ambiguine H. *Tetrahedron* **2015**, *71* (22), 3652–3665.

63. Baran, P. S.; Maimone, T. J.; Richter, J. M. Total Synthesis of Marine Natural Products without Using Protecting Groups. *Nature* **2007**, *446* (7134), 404–408.
64. Pronin, S. V.; Reiher, C. A.; Shenvi, R. A. Stereoinversion of Tertiary Alcohols to Tertiary-Alkyl Isonitriles and Amines. *Nature* **2013**, *501* (7466), 195–199.
65. Hahn, E. E.; Tamm, M. Chelate Complexes with TRIISOCYANIDE Ligands. *Angewandte Chemie International Edition in English* **1991**, *30* (2), 203–205..
66. Zhao, M.-X.; Ji, F.-H.; Wei, D.-K.; Shi, M. Chiral Squaramides Catalyzed Diastereo- and Enantioselective Michael Addition of α-Substituted Isocyanoacetates to N-Aryl Maleimides. *Tetrahedron* **2013**, *69* (50), 10763–10771.
67. Bai, J.-F.; Wang, L.-L.; Peng, L.; Guo, Y.-L.; Jia, L.-N.; Tian, F.; He, G.-Y.; Xu, X.-Y.; Wang, L.-X. Asymmetric Michael Addition of α-Substituted Isocyanoacetates with Maleimides Catalyzed by Chiral Tertiary Amine Thiourea. *The Journal of Organic Chemistry* **2012**, *77* (6), 2947–2953..
68. Smitka, T. A.; Bonjouklian, R.; Doolin, L.; Jones, N. D.; Deeter, J. B.; Yoshida, W. Y.; Prinsep, M. R.; Moore, R. E.; Patterson, G. M. Ambiguine Isonitriles, Fungicidal Hapalindole-Type Alkaloids from Three Genera of Blue-Green Algae Belonging to the Stigonemataceae. *The Journal of Organic Chemistry* **1992**, *57* (3), 857–861
69. Sharma, H. A.; Tanaka, J.; Higa, T.; Lithgow, A.; Bernardinelli, G.; Jefford, C. W. Two New Diterpene Isocyanides from a Sponge of the Family Adocidae. *Tetrahedron Letters* **1992**, *33* (12), 1593–1596.
70. Vriezema, D. M.; Kros, A.; de Gelder, R.; Cornelissen, J. J.; Rowan, A. E.; Nolte, R. J. Electroformed Giant Vesicles from Thiophene-Containing Rod−coil Diblock Copolymers. *Macromolecules* **2004**, *37* (12), 4736–4739.
71. Zheng, Q.; Kurpiewska, K.; Dömling, A. S n Ar Isocyanide Diversification. *European Journal of Organic Chemistry* **2021**, *2022* (3).
72. Britton, D. Crystal Structure of P-Isocyanoaniline. *J. Crystallogr. Spectrosc. Res.* **1993**, *23* (8), 689–690.
73. Bano, H.; Yousuf, S. Crystal Structure of*p*-Toluenesulfonylmethyl Isocyanide. *Acta Crystallographica Section E Crystallographic Communications* **2015**, *71* (6).
74. Ichikawa, Y.; Watanabe, H.; Kotsuki, H.; Nakano, K. Anomeric Effect of the Nitrogen Atom in the ISOCYANO and Urea Groups. *European Journal of Organic Chemistry* **2010**, *2010* (33), 6331–6337..
75. Buschmann, J.; Kleinhenz, S.; Lentz, D.; Luger, P.; Madappat, K. V.; Preugschat, D.; Thrasher, J. S. Crystal and Molecular Structures of Trifluoroacrylonitrile, F2CCF−CN, and Trifluorovinyl Isocyanide, f2CCF−NC, by Low-Temperature x-Ray Crystallography and Ab Initio Calculations. *Inorganic Chemistry* **2000**, *39* (13), 2807–2812
76. Paravidino, M.; Bon, R. S.; Scheffelaar, R.; Vugts, D. J.; Znabet, A.; Schmitz, R. F.; de Kanter, F. J.; Lutz, M.; Spek, A. L.; Groen, M. B.; Orru, R. V. Diastereoselective Multicomponent Synthesis of Dihydropyridones with an Isocyanide Functionality. *Organic Letters* **2006**, *8* (23), 5369–5372.
77. Noland, W. E.; Tritch, K. J. 2,6-Dibromo-4-Chlorophenyl Isocyanide. *IUCrData* **2018**, *3* (1)..
78. Noland, W. E.; Britton, D.; Sutton, G. K.; Schneerer, A. K.; Tritch, K. J. Crystal Structures of 2,4,6-Triiodobenzonitrile and 2,4,6-Triiodophenyl Isocyanide. *Acta Crystallographica Section E Crystallographic Communications* **2018**, *74* (2), 98–102.
79. Rukiah, M.; Al-Ktaifani, M. Powder X-Ray Investigation of 4,4′-Diisocyano-3,3′-Dimethylbiphenyl. *Acta Crystallographica Section E Structure Reports Online* **2013**, *69* (3).
80. Schwarz, O.; Brun, R.; Bats, J. W.; Schmalz, H.-G. Synthesis and Biological Evaluation of New Antimalarial ISONITRILES Related to Marine Diterpenoids. *Tetrahedron Letters* **2002**, *43* (6), 1009–1013.
81. Dong, J.; Wang, X.; Shi, H.; Wang, L.; Hu, Z.; Li, Y.; Xu, X. Tandem Cyclization−annulation of α-Acidic Isocyanides with 2-Methyleneaminochalcones: Synthesis of Pyrrolo[2,3-*c*]Quinoline Derivatives. *Advanced Synthesis & Catalysis* **2018**, *361* (4), 863–867.
82. Toriyama, M.; Maher, T. R.; Holovics, T. C.; Vanka, K.; Day, V. W.; Berrie, C. L.; Thompson, W. H.; Barybin, M. V. Multipoint Anchoring of the [2.2.2.2]Metacyclophane Motif to a Gold Surface via Self-Assembly: Coordination Chemistry of a Cyclic Tetraisocyanide Revisited. *Inorganic Chemistry* **2008**, *47* (8), 3284–3291.
83. Fernandes, M. A.; Layh, M.; Omondi, B. 2,4,6-Trimethylphenyl Isocyanide. *Acta Crystallographica Section C Crystal Structure Communications* **2002**, *58* (7).
84. Buschmann, J.; Kleinhenz, S.; Lentz, D.; Luger, P.; Madappat, K. V.; Preugschat, D.; Thrasher, J. S. Crystal and Molecular Structures of Trifluoroacrylonitrile, F2CCF−CN, and Trifluorovinyl Isocyanide, f2CCF−NC, by Low-Temperature x-Ray Crystallography and Ab Initio Calculations. *Inorganic Chemistry* **2000**, *39* (13), 2807–2812.
85. Butera, R.; Shrinidhi, A.; Kurpiewska, K.; Kalinowska-Tłuścik, J.; Dömling, A. Fourfold Symmetric MCR's *via* the Tetraisocyanide 1,3-Diisocyano-2,2-Bis(Isocyanomethyl)Propane. *Chemical Communications* **2020**, *56* (73), 10662–10665.

86. Buschmann, J.; Bartolmäs, T.; Lentz, D.; Luger, P.; Neubert, I.; Röttger, M. Synthesis, Structure, and Coordination Chemistry of Diisocyanomethane. *Angewandte Chemie International Edition in English* **1997**, *36* (21), 2372–2374
87. Koritsánszky, T.; Buschmann, J.; Lentz, D.; Luger, P.; Perpetuo, G.; Röttger, M. Topological Analysis of the Experimental Electron Density of Diisocyanomethane at 115 K. *Chemistry - A European Journal* **1999**, *5* (11), 3413–3420.
88. Tan, W. S.; Prabhakar, Ch.; Liu, Y.-H.; Peng, S.-M.; Yang, J.-S. Effects of Iptycene Scaffolds on the Photoluminescence of N, N-Dimethylaminobenzonitrile and Its Analogues. *Photochemical & Photobiological Sciences* **2014**, *13* (2), 211–223.
89. Ditri, T. B.; Fox, B. J.; Moore, C. E.; Rheingold, A. L.; Figueroa, J. S. Effective Control of Ligation and Geometric Isomerism: Direct Comparison of Steric Properties Associated with Bis-Mesityl and Bis-Diisopropylphenyl *m*-Terphenyl Isocyanides. *Inorganic Chemistry* **2009**, *48* (17), 8362–8375.
90. Scheffelaar, R.; Paravidino, M.; Znabet, A.; Schmitz, R. F.; de Kanter, F. J.; Lutz, M.; Spek, A. L.; Guerra, C. F.; Bickelhaupt, F. M.; Groen, M. B.; Ruijter, E.; Orru, R. V. Scope and Limitations of an Efficient Four-Component Reaction for Dihydropyridin-2-Ones. *The Journal of Organic Chemistry* **2010**, *75* (5), 1723–1732.
91. Xu, X.; Liu, Q.; Zhang, D.; Tan, J. Direct Stereoselective Synthesis of 1-Amino-2,5-Diarylcyclohexanecarboxylic Acid Derivatives Based on a [5+1] Annulation of Divinyl Ketone and Isocyanoacetate. *Synlett* **2010**, *2010* (06), 917–920.
92. Zhang, D.; Hao, L.; Li, J. Ethyl 2,6-Bis(4-Bromophenyl)-1-Isocyano-4-Oxocyclohexanecarboxylate. *Acta Crystallographica Section E Structure Reports Online* **2014**, *70* (8).
93. Maher, T. R.; Spaeth, A. D.; Neal, B. M.; Berrie, C. L.; Thompson, W. H.; Day, V. W.; Barybin, M. V. Linear 6,6′-BIAZULENYL Framework Featuring Isocyanide Termini: Synthesis, Structure, Redox Behavior, Complexation, and Self-Assembly on Au(111). *Journal of the American Chemical Society* **2010**, *132* (45), 15924–15926.
94. Surmiak, E.; Neochoritis, C. G.; Musielak, B.; Twarda-Clapa, A.; Kurpiewska, K.; Dubin, G.; Camacho, C.; Holak, T. A.; Dömling, A. Rational Design and Synthesis of 1,5-Disubstituted Tetrazoles as Potent Inhibitors of the MDM2-P53 Interaction. *European Journal of Medicinal Chemistry* **2017**, *126*, 384–407
95. Aakeröy, C. B.; Hurley, E. P.; Desper, J. Modulating Supramolecular Reactivity Using Covalent “Switches” on a Pyrazole Platform. *Crystal Growth & Design* **2012**, *12* (11), 5806–5814.
96. Mayer, A. M. S.; Avilés, E.; Rodríguez, A. D. Marine Sponge Hymeniacidon Sp. Amphilectane Metabolites Potently Inhibit Rat Brain Microglia Thromboxane B2 Generation. *Bioorganic & Medicinal Chemistry* **2012**, *20* (1), 279–282.
97. Arroyave, A.; Gembicky, M.; Rheingold, A. L.; Figueroa, J. S. Aqueous Stability and Ligand Substitution of a Layered Cu(i)/Isocyanide-Based Organometallic Network Material with a Well-Defined Channel Structure. *Inorganic Chemistry* **2020**, *59* (17), 11868–11878.
98. Joost, M.; Nava, M.; Transue, W. J.; Cummins, C. C. An Exploding N-Isocyanide Reagent Formally Composed of Anthracene, Dinitrogen and a Carbon Atom. *Chem. Commun.* **2017**, *53* (83), 11500–11503.
99. Bai, J.-F.; Wang, L.-L.; Peng, L.; Guo, Y.-L.; Jia, L.-N.; Tian, F.; He, G.-Y.; Xu, X.-Y.; Wang, L.-X. Asymmetric Michael Addition of α-Substituted Isocyanoacetates with Maleimides Catalyzed by Chiral Tertiary Amine Thiourea. *The Journal of Organic Chemistry* **2012**, *77* (6), 2947–2953.
100. Ekkehardt Hahn, F.; Tamm, M.; Imhof, L.; Lügger, T. Preparation and Crystal Structures of a Bidentate Isocyanide and Its Tetracarbonylchromium Complex. *Journal of Organometallic Chemistry* **1996**, *526* (1), 149–155.
101. Marsh, R. E.; Kapon, M.; Hu, S.; Herbstein, F. H. Some 60 New Space-Group Corrections. *Acta Crystallographica Section B Structural Science* **2001**, *58* (1), 62–77.
102. Janssen, G. V.; Vicente-García, E.; Vogel, W.; Slootweg, J. C.; Ruijter, E.; Lammertsma, K.; Orru, R. V. Stereoselective Synthesis of β-Sulfinylamino Isocyanides and 2-Imidazolines. *European Journal of Organic Chemistry* **2014**, *2014* (18), 3762–3766.
103. Peters, K.; Peters, E.-M.; von Schnering, H. G.; Pakusch, J.; Beckhaus, H.-D.; Rüchardt, C. Crystal Structure of 2,3-Diisocyano-2,3-Diphenylbutane, [H3CC(NC)(C6H5)]2. *Zeitschrift für Kristallographie - Crystalline Materials* **1997**, *212* (1), 75.
104. Lu, Z.; Yang, M.; Chen, P.; Xiong, X.; Li, A. Total Synthesis of Hapalindole-Type Natural Products. *Angewandte Chemie International Edition* **2014**, *53* (50), 13840–13844.
105. F.E.Hahn; T.Lugger. Crystal Structure of 2-Benzyloxyphenylisocyanide, C14H11No. *Zeitschrift für Kristallographie - Crystalline Materials* **1997**, *212* (2), 165–165..
106. Xu, Y.; Li, N.; Jiao, W.-H.; Wang, R.-P.; Peng, Y.; Qi, S.-H.; Song, S.-J.; Chen, W.-S.; Lin, H.-W. Antifouling and Cytotoxic Constituents from the South China Sea Sponge Acanthella Cavernosa. *Tetrahedron* **2012**, *68* (13), 2876–2883.
107. Stolzenberg, H.; Weinberger, B.; Fehlhammer, W. P.; Pühlhofer, F. G.; Weiss, R. Free and Metal-coordinated (*n* -iso-cyanimino)Triphenylphosphorane: X-ray Structures and Selected Reactions. *European Journal of Inorganic Chemistry* **2005**, *2005* (21), 4263–4271.

108. Brough, P.; Pécaut, J.; Rassat, A.; Rey, P. Pyrimidinyl Nitronyl Nitroxides. *Chemistry - A European Journal* **2006**, *12* (19), 5134–5141.
109. F.E.Hahn; T.Lugger; P.Hein. *Zeitschrift fur Kristallographie - New Crystal Structures* **1998**.
110. Meetsma, A.; Stoelwinder, J.; van Leusen, A. M. Structure of an (e)-17-(Diethylphosphonoisocyanomethylene) Steroid. *Acta Crystallographica Section C Crystal Structure Communications* **1992**, *48* (6), 1138–1141
111. Schwartz, E.; Lim, E.; Gowda, C. M.; Liscio, A.; Fenwick, O.; Tu, G.; Palermo, V.; de Gelder, R.; Cornelissen, J. J.; Van Eck, E. R.; Kentgens, A. P.; Cacialli, F.; Nolte, R. J.; Samorì, P.; Huck, W. T.; Rowan, A. E. Synthesis, Characterization, and Surface Initiated Polymerization of Carbazole Functionalized Isocyanides. *Chemistry of Materials* **2010**, *22* (8), 2597–2607.
112. Perpétuo, G. J.; Buschmann, J.; Luger, P.; Lentz, D.; Dreissig, D. Low-Temperature Crystallization and Structure Determination of *n*-(Trifluoromethyl)Formamide, *n*-(2,2,2-Trifluoroethyl)Formamide and 2,2,2-Trifluoroethyl Isocyanide. *Acta Crystallographica Section B Structural Science* **1999**, *55* (1), 70–77.
113. Brady, S. F.; Clardy, J. Cloning and Heterologous Expression of Isocyanide Biosynthetic Genes from Environmental DNA. *Angewandte Chemie International Edition* **2005**, *44* (43), 7063–7065..
114. Stolzenberg, H.; Weinberger, B.; Fehlhammer, W. P.; Pühlhofer, F. G.; Weiss, R. Free and Metal-coordinated (*n* -iso-cyanimino)Triphenylphosphorane: X-ray Structures and Selected Reactions. *European Journal of Inorganic Chemistry* **2005**, *2005* (21), 4263–4271.
115. Britton, D.; Noland, W. E.; Tritch, K. J. Two New Polytypes of 2,4,6-Tribromobenzonitrile. *Acta Crystallographica Section E Crystallographic Communications* **2016**, *72* (2), 178–183.
116. Linden, A.; König, G. M.; Wright, A. D. Four Diterpene Isonitriles from the Sponge Cymbastela Hooperi. *Acta Crystallographica Section C Crystal Structure Communications* **1996**, *52* (10), 2601–2607.
117. Ollis, W. D.; Rey, M.; Godtfredsen, W. O.; Rastrup-Andersen, N.; Vangedal, S.; King, T. J. The Constitution of the Antibiotic Trichoviridin. *Tetrahedron* **1980**, *36* (4), 515–520.
118. Volbach, L.; Struch, N.; Bohle, F.; Topić, F.; Schnakenburg, G.; Schneider, A.; Rissanen, K.; Grimme, S.; Lützen, A. Influencing the Self-sorting Behavior of [2.2]Paracyclophane-based Ligands by Introducing Isostructural Binding Motifs. *Chemistry – A European Journal* **2020**, *26* (15), 3335–3347.
119. Fang, H.-P.; Fu, C.-C.; Tai, C.-K.; Chang, K.-H.; Yang, R.-H.; Wu, M.-J.; Chen, H.-C.; Li, C.-J.; Huang, S.-Q.; Lien, W.-H.; Chen, C.-H.; Hsieh, C.-H.; Wang, B.-C.; Cheung, S.-F.; Pan, P.-S. Synthesis and Stability Study of Isocyano Aryl Boronate Esters and Their Synthetic Applications. *RSC Advances* **2016**, *6* (36), 30362–30371
120. Cueny, E. S.; Johnson, H. C.; Anding, B. J.; Landis, C. R. Mechanistic Studies of Hafnium-Pyridyl Amido-Catalyzed 1-Octene Polymerization and Chain Transfer Using Quench-Labeling Methods. *Journal of the American Chemical Society* **2017**, *139* (34), 11903–11912.
121. Altundas, B.; Alwedi, E.; Song, Z.; Gogoi, A. R.; Dykstra, R.; Gutierrez, O.; Fleming, F. F. Dearomatization of Aromatic Asmic Iso-cyanides to Complex Cyclohexadienes. *Nature Communications* **2022**, *13* (1), 6444.
122. Schraff, S.; Trampert, J.; Orthaber, A.; Pammer, F. Electronic Properties and Solid-State Packing of Isocyanofulvenes and Their Gold(I) Chloride Complexes. *Inorganic Chemistry* **2020**, *59* (23), 17171–17183.
123. Mo, S.; Krunic, A.; Santarsiero, B. D.; Franzblau, S. G.; Orjala, J. Hapalindole-Related Alkaloids from the Cultured Cyanobacterium Fischerella Ambigua. *Phytochemistry* **2010**, *71* (17–18), 2116–2123.
124. Skodje, K. M.; Hinkle, L. M.; Miranda, M. O.; Mann, K. R.; Janzen, D. E. Nonisomorphous X-Ray Structures of Tritylnitrile and Tritylisonitrile. *Journal of Chemical Crystallography* **2012**, *42* (9), 972–980.
125. Ciavatta, M. L.; Gavagnin, M.; Manzo, E.; Puliti, R.; Mattia, C. A.; Mazzarella, L.; Cimino, G.; Simpson, J. S.; Garson, M. J. Structural and Stereochemical Revision of Isocyanide and Isothiocyanate Amphilectenes from the Caribbean Marine Sponge Cribochalina SP.. *Tet-rahedron* **2005**, *61* (33), 8049–8053.
126. Burnham, L. E.; Gano, K. J.; Young, A. M.; Risley, J. M.; Jones, D. S. *N*-(4-Isocyanophenyl)Succinamic Acid. *Acta Crystallographica Section E Structure Reports Online* **2012**, *68* (7).
127. Holovics, T. C.; Robinson, R. E.; Weintrob, E. C.; Toriyama, M.; Lushington, G. H.; Barybin, M. V. The 2,6-Diisocyanoazulene Motif: Synthesis and Efficient Mono- and Heterobimetallic Complexation with Controlled Orientation of the Azulenic Dipole. *Journal of the American Chemical Society* **2006**, *128* (7), 2300–2309..
128. Mikherdov, A. S.; Popov, R. A.; Smirnov, A. S.; Eliseeva, A. A.; Novikov, A. S.; Boyarskiy, V. P.; Gomila, R. M.; Frontera, A.; Kukushkin, V. Yu.; Bokach, N. A. Isocyanide and Cyanide Entities Form Isostructural Halogen Bond-Based Supramolecular Networks Featuring Five-Center Tetrafurcated Halogen⋯c/N Bonding. *Crystal Growth & Design* **2022**, *22* (10), 6079–6087.

129. Mueller, L. G.; Keller, T. M.; Fleming, F. F. One-Pot Syntheses of Substituted Oxazoles and Imidazoles from the Isocyanide Asmic. *The Journal of Organic Chemistry* **2023**, *88* (2), 909–916
130. Yamamoto, Y.; Nakamura, H.; Ma, J.-F. Preparation and Characterization of Ruthenium(Ii), Rhodium(Iii) and Iridium(Iii) Complexes of Isocyanide Bearing the AZO Group. *Journal of Organometallic Chemistry* **2001**, *640* (1–2), 10–20.
131. Li, D.; Wang, L.; Zhu, H.; Bai, L.; Yang, Y.; Zhang, M.; Yang, D.; Wang, R. Catalytic Asymmetric Reactions of α-Isocyanoacetates and *Meso*-Aziridines Mediated by an in-Situ-Generated Magnesium Catalytic Method. *Organic Letters* **2019**, *21* (12), 4717–4720..
132. Zhang, D.; Yang, P.; Liu, W.; Li, J. Ethyl 2,6-Bis(4-Chlorophenyl)-1-Isocyano-4-Oxocyclohexanecarboxylate. *Acta Crystallographica Section E Structure Reports Online* **2014**, *70* (7).
133. Khamphaijun, K.; Namnouad, P.; Docker, A.; Ruengsuk, A.; Tantirungrotechai, J.; Díaz-Torres, R.; Harding, D. J.; Bunchuay, T. Neutral Isocyanide-Templated Assembly of Pillar[5]Arene [2] and [3]Pseudorotaxanes. *Chemical Communications* **2022**, *58* (52), 7253–7256.
134. Brady, S. F.; Bauer, J. D.; Clarke-Pearson, M. F.; Daniels, R. Natural Products from *Isna*-Containing Biosynthetic Gene Clusters Recovered from the Genomes of Cultured and Uncultured Bacteria. *Journal of the American Chemical Society* **2007**, *129* (40), 12102–12103.
135. Buschmann, J.; Lentz, D.; Luger, P.; Perpetuo, G.; Scharn, D.; Willemsen, S. Synthese, Strukturuntersuchung Und Ligandeneigenschaften von Isocyanacetonitril. *Angewandte Chemie* **1995**, *107* (8), 988–990.
136. Buschmann, J.; Lentz, D.; Luger, P.; Röttger, M.; Perpetuo, G.; Scharn, D.; Willemsen, S. Synthese, Strukturuntersuchung Und Koordinationschemie von Isocyanacetonitril. *Zeitschrift für anorganische und allgemeine Chemie* **2000**, *626* (10), 2107–2116.
137. Homann, V. V.; Sandy, M.; Tincu, J. A.; Templeton, A. S.; Tebo, B. M.; Butler, A. Loihichelins A−F, a Suite of Amphiphilic Siderophores Produced by the Marine Bacterium *Halomonas* Lob-5. *Journal of Natural Products* **2009**, *72* (5), 884–888.
138. Greb, L.; Eichhöfer, A.; Lehn, J. Synthetic Molecular Motors: Thermal n Inversion and Directional Photoinduced CN Bond Rotation of Camphorquinone Imines. *Angewandte Chemie International Edition* **2015**, *54* (48), 14345–14348..
139. Das, A. K.; Mazumdar, S. K. 3’-Isocyano-2’,3’-Dideoxyuridine (Ncddurd): A Nucleoside Analogue. *Acta Crystallographica Section C Crystal Structure Communications* **1995**, *51* (8), 1652–1654.
140. Yu, K.-M.; Zhu, H.-K.; Zhao, X.-L.; Shi, M.; Zhao, M.-X. Organocatalyzed Asymmetric Tandem Conjugate Addition–Protonation of Isocyanoacetates to 2-Chloroacrylonitrile. *Organic & Biomolecular Chemistry* **2019**, *17* (3), 639–645.
141. A. E. Carpenter, C. C. Mokhtarzadeh, D. S. Ripatti, I. Havrylyuk, R. Kamezawa, C. E. Moore, Arnold. L. Rheingold, J. S. Figueroa, *Inorganic Chemistry* **2015**, *54*, 2936–2944.
142. A. S. Mikherdov, A. S. Novikov, V. P. Boyarskiy, V. Yu. Kukushkin, The halogen bond with isocyano carbon reduces isocyanide odor, *Nature Communications* **2020**, *11*,
143. M. Colapietro, A. Domenicano, G. Portalone, I. Torrini, I. Hargittai, G. Schultz, *Journal of Molecular Structure* **1984**, *125*, 19–32.
144. S. V. Chepyshev, J. A. Lujan-Montelongo, A. Chao, F. F. Fleming, Alkenyl Isocyanide Conjugate Additions: A Rapid Route to γ-Carbolines, *Angewandte Chemie International Edition* **2017**, *56*, 4310–4313.
145. Y. Li, X. Xu, J. Tan, C. Xia, D. Zhang, Q. Liu, Double Isocyanide Cyclization: A Synthetic Strategy for Two-Carbon-Tethered Pyrrole/Oxazole Pairs, *Journal of the American Chemical Society* **2011**, *133*, 1775–1777.
146. M.-X. Zhao, G.-Y. Zhu, X.-L. Zhao, M. Shi, Cinchona alkaloid derived squaramide catalyzed diastereo- and enantioselective Michael addition of isocyanoacetates to 2-enoylpyridines, *Tetrahedron* **2019**, *75*, 1171–1179.
147. T. F. Molinski, D. J. Faulkner, G. D. Van Duyne, J. Clardy, Three new diterpene isonitriles from a Palauan sponge of the genus Halichondria, *The Journal of Organic Chemistry* **1987**, *52*, 3334–3337.
148. C. J. Fookes, M. J. Garson, J. K. MacLeod, B. W. Skelton, A. H. White, Biosynthesis of diisocyanoadociane, a novel diterpene from the marine sponge Amphimedon sp. Crystal structure of a monoamide derivative, *Journal of the Chemical Society, Perkin Transactions 1* **1988**, 1003.
149. J. T. Baker, R. J. Wells, W. E. Oberhaensli, G. B. Hawes, A new diisocyanide of novel ring structure from a sponge, *Journal of the American Chemical Society* **1976**, *98*, 4010–4012.
150. A.L.Spek, *Crystal Structure Communications* **1979**.
151. M. Knorn, T. Rawner, R. Czerwieniec, O. Reiser, [Copper(phenanthroline)(bisisonitrile)]+-Complexes for the Visible-Light-Mediated Atom Transfer Radical Addition and Allylation Reactions , *ACS Catalysis* **2015**, *5*, 5186–5193.
152. W. E. Noland, J. E. Shudy, J. L. Rieger, Z. H. Tu, K. J. Tritch, Crystal structures of 2,6-di-bromo-4-methyl-benzo-nitrile and 2,6-di-bromo-4-methyl-phenyl isocyanide, *Acta Crystallographica Section E Crystallographic Communications* **2017**, *73*, 1913–1916.
153. X. Ji, W.-G. Cao, G. Zhao, Dual-reagent organophosphine catalyzed asymmetric Mannich reactions of isocyanoacetates with N -Boc-aldimines, *Tetrahedron* **2017**, *73*, 5983–5992.

154. E. Schwartz, H. J. Kitto, R. de Gelder, R. J. Nolte, A. E. Rowan, J. J. Cornelissen, Synthesis, characterisation and chiroptical properties of ‘click’able polyisocyanopeptides, *J. Mater. Chem.* **2007**, *17*, 1876–1884.
155. Britton, D. P-Iso-cyano-benzo-nitrile. *Acta Crystallogr. Sect. E Struct. Rep. Online* **2002**, *58* (6), 637–639.
156. G. Chennakrishnareddy, G. Nagendra, H. P. Hemantha, U. Das, T. N. Guru Row, V. V. Sureshbabu, *Tetrahedron* **2010**, *66*, 6718–6724.
157. A. Hinz, A. Schulz, A. Villinger, Tunable Cyclopentane-1,3-diyls Generated by Insertion of Isonitriles into Diphosphadiazanediyls, *Journal of the American Chemical Society* **2015**, *137*, 9953–9962.
158. N. Fusetani, K. Yasumuro, H. Kawai, T. Natori, L. Brinen, J. Clardy, Kalihinene and isokalihinol B, cytotoxic diterpene isonitriles from the marine sponge Acanthella klethra, *Tetrahedron Letters* **1990**, *31*, 3599–3602.
159. K. M. Skodje, L. M. Hinkle, M. O. Miranda, K. R. Mann, D. E. Janzen, Nonisomorphous x-ray structures of tritylnitrile and tritylisonitrile, *Journal of Chemical Crystallography* **2012**, *42*, 972–980.
160. A. G. Zhdanko, V. G. Nenajdenko, Nonracemizable isocyanoacetates for multicomponent reactions, *The Journal of Organic Chemistry* **2008**, *74*, 884–887.
161. A. E. Carpenter, C. C. Mokhtarzadeh, D. S. Ripatti, I. Havrylyuk, R. Kamezawa, C. E. Moore, Arnold. L. Rheingold, J. S. Figueroa, Comparative Measure of the Electronic Influence of Highly Substituted Aryl Isocyanides, *Inorganic Chemistry* **2015**, *54*, 2936–2944.
162. W. P. Fehlhammer, S. Schrölkamp, M. Hoyer, H. Hartl, W. Beck, Alkaliisocyanacetate. Synthese und Struktur von [K(18-Krone-6)](O2CCH2NC), *Zeitschrift für anorganische und allgemeine Chemie* **2005**, *631*, 3025–3029.
163. M. Monim-ul-Mehboob, M. Ramzan, W. Zierkiewicz, M. Michalczyk, R. Mahmood, M. Altaf, S. Nadeem, M. Akhtar, S. Ahmad, Synthesis, Characterization, and DFT Investigation of a Zinc(II)–Silver(I) Bimetallic Complex, $[Zn(Dmen)_2\{Ag(CN)_2\}_2][Zn(Dmen)_2(H_2O)_2]\{Ag(CN)_2\}_2$ (Dmen = N,N′-Dimethylethylenediamine), *Russian Journal of Coordination Chemistry* **2018**, *44*, 198–206.
164. E.Mallah, Q.Abu-Salem, K.Sweidan, N.Kuhn, C.Maichle-Mossmer, M.Steimann, M.Strobele, M.Walker, Imidazolium Dicyanoargentates, *Zeitschrift fur Naturforschung,B:Chemical Sciences* **2011**.
165. S. E. Baillie, V. L. Blair, T. D. Bradley, W. Clegg, J. Cowan, R. W. Harrington, A. Hernán-Gómez, A. R. Kennedy, Z. Livingstone, E. Hevia, Isomeric and chemical consequences of the direct magnesiation of 1,3-benzoazoles using β-diketiminate-stabilized magnesium bases, *Chemical Science* **2013**, *4*, 1895.
166. X.Solans, M.Font-Altaba, J.L.Brianso, *Zeitschrift fur Kristallographie, Kristallgeomie, Krystallphysik, Kristallchemie* **1983**.
167. Z.-J. Lv, P. D. Engel, L. Alig, S. Maji, M. C. Holthausen, S. Schneider, Stabilizing Doubly Deprotonated Diazomethane: Isolable Complexes with CN and CN Radical Ligands, *Journal of the American Chemical Society* **2022**, *144*, 21872–21877.
168. V. N. Nemykin, S. V. Dudkin, M. Fathi-Rasekh, A. D. Spaeth, H. M. Rhoda, R. V. Belosludov, M. V. Barybin, robing Electronic Communications in Heteronuclear Fe-Ru-Fe Molecular Wires Formed by Ruthenium(II) Tetraphenylporphyrin and Isocyanoferrocene or 1,1′-Diisocyanoferrocene Ligands, *Inorganic Chemistry* **2015**, *54*, 10711–10724.
169. Zhang, Y.; Maverick, A. W. Preparation of an Isocyano-β-Diketone via Its Metal Complexes, by Use of Metal Ions as Protecting Groups. *Inorg. Chem.* **2009**, *48* (22), 10512–10518.
170. Zhang, D.; Zhang, L.; Chen, X.; Ni, Z. A Series of Trinuclear Sandwich-like Cyanide-Bridged Iron(III)-Manganese(II) Complexes: Synthesis, Crystal Structures, and Magnetic Properties. *Transit. Met. Chem.* **2011**, *36* (5), 539–544.
171. Garner, M. E.; Hohloch, S.; Maron, L.; Arnold, J. Carbon–Nitrogen Bond Cleavage by a Thorium-NHC-Bpy Complex. *Angew. Chem. Int. Ed.* **2016**, *55* (44), 13789–13792.
172. Bowmaker, G. A.; Lim, K. C.; Skelton, B. W.; White, A. H. Syntheses, Structures and Vibrational Spectroscopy of Some Adducts of Copper(I) Cyanide with Pyridine Bases. *Z. Für Naturforschung B* **2004**, *59* (11–12), 1264–1276.
173. Chika Nanzan, Tomomi Sakata, *CSD Communication* **2019**.
174. A. V. Chizmeshya, C. J. Ritter, T. L. Groy, J. B. Tice, J. Kouvetakis, Synthesis of Molecular Adducts of Beryllium, Boron, and Gallium Cyanides: Theoretical and Experimental Correlations between Solid-State and Molecular Analogues, *Chemistry of Materials* **2007**, *19*, 5890–5901.
175. W. C. Andersen, D. R. Mitchell, K. J. Young, X. Bu, V. M. Lynch, H. A. Mayer, W. C. Kaska, The Synthesis, X-ray Crystal Structure, and Molecular Structure of Rhenium Tetra- carbonylbromidetrimethylamineisocyanoborane, *Inorganic Chemistry* **1999**, *38*, 1024–1027.
176. D. V. Konarev, A. V. Kuzmin, A. F. Shestakov, S. S. Khasanov, R. N. Lyubovskaya, Coordination-induced metal-to-macrocycle charge transfer and effect of cations on reorientation of the CN ligand in the $\{SnL2Mac\}^{2-}$ dianions (L = CN−, OCN−, Im−; Mac = phthalo- or naphthalocyanine), *Dalton Transactions* **2019**, *48*, 4961–4972.

177. R. Bhaskaran, K. Ramalingam, G. Bocelli, A. Cantoni, C. Rizzoli, Steric and electronic effects of N-coordinated NC− and NCS− on NiS_2PN: synthesis, spectral and single crystal X-ray structural studies on N,N′-di-n-butyldithiocarbamate complexes of nickel(II) with phosphorus and nitrogen donor ligands, *Journal of Coordination Chemistry* **2008**, *61*, 1710–1719.
178. F. Liu, S. Wang, C.-L. Gao, Q. Deng, X. Zhu, A. Kostanyan, R. Westerström, F. Jin, S.-Y. Xie, A. A. Popov, et al., Mononuclear Clusterfullerene Single-Molecule Magnet Containing Strained Fused-Pentagons Stabilized by a Nearly Linear Metal Cyanide Cluster *Angewandte Chemie International Edition* **2017**, *56*, 1830–1834.
179. Bing Yan, *CSD Communication* **2004**.
180. Wrackmeyer, B.; Maisel, H. E.; Tok, O. L.; Milius, W.; Herberhold, M. NMR Spectroscopic Characterization of Isocyano- and 1, 1′-Diisocyanoferrocene. The Molecular Structure of Isocyanoferrocene. *Z. Für Anorg. Allg. Chem.* **2004**, *630* (12), 2106–2109.
181. Y.-P. Ren, L.-S. Long, R.-B. Huang, L.-S. Zheng, Crystallographic report: Chain-like crystal structure of [Ni(en)2Ag(CN)2][Ag(CN)2]*Applied Organometallic Chemistry* **2005**, *19*, 1070–1071.
182. A. G. Coutsolelos, A. Tsapara, D. Daphnomili, D. L. Ward, Pseudohalogeno-bonding of thallium(III) porphyrins, stabilization of isocyano-bonding. Crystal structure of Tl(TPP)NC complex, *Journal of the Chemical Society, Dalton Transactions* **1991**, 3413.
183. K. Škoch, I. Císařová, J. Schulz, U. Siemeling, P. Štěpnička, Synthesis and characterization of 1′-(diphenylphosphino)-1-isocyanoferrocene, an organometallic ligand combining two different soft donor moieties, and its Group 11 metal complexes† *Dalton Transactions* **2017**, *46*, 10339–10354.
184. C. J. Stevens, G. S. Nichol, P. L. Arnold, J. B. Love, Isocyanide and Phosphine Oxide Coordination in Binuclear Chromium Pacman Complexes*Organometallics* **2013**, *32*, 6879–6882.
185. H. Xu, B.-Y. Zhou, K. Yu, Z.-H. Su, B.-B. Zhou, Z.-M. Su, Copper cyanide polymers with controllable dimensions modulated by rigid and flexible bis-(imidazole) ligands: synthesis, crystal structure and fluorescence properties *CrystEngComm* **2019**, *21*, 1242–1249.
186. F. Ekkehardt Hahn, M. Tamm, Coordinated versus Noncoordinated Isocyanides: Synthesis of Complexes CH3C(CH2NC)x[CH2NC—Cr(CO)5]3-x(x=1, 2) and Crystal Structure of CH3C(CH2NC)2[CH2NC — Cr(CO)5], *Chemische Berichte* **1992**, *125*, 119–121.
187. A. Jana, I. Objartel, H. W. Roesky, D. Stalke, Dehydrogenation of LGeH by a Lewis N-Heterocyclic Carbene Borane Pair under the Formation of L′Ge and its Reactions with B(C6F5)3 and Trimethylsilyl Diazomethane: An Unprecedented Rearrangement of a Diazo-compound to an Isonitrile *Inorganic Chemistry* **2009**, *48*, 7645–7649.
188. C. Zhang, P. Yang, E. Zhou, X. Deng, G. Zi, M. D. Walter, Reactivity of a Lewis Base Supported Thorium Terminal Imido Metallocene toward Small Organic Molecules *Organometallics* **2017**, *36*, 4525–4538.
189. Takuya Hayakawa, Tomomi Sakata, *CSD Communication* **2017**.
190. Y.-C. Huang, H.-Y. Chen, C.-Y. Cheng, Y.-L. Tsai, M. Y. Chiang, S. C. Hsu, Stepwise and Self-Assembly Synthesis of Tetranuclear Iron–Thiolate–Diisocyanide Metallocyclophane Complexes, *Journal of the Chinese Chemical Society* **2016**, *64*, 94–102.
191. W.-H. Zhang, Y.-L. Song, Y. Zhang, J.-P. Lang, Binuclear Cluster-to-Cluster-Based Supramolecular Compounds: Design, Assembly, and Enhanced Third-Order Nonlinear Optical Performances of {[Et4N]2[MoOS3Cu2(μ-CN)]2·2aniline}n and {[Et4N]4[MoOS3Cu3CN(μ′-CN)]2(μ-CN)2}n*Crystal Growth & Design* **2007**, *8*, 253–258.
192. T. Tanase, C. Yamamoto, B. Kure, T. Nakajima, One-dimensional Anisotropic Metal–Organic Module Containing a Pt6 Chain Terminated with Redox Active Ferrocenyl Units *Chemistry Letters* **2014**, *43*, 913–915.
193. A. Kassymbek, D. G. Gusev, A. Dmitrienko, M. Pilkington, G. I. Nikonov, An Isolable Gallium-Substituted Nitrilimine and its Reactivity with B−H, Si−H and B−B Bonds, *Chemistry – A European Journal* **2021**, *28*,.
194. T. Bartolomäs, D. Lentz, I. Neubert, M. Röttger, Synthese und Koordinationschemie von Diisocyanmethan, *Zeitschrift für anorganische und allgemeine Chemie* **2002**, *628*, 863.
195. J. Buschmann, T. Bartolmäs, D. Lentz, P. Luger, I. Neubert, M. Röttger, Synthesis, Structure, and Coordination Chemistry of Diisocyanomethane *Angewandte Chemie* **1997**, *109*, 2466–2468.
196. G. Ballmann, H. Elsen, S. Harder, Alkene Transfer Hydrogenation with Alkaline-Earth Metal Catalysts, *Angewandte Chemie International Edition* **2019**, *58*, 15736–15741.
197. K. Yuvaraj, I. Douair, A. Paparo, L. Maron, C. Jones, Reductive Trimerization of CO to the Deltate Dianion Using Activated Magnesium(I) Compounds, *Journal of the American Chemical Society* **2019**, *141*, 8764–8768.
198. A. M. Champsaur, C. Mézière, M. Allain, D. W. Paley, M. L. Steigerwald, C. Nuckolls, P. Batail, Weaving Nanoscale Cloth through Electrostatic Templating *Journal of the American Chemical Society* **2017**, *139*, 11718–11721.
199. J.-Q. Zhao, D. Cai, J. Dai, M. Kurmoo, X. Peng, M.-H. Zeng, Heptanuclear brucite disk with cyanide bridges in a cocrystal and tracking its pyrolysis to an efficient oxygen evolution electrode *Science Bulletin* **2019**, *64*, 1667–1674.

200. J. C. Byun, C. H. Han, K. J. Kim, Synthesis and crystal structure of a new polymer built from a cyano nickel(II) oxa-azamacrocyclic complex*Inorganic Chemistry Communications* **2006**, *9*, 171–174.
201. Z.-J. Lv, M. Zhu, W. Liu, Z. Chai, J. Wei, W.-X. Zhang, The Titanocene Complex of Bis(trimethylsilyl)acetylene: Synthesis, Structure, and Chemistry, *Organometallics* **2021**, *40*, 3992–3998.
202. M. Hailmann, S. Z. Konieczka, A. Himmelspach, J. Löblein, G. J. Reiss, M. Finze, Carba-closo-dodecaborate Anions with Two Functional Groups: [1-R-12-HC≡C-closo-1-CB11H10]− (R = CN, NC, CO2H, C(O)NH2, NHC(O)H) *Inorganic Chemistry* **2014**, *53*, 9385–9399.
203. G. Indramahalakshmi, A. Thamaraichelvan, R. Gandhidasan, S. Athimoolam, S. Natarajan, Poly[[aqua-[mu]-cyanido-tetra-kis(ethyl-enediamine)dicopper(II)] [nona-[mu]-cyanido-dizincate(II)dicuprate(I)]]*Acta Crystallographica Section E Structure Reports Online* **2007**, *63*, m2160–m2160.
204.]M. A. Tetilla, M. C. Aragoni, M. Arca, C. Caltagirone, C. Bazzicalupi, A. Bencini, A. Garau, F. Isaia, A. Laguna, V. Lippolis, et al., Colorimetric response to anions by a "robust" copper(ii) complex of a [9]aneN3 pendant arm derivative: CN− and I− selective sensing, *Chemical Communications* **2011**, *47*, 3805.
205. J. F. Valliant, P. Schaffer, A new approach for the synthesis of isonitrile carborane derivatives.: Ligands for metal based boron neutron capture therapy (BNCT) and boron neutron capture synovectomy (BNCS) agents, *Journal of Inorganic Biochemistry* **2001**, *85*, 43–51.
206. J. Weiß, B. Theis, S. Metz, C. Burschka, C. Fonseca Guerra, F. M. Bickelhaupt, R. Tacke, Neutral Pentacoordinate Halogeno- and Pseudohalogenosilicon(IV) Complexes with a Tridentate Dianionic O,N,O or N,N,O Ligand: Synthesis and Structural Characterization in the Solid State and in Solution *European Journal of Inorganic Chemistry* **2012**, *2012*, 3216–3228.
207. X. He, C. Lu, D. Yuan, S. Chen, J. Chen, Synthesis and Crystal Structures of Four Cyanide-Bridged Coordination Polymers, *European Journal of Inorganic Chemistry* **2005**, *2005*, 2181–2188.
208. D. W. Agnew, I. M. DiMucci, A. Arroyave, M. Gembicky, C. E. Moore, S. N. MacMillan, A. L. Rheingold, K. M. Lancaster, J. S. Figueroa, Crystalline Coordination Networks of Zero-Valent Metal Centers: Formation of a 3-Dimensional Ni(0) Framework with m-Terphenyl Diisocyanides *Journal of the American Chemical Society* **2017**, *139*, 17257–17260.
209. Tao, L.; Feng, L.; Shi-wei, Z.; Cheng-tai, W. Synthesis, Structure and Antitumor Activities of [Ni(Dien)2][Ni(CN)4] Complex. *Wuhan Univ. J. Nat. Sci.* **2004**, *9* (2), 229–233.
210. T. C. Holovics, R. E. Robinson, E. C. Weintrob, M. Toriyama, G. H. Lushington, M. V. Barybin, The 2,6-Diisocyanoazulene Motif: Synthesis and Efficient Mono- and Heterobimetallic Complexation with Controlled Orientation of the Azulenic Dipole*Journal of the American Chemical Society* **2006**, *128*, 2300–2309.
211. R. Hayoun, D. K. Zhong, A. L. Rheingold, L. H. Doerrer, Gold(III) and Platinum(II) Polypyridyl Double Salts and a General Metathesis Route to Metallophilic Interactions, *Inorganic Chemistry* **2006**, *45*, 6120–6122.
212. S. C. N. Hsu, H. H. Z. Chen, I.-J. Lin, J.-J. Liu, P.-Y. Chen, Dinuclear copper(I) complexes of tris(3,5-dimethylpyrazol-1-yl)methane: Synthesis, structure, and reactivity *Journal of Organometallic Chemistry* **2007**, *692*, 3676–3684.
213. R.-F. Zhang, B. Zhao, H.-S. Wang, P. Cheng, Two novel 2-D homometallic cyano-bridged complexes: Synthesis, structures and fluorescent properties, *Inorganic Chemistry Communications* **2007**, *10*, 1226–1228.
214. M. Trivedi, Bhaskaran, G. Singh, A. Kumar, N. P. Rath, Synthesis, spectral and structural studies of silver and gold(I) complexes containing some symmetrical diphosphine ligands*Journal of Organometallic Chemistry* **2014**, *758*, 9–18.
215. Ulrich Flörke, *CSD Communication* **2015**.
216. J.Pickardt, G.-T.Gong, Crystal Structure of Bis(1-Aza-Bicylo[2.2.2]Octane)-Dicyano-Mercury(II), Hg(C7H13N)2(CN)2. *Z. Für Krist. - Cryst. Mater.* **1995**, *210* (9), 715–716.
217. C. Hu, W. C. Hodgeman, D. W. Bennett, Bis(1,2-bis(diphenylphosphino)ethane)tungsten(0) Complexes Containing Electron-Saturated Metal Centers and Singly-Coordinated Bridging Ligands, *Inorganic Chemistry* **1996**, *35*, 1621–1626.
218. A. S. Smirnov, A. S. Mikherdov, A. V. Rozhkov, R. M. Gomila, A. Frontera, V. Yu. Kukushkin, N. A. Bokach, Halogen Bond-Involving Supramolecular Assembly Utilizing Carbon as a Nucleophilic Partner of I···C Non-Covalent Interaction,*Chemistry – An Asian Journal* **2023**, *18*,.
219. P. H. Bentley, J. P. Clayton, M. O. Boles, R. J. Girven, Transformations using benzyl 6-isocyanopenicillanate, *Journal of the Chemical Society, Perkin Transactions 1* **1979**, 2455-2467.
220. S. J. Wratten, D. J. Faulkner, K. Hirotsu, J. Clardy, Diterpenoid isocyanides from the marine sponge *Hymeniacidon amphilecta, Tetrahedron Letters* **1978**, *19*, 4345–4348.
221. P. Siega, L. Randaccio, P. A. Marzilli, L. G. Marzilli, Metal Coordination by Sterically Hindered Heterocyclic Ligands, Including 2-Vinylpyridine, Assessed by Investigation of Cobaloximes, *Inorganic Chemistry* **2006**, *45*, 3359–3368.

222. A. S. Ionkin, W. J. Marshall, B. M. Fish, L. A. Howe, *Organometallics* **2010**, *29*, 4154–4158.
223. M. Xu, B. Kooij, T. Wang, J. H. Lin, Z. Qu, S. Grimme, D. W. Stephan, *Angewandte Chemie International Edition* **2021**, *60*, 16965–16969.
224. Kumaradhas, P.; Gupta, K. R.; Kotha, S.; Brahmachari, E.; Nirmala, K. A. Crystal Structure of 2,6-Diisocyano-1,2,3,5,6,7-Hexahydro-*s*-Indacene-2,6-Dicarboxylic Acid Diethylester. *Anal. Sci. X-Ray Struct. Anal. Online* **2008**, *24*, 65–66.
225. T. M. Lane, D. S. Grubisha, C. Hu, D. W. Bennett, The structure of trans-1,4-diisocyanocyclohexane in solution, in the solid state, and as a ligand bridging bulky tungsten(II) complexes, *Journal of Molecular Structure* **1994**, *328*, 133–144.
226. R. Hulme, The structure of p-di-isocyanobenzene, *Acta Crystallographica* **1952**, *5*, 144–144.
227. M. Colapietro, A. Domenicano, G. Portalone, I. Torrini, I. Hargittai, G. Schultz, *Journal of Molecular Structure* **1984**, *125*, 19–32.
228. Nobuhara, M.; Tazima, H.; Shudo, K.; Itai, A.; Okamoto, T.; Iitaka, Y. A Fungal Metabolite, Novel Isocyano Epoxide. *Chem. Pharm. Bull. (Tokyo)* **1976**, *24* (4), 832–834.
229. M. D. Hollingsworth, M. E. Brown, B. D. Santarsiero, J. C. Huffman, C. R. Goss, Template-directed synthesis of 1–1 layered complexes of alpha, omega-dinitriles and urea-packing efficiency versus specific functional-group interactions, *Chemistry of Materials* **1994**, *6*, 1227–1244.
230. R. Jena, F. Benner, F. Delano, D. Holmes, J. McCracken, S. Demir, A. L. Odom, A rare isocyanide derived from an unprecedented neutral yttrium(ii) bis(amide) complex, *Chemical Science* **2023**, *14*, 4257–4264.
231. N.Shaikh, A.Panja, S.Goswami, P.Banerjee, M.Kubiak, Z.Ciunik, M.Puchalska, J.Legendziewicz, *Indian Journal of Chemistry* **2004**.
232. S. Ferlay, P. Dechambenoit, N. Kyritsakas, M. W. Hosseini, Molecular tectonics: tuning the dimensionality and topology of extended cyanocuprate networks using a bisamidinium cation, *Dalton Transactions* **2013**, *42*, 11661.
233. M. E. Garner, S. Hohloch, L. Maron, J. Arnold, Carbon–Nitrogen Bond Cleavage by a Thorium-NHC-bpy Complex, *Angewandte Chemie International Edition* **2016**, *55*, 13789–13792.
234. L. Reguera, A. Cano, J. Rodríguez-Hernández, D. G. Rivera, E. V. Van der Eycken, D. Ramírez-Rosales, E. Reguera, Cu^{I} Cu^{II} and Ag^{I}: P -isocyanobenzoates as novel 1D semiconducting coordination oligomers *Dalton Transactions* **2020**, *49*, 12432–12440.
235. L. Barluzzi, L. Chatelain, F. Fadaei-Tirani, I. Zivkovic, M. Mazzanti, Facile N-functionalization and strong magnetic communication in a diuranium(v) bis-nitride complex, *Chemical Science* **2019**, *10*, 3543–3555.
236. J. Buschmann, T. Bartolmäs, D. Lentz, P. Luger, I. Neubert, M. Röttger, Synthesis, Structure, and Coordination Chemistry of Diisocyanomethane, *Angewandte Chemie International Edition in English* **1997**, *36*, 2372–2374.
237. A. Hervé, Y. Bouzidi, J.-C. Berthet, L. Belkhiri, P. Thuéry, A. Boucekkine, M. Ephritikhine, U^{III}–CN versus U^{IV}–NC Coordination in Tris(silylamide) Complexes, *Inorganic Chemistry* **2015**, *54*, 2474–2490.
238. W. Shen, Z. Hu, P. Yu, Z. Wei, P. Jin, Z. Shi, X. Lu, An experimental and theoretical study of LuNC@C7$_{6,82}$ revealing a cage-cluster selection rule , *Inorganic Chemistry Frontiers* **2020**, *7*, 4563–4571.
239. V. Vreshch, W. Shen, B. Nohra, S.-K. Yip, V. W.-W. Yam, C. Lescop, R. Réau, *Chemistry - A European Journal* **2011**, *18*, 466–477.
240. Shinde, N.; Handa, R.; Furutachi, H.; Sakata, Y.; Akine, S.; Fujinami, S.; Suzuki, M. Synthesis and Crystal Structure of (μ-Acetato)Bis(μ-Alkoxo)Dicobalt(II, III) Complex with an Unsymmetric Dinucleating Ligand. *X-Ray Struct. Anal. Online* **2021**, *37*, 13–14.
241. Yanamoto, Y.; Aoki, K.; Yamazaki, H. A Novel High Nuclearity Platinum Isocyanide Cluster. *Chem. Lett.* **1979**, *8* (4), 391–392.
242. Y.-C. Huang, W.-Y. Lan, W.-M. Ching, S. C. Hsu, Formation of iron(iii)–thiolate metallocyclophane using a ferrocene-based bis-isocyanide, *New Journal of Chemistry* **2020**, *44*, 18242–18249.
243. Q. Liu, S. Yue, Z. Yan, Y. Xie, H. Cai, *Jiegou Huaxue* **2022**, 41387–41394.
244. The pandas development team, 2023, DOI 10.5281/zenodo.8092754.
245. C. R. Harris, K. J. Millman, S. J. van der Walt, R. Gommers, P. Virtanen, D. Cournapeau, E. Wieser, J. Taylor, S. Berg, N. J. Smith, et al., Array programming with NumPy, *Nature* **2020**, *585*, 357–362.
246. M. Waskom, seaborn: statistical data visualization, *Journal of Open Source Software* **2021**, *6*, 3021.
247. J. D. Hunter, Matplotlib: A 2D Graphics Environment, *Computing in Science & Engineering* **2007**, *9*, 90–95.
248. P. Virtanen, R. Gommers, T. E. Oliphant, M. Haberland, T. Reddy, D. Cournapeau, E. Burovski, P. Peterson, W. Weckesser, J. Bright, et al., SciPy 1.0: fundamental algorithms for scientific computing in Python, *Nature Methods* **2020**, *17*, 261–272.
